# TMOD2 and DOCK4 as Novel Gut Microbiota-Associated Biomarkers for Colorectal Adenoma: Integrated Transcriptomic Analysis and Therapeutic Target Identification

**DOI:** 10.1155/mi/6267309

**Published:** 2025-12-02

**Authors:** Chuang Liu, Li Wang, Jianguo Huai, Song He, Qiang Su, Qiuming Min, Zhenxiang An

**Affiliations:** ^1^Department of Gastroenterology, First Affiliated Hospital of Guizhou University of Traditional Chinese Medicine, Guiyang, China; ^2^Department of Pathology, Wuhu No. 1 People's Hospital, Wuhu, China; ^3^Guizhou University of Traditional Chinese Medicine, Guiyang, China; ^4^Department of Traditional Chinese Medicine Classics, First Affiliated Hospital of Guizhou University of Traditional Chinese Medicine, Guiyang, China

**Keywords:** biomarker discovery, colorectal adenoma, gut microbiome, immune infiltration, molecular targets, single-cell RNA sequencing, transcriptomics

## Abstract

Colorectal adenomas (CRA) represent critical precursors to colorectal cancer (CRC), yet reliable transcriptomic biomarkers for early detection and therapeutic targeting remain limited. Integration of gut microbiota (GM) genetics with transcriptomics offers a novel approach to identify disease-associated molecular signatures. We sought to identify GM-associated molecular signatures that could serve as early intervention targets. We integrated transcriptomic data with Mendelian randomization (MR) analysis to establish causal relationships between GM and CRA development. Machine learning algorithms identified robust biomarkers, which we validated through expression analysis and receiver operating characteristic (ROC) analysis to construct predictive nomogram models. Comprehensive molecular characterization included Gene Set Enrichment Analysis (GSEA), immune profiling, and regulatory network analysis. Single-cell RNA sequencing (scRNA-seq) analysis further validated biomarker expression patterns across distinct cell populations in the tumor microenvironment. We discovered 12 GM species with significant causal relationships to CRA risk. Two biomarkers, TMOD2 and DOCK4, emerged as powerful predictive indicators with strong correlation (*r* = 0.66, *p*  < 0.001). These biomarkers demonstrated excellent diagnostic performance in ROC analysis and revealed previously unrecognized connections to cell adhesion pathways critical for adenoma progression. Single-cell analysis revealed TMOD2 expression across multiple cell clusters with notable exclusion in mast cells, while DOCK4 expression was predominantly restricted to fibroblasts, myeloid, and epithelial cells. Notably, we identified distinct immune cell infiltration patterns, including altered naive B cells and macrophage populations, suggesting immune dysregulation as a key mechanism. GSEA revealed enrichment in cell adhesion molecule (CAM) pathways. Regulatory network analysis uncovered complex control by 18 microRNAs (miRNAs), 40 long noncoding RNAs (lncRNAs), and 10 transcription factors (TFs), with EIF3A emerging as a key m6A reader protein. Drug screening identified 22 potential therapeutic compounds, with trichostatin A showing optimal binding affinity. These findings establish TMOD2 and DOCK4 as novel biomarkers linking GM dysbiosis to CRA development, opening new avenues for microbiome-targeted early intervention strategies.

## 1. Introduction

Colorectal cancer (CRC) remains the third most common malignancy worldwide, with colorectal adenomas (CRA) serving as its primary precancerous gateway. While affecting 20%–30% of adults globally, these seemingly benign lesions harbor a troubling secret: 10%–15% will inevitably progress to invasive carcinoma [1]. Despite advances in colonoscopic screening and polypectomy techniques, recent studies continue to report concerning recurrence rates, highlighting the persistent challenges in adenoma management [2]. This underscores our incomplete understanding of the molecular mechanisms driving adenoma progression and recurrence.

The human gut microbiota (GM) has emerged as an unexpected orchestrator of colorectal tumorigenesis. This complex microbial ecosystem, containing over 1000 species and 100 trillion organisms, fundamentally shapes intestinal homeostasis through intricate host–microbe crosstalk [3]. Accumulating evidence reveals that CRA patients harbor distinctly altered microbial landscapes—characterized by inflammatory bacterial overgrowth and diminished protective butyrate producers [4, 5]. Yet a critical gap persists: do these microbial changes causally drive adenoma formation, or merely reflect disease consequences?

GM influences host gene expression through multiple interconnected pathways. Microbial metabolites, particularly short-chain fatty acids (SCFAs), can directly modulate epigenetic modifications including DNA methylation and histone acetylation, leading to altered transcriptional programs [6]. Additionally, microbial components such as lipopolysaccharides activate pattern recognition receptors (PRRs), triggering inflammatory signaling cascades that reshape host transcriptomes. These microbiota–host interactions may contribute to colorectal adenoma pathogenesis by disrupting normal epithelial homeostasis and immune surveillance mechanisms, providing biological rationale for identifying GM-associated transcriptomic signatures.

Traditional observational studies cannot resolve this chicken-and-egg dilemma due to inherent confounding factors and reverse causality. Mendelian randomization (MR) offers an elegant solution by leveraging genetic variants as natural randomization tools, effectively mimicking randomized controlled trials while circumventing ethical constraints [7]. Recent advances in microbial genome-wide association studies (MiGWASs) now enable MR analysis of specific microbial taxa, opening unprecedented opportunities to dissect causal microbe-disease relationships [8].

The advent of single-cell RNA sequencing (scRNA-seq) has revolutionized our understanding of cellular heterogeneity in complex tissues, enabling detailed characterization of gene expression patterns across distinct cell populations within the tumor microenvironment [9]. This technology provides unprecedented resolution to validate biomarker expression at the cellular level and uncover cell-type-specific regulatory mechanisms.

Here, we employ an integrative approach combining MR analysis with transcriptomic profiling and single-cell sequencing validation to identify GM-associated biomarkers in CRA development. By establishing causal relationships between specific microbial taxa and adenoma risk, followed by comprehensive molecular characterization of associated genetic signatures and single-cell validation of biomarker expression patterns, we aim to uncover novel therapeutic targets that could transform CRA prevention strategies.

## 2. Methods

### 2.1. Dataset Acquisition and Characteristics

#### 2.1.1. Transcriptomic Data Sources

We systematically retrieved CRA transcriptomic datasets from the Gene Expression Omnibus (GEO) database (https://www.ncbi.nlm.nih.gov/geo/). Two high-quality datasets were selected based on sample size, platform consistency, and data completeness: GSE37364 and GSE8671, both utilizing the Affymetrix Human Genome U133 Plus 2.0 Array platform (GPL570). The GSE37364 dataset comprised colon tissue samples from 29 CRA patients and 38 matched controls, while GSE8671 included 32 CRA cases and 32 controls. All samples underwent rigorous quality control assessment, with batch effects evaluated and normalized using standard protocols.

#### 2.1.2. Data Quality Control and Preprocessing

For transcriptomic datasets, quality control was performed using the following criteria: (1) samples with > 20% missing expression values were excluded; (2) genes with mean expression <1 fragments per kilobase of transcript per million mapped reads (FPKM) across all samples were filtered out; (3) outlier samples identified through hierarchical clustering and principal component analysis (PCA) were removed (samples beyond three standard deviations from the mean). Batch effects were assessed using ComBat-seq algorithm and corrected when significant (*p*  < 0.05 in Kruskal–Wallis test). Expression data normalization included: (1) log_2_ transformation of raw counts; (2) quantile normalization across samples; (3) *Z*-score standardization for cross-platform comparability. For genome-wide association study (GWAS) summary statistics, we ensured: (1) *λ* (genomic inflation factor) <1.1 for population stratification control; (2) minimum sample size of 1000 participants; (3) exclusion of variants with minor allele frequency (MAF) <0.01 or imputation quality (INFO) score <0.8.

#### 2.1.3. GM Genetic Data

GM genetic association data were obtained from the MiBioGen Consortium (https://mibiogen.gcc.rug.nl/), representing the largest available GWAS of human GM composition. This comprehensive dataset encompasses 18,340 individuals of predominantly European ancestry across 24 cohorts, ensuring robust statistical power for downstream analyses. The taxonomic classification included 211 microbial features hierarchically organized into 131 genera, 35 families, 20 orders, 16 classes, and 9 phyla, with genetic associations mapped across 14,587 single-nucleotide polymorphisms (SNPs).

#### 2.1.4. Disease Outcome Data

CRA-associated genetic data (identifier: ukb-b-14210) were retrieved from the IEU OpenGWAS database (https://gwas.mrcieu.ac.uk/), derived from the UK Biobank. This extensive dataset comprised 1391 CRA cases and 461,542 controls, with genome-wide association signals across 9,851,867 SNP loci, providing sufficient statistical power for robust MR analysis.

#### 2.1.5. Data Integration and Quality Control

All datasets underwent stringent quality control procedures, including assessment of population stratification, linkage disequilibrium patterns, and instrument strength validation. Only participants of European ancestry were included to minimize population stratification bias in MR analyses.

#### 2.1.6. Data Availability and Licensing

All datasets utilized in this study are publicly accessible through their respective repositories. The GEO database, MiBioGen Consortium, and IEU OpenGWAS database provide open access to research data under standard academic use terms, facilitating reproducible research and data sharing within the scientific community.

#### 2.1.7. Ethical Considerations

Since this study exclusively utilized publicly available datasets, no additional ethical approval was required. All original studies contributing data to the GEO database, MiBioGen Consortium, and UK Biobank had obtained appropriate ethical approvals and informed consent from participants at the time of data collection. Our secondary analysis of these deidentified, publicly accessible datasets complies with established guidelines for the responsible use of shared genomic and transcriptomic data.

### 2.2. MR Analysis

#### 2.2.1. Study Design and Assumptions

We conducted two-sample MR analysis to investigate causal relationships between GM composition and CRA development. GM taxonomic features served as exposure variables, with CRA as the outcome. Our analysis adhered to three fundamental MR assumptions: (1) instrumental variables (IVs) demonstrate robust association with the exposure; (2) IVs influence the outcome exclusively through the exposure pathway (exclusion restriction); (3) IVs remain independent of confounding factors affecting the exposure–outcome relationship.

#### 2.2.2. IV Selection and Quality Control

Valid IVs were extracted from GM GWAS summary statistics using the extract_instruments function from the TwoSampleMR R package (version 0.6.4) [10]. We implemented stringent screening criteria to ensure instrument validity: (1) genome-wide significant association with GM features (*p*  < 1 × 10^−5^) [11, 12]; (2) independence ensured through linkage disequilibrium clumping (*r*^2^ < 0.001, clumping window = 10 kb); (3) absence of direct association with CRA outcome (proxy variants allowed: rsq = 0.8); (4) instrument strength validation using *F*-statistics > 10, calculated as *F*=*R*^2^(*N* − *K* − 1)/*K*(1 − *R*^2^), where *R*^2^ represents cumulative explained variance, *N* denotes sample size, and *K* indicates SNP count; (5) exclusion of palindromic SNPs and GM features with fewer than three valid instruments; (6) MAF threshold > 0.01 to ensure adequate statistical power. Steiger directionality test to confirm causal direction. Significant heterogeneity (*p*  < 0.05) led to outlier SNP removal and reanalysis.

#### 2.2.3. MR Analysis Implementation

Effect alleles and effect sizes were harmonized across exposure and outcome datasets using the harmonise_data function. We employed five complementary MR methods to ensure robust causal inference: inverse variance weighted (IVW) as the primary method, supplemented by MR–Egger, weighted median, simple mode, and weighted mode approaches. Statistical significance was defined as *p*  < 0.05, with odds ratios (ORs) > 1 indicating risk-enhancing effects and OR < 1 suggesting protective associations (95% confidence intervals [CIs] excluding unity).

#### 2.2.4. Visualization and Result Interpretation

Scatter plots were generated using mr_scatter_plot to visualize SNP-exposure and SNP-outcome effect relationships. Forest plots (mr_forest_plot) illustrated individual SNP contributions to overall causal estimates, while funnel plots (mr_funnel_plot) assessed potential violations of MR assumptions through symmetry evaluation.

#### 2.2.5. Sensitivity Analysis and Validation

Comprehensive sensitivity analyses were performed to validate result robustness: (1) Cochran's *Q* heterogeneity test (mr_heterogeneity, *p* > 0.05 indicating homogeneity); (2) MR–Egger intercept test for horizontal pleiotropy (mr_pleiotropy_test, *p* > 0.05 indicating absence of pleiotropy); (3) leave-one-out analysis (mr_leaveoneout) to identify influential SNPs; (4) Steiger directionality test (directionality_test) to confirm correct causal direction (*p*  < 0.05 with correct_causal_direction = TRUE). Only GM features passing all sensitivity tests were considered to have robust causal relationships with CRA.

#### 2.2.6. Gene Mapping and Functional Annotation

Significant GM-associated SNPs were mapped to their corresponding genes using the SNPense database (https://biit.cs.ut.ee/gprofiler/snpense). These causal genes were subsequently integrated with transcriptomic analysis to identify convergent biological pathways underlying GM-CRA associations.

### 2.3. Identification of GM-Associated Candidate Genes in CRA

#### 2.3.1. Differential Expression Analysis and Cross-Dataset Validation

To identify transcriptomic signatures associated with GM-mediated CRA development, we performed differential gene expression analysis on both GSE37364 and GSE8671 datasets using the limma package (version 3.54.0) [13]. After standard preprocessing including background correction, quantile normalization, and batch effect assessment, differentially expressed genes (DEGs) were identified using stringent criteria: |log_2_ fold change (FC)| > 1.0 and false discovery rate (FDR) < 0.05 after Benjamini–Hochberg correction. To enhance reproducibility, only genes consistently dysregulated across both datasets were retained for downstream analysis. Results were visualized using volcano plots (ggplot2 v3.5.1) [14] highlighting the top 10 most significantly up and downregulated genes, and expression heatmaps (ComplexHeatmap v2.21.1) [15] displaying clustering patterns across samples.

#### 2.3.2. Integration With MR-Derived Causal Genes

GM-associated candidate genes were identified through intersection analysis between: (1) genes mapped from GM-associated SNPs identified through MR analysis (Section 2.2) and (2) consistently DEGs from transcriptomic analysis. Venn diagrams (VennDiagram v1.7.3) [16] visualized overlapping gene sets, with genes present in the intersection designated as high-priority candidates. This integrative approach ensures identification of targets with convergent evidence from both genetic causality and functional dysregulation in CRA, enhancing biological plausibility and therapeutic potential of identified candidates.

### 2.4. Biomarker Identification and Validation

#### 2.4.1. Feature Selection Using Machine Learning Approaches

To identify robust biomarkers from GM-associated candidate genes, we employed two complementary machine learning algorithms for feature selection. Least absolute shrinkage and selection operator (LASSO) regularization was performed using the glmnet package (version 4.1.8) [17] with 10-fold cross-validation on the GSE37364 dataset to identify the most predictive gene subset while preventing overfitting. The optimal lambda parameter was determined by minimizing cross-validation error, and genes with non-zero coefficients in the final model were retained as LASSO-selected features. Simultaneously, support vector machine-recursive feature elimination (SVM-RFE) was implemented using the caret package (version 6.0.94) [18] to identify the optimal feature subset that maximized classification accuracy through iterative backward elimination. The intersection of features identified by both algorithms (VennDiagram v1.7.3) [19] yielded candidate biomarkers with enhanced robustness and reduced algorithm-specific bias.

#### 2.4.2. Biomarker Validation and Performance Assessment

Candidate biomarkers underwent rigorous validation across both transcriptomic datasets. Differential expression was assessed using Wilcoxon rank-sum tests (rstatix v0.7.2) (https://CRAN.R-project.org/package=rstatix) with expression patterns visualized through violin plots (ggplot2 v3.5.1)[14]. Diagnostic performance was evaluated using receiver operating characteristic (ROC) analysis (pROC v1.18.5) [20], with area under the ROC curve (AUC) values calculated for each biomarker in both GSE37364 and GSE8671 datasets. Final biomarkers were required to meet stringent criteria: (1) statistically significant differential expression in both datasets (*p*  < 0.05), (2) consistent expression direction across cohorts, and (3) AUC > 0.7 in both validation sets, indicating good discriminatory power. This multistep validation approach ensures identification of clinically relevant biomarkers with robust performance across independent patient cohorts.

### 2.5. Nomogram Development and Clinical Performance Assessment

#### 2.5.1. Nomogram Construction and Validation

To facilitate clinical translation of identified biomarkers, we developed a comprehensive diagnostic nomogram using the rms package (version 6.5.0) [21] based on the validated biomarker panel in the GSE37364 dataset. The nomogram integrates individual biomarker contributions into a unified scoring system, providing clinically interpretable risk predictions for CRA development. Model performance was rigorously assessed through multiple complementary approaches: calibration curves were generated using the calibrate function (rms v6.5.0) to evaluate agreement between predicted and observed probabilities across risk strata, while decision curve analysis (DCA) quantified the clinical utility of the nomogram by comparing net benefits against alternative strategies (treat-all and treat-none) across different probability thresholds. Additionally, ROC analysis was performed using the pROC package (version 1.18.5) to assess discriminatory capacity, with AUC > 0.7 considered indicative of acceptable diagnostic performance. This comprehensive evaluation framework ensures both statistical accuracy and clinical applicability of the developed diagnostic tool.

### 2.6. Functional Characterization of Biomarkers

#### 2.6.1. Genomic Localization and Molecular Interactions

To comprehensively characterize the functional properties of identified biomarkers, we performed multidimensional analysis beginning with chromosomal localization using the RCircos package (version 1.2.2) [22] to visualize genomic distribution patterns and identify potential clustering or structural features. Interbiomarker relationships were assessed through Spearman correlation analysis using the psych package (version 2.4.3) [23], with results visualized as scatter plots using the ggscatter function (ggpubr v0.6.0) (https://CRAN.R-project.org/package=ggpubr) to reveal coexpression patterns that might indicate shared regulatory mechanisms or functional pathways. Additionally, we conducted subcellular localization prediction by obtaining FASTA sequences from the NCBI database (https://www.ncbi.nlm.nih.gov/) and analyzing them through the mRNALocater database (http://bio-bigdata.cn/mRNALocater/), with results visualized using ggplot2 (version 3.5.1) to understand potential cellular compartment-specific functions.

#### 2.6.2. Functional Network Analysis

To explore broader functional contexts and identify potential therapeutic targets, we constructed comprehensive protein–protein interaction (PPI) networks using the GeneMANIA database (http://www.genemania.org) with default parameters, including a minimum interaction confidence score of 0.7. This analysis integrated our biomarkers with the top 20 functionally similar genes based on coexpression, genetic interactions, pathway membership, and PPIs, ensuring high-confidence functional relationships.

### 2.7. RNA Methylation Modification Analysis

#### 2.7.1. m6A Modification Site Prediction and Protein Identification

To investigate potential regulatory mechanisms of identified biomarkers through RNA methylation, we conducted comprehensive m6A modification analysis using multiple computational approaches. N6-methyladenosine (m6A) modification sites within biomarker transcripts were predicted using the SRAMP database (http://www.cuilab.cn/sramp/) with default generic parameters, providing both modification probability scores and secondary structure context for predicted sites. Subsequently, m6A-associated regulatory proteins (writers, readers, and erasers) potentially targeting our biomarkers were identified through the ENCORI database (https://starbase.sysu.edu.cn/), which integrates experimental evidence from multiple high-throughput sequencing datasets. Common m6A modification proteins shared among multiple biomarkers were identified using intersection analysis (VennDiagram v1.7.3) to focus on regulators with broad impact on the biomarker panel.

#### 2.7.2. Protein–RNA Interaction Prediction and Motif Analysis

The functional relevance of identified m6A proteins was assessed through binding affinity prediction using the RPISeq database (http://pridb.gdcb.iastate.edu/RPISeq/), which employs machine learning algorithms to calculate protein–RNA interaction probabilities and binding scores based on sequence and structural features. The m6A regulatory protein demonstrating the highest binding affinity to our biomarkers was selected for detailed motif analysis using the RNA-binding protein (RBP)suite database (http://www.csbio.sjtu.edu.cn/bioinf/RBPsuite/) to identify specific recognition sequences and binding site preferences. This integrated analysis framework provides insights into post-transcriptional regulatory networks that may modulate biomarker expression in CRA development, potentially revealing novel therapeutic targets for intervention through RNA methylation pathways.

### 2.8. Pathway Enrichment Analysis of Biomarkers

#### 2.8.1. Gene Set Enrichment Analysis (GSEA) Implementation

To elucidate the biological pathways and molecular mechanisms underlying biomarker functions in CRA pathogenesis, we performed comprehensive pathway enrichment analysis using GSEA. The Kyoto Encyclopedia of Genes and Genomes (KEGG) pathway gene sets (c2.cp.kegg.v7.5.1.symbols.gmt) were obtained from the Molecular Signatures Database (MSigDB, https://www.gsea-msigdb.org/gsea/msigdb) as the reference pathway collection. For each biomarker in the GSE37364 dataset, Spearman's rank correlation coefficients were calculated against all other genes using the psych package (version 2.4.3), generating ranked gene lists ordered by correlation strength. This approach identifies genes that exhibit similar or opposing expression patterns to each biomarker, providing biological context for pathway analysis.

#### 2.8.2. Pathway Identification and Statistical Assessment

GSEA was performed on the correlation-ranked gene lists using the ClusterProfiler package (version 4.8.3)[24] to identify significantly enriched biological pathways associated with each biomarker. The analysis employed stringent statistical criteria: |normalized enrichment score (NES)| > 1.0 and *p*  < 0.05, ensuring identification of pathways with both biological relevance and statistical significance. The NES accounts for gene set size and provides a standardized measure of pathway enrichment strength, while the *p*-value threshold controls for false positive discoveries. For each biomarker, the top five most significantly enriched pathways (ranked by *p*-value) were selected for detailed interpretation, providing insights into the key biological processes through which these biomarkers may contribute to CRA development and progression.

### 2.9. Immune Microenvironment Analysis

#### 2.9.1. Immune Cell Infiltration Profiling and Group Comparison

Given the established association between CRA development and immune system dysfunction, we performed comprehensive immune microenvironment analysis using the CIBERSORT algorithm to quantify infiltration levels of 22 distinct immune cell types in both CRA and control groups from the GSE37364 dataset. Samples with CIBERSORT *p*-values > 0.05 were excluded to ensure reliable deconvolution results, as recommended by the algorithm developers. The resulting immune cell composition profiles were visualized using heatmaps generated with the pheatmap package (version 1.0.12) [24] to display overall infiltration patterns across samples. Statistical comparison of immune cell infiltration between CRA and control groups was performed using Wilcoxon rank-sum tests (*p*  < 0.05), with significantly different immune cell populations identified and visualized through box plots using the ggplot2 package (version 3.4.4) to highlight group-specific immune alterations.

#### 2.9.2. Immune-Biomarker Correlation Network Analysis

To explore functional relationships within the immune microenvironment and identify potential biomarker-immune interactions, we conducted Spearman correlation analysis using the psych package. Correlations were assessed both between differentially infiltrated immune cell types and between these immune cells and our identified biomarkers, with correlation coefficients |*r*| > 0.3 and *p*  < 0.05 considered statistically significant. The correlation results were presented as comprehensive heatmaps to visualize the complex network of immune–immune and immune-biomarker relationships.

### 2.10. Molecular Regulatory Network Construction

#### 2.10.1. Multilevel Regulatory Element Prediction

To comprehensively map the molecular regulatory landscape governing biomarker expression, we systematically identified multiple classes of regulatory elements through computational prediction approaches. MicroRNA (miRNA) regulators were predicted using complementary databases: miRDB (https://mirdb.org/) and TarBase (https://dianalab.e-ce.uth.gr/tarbasev9), with intersecting miRNAs retained to enhance prediction confidence by combining algorithm-based and experimentally validated targets. The identified miRNAs were subsequently used as query sequences in the STARBASE database (http://starbase.sysu.edu.cn/) to predict upstream long noncoding RNAs (lncRNAs) that may function as competing endogenous RNAs (ceRNAs) in the regulatory network. Additionally, transcription factors (TFs) directly regulating biomarker expression were identified through the JASPAR database (https://jaspar.elixir.no/), which provided both TF binding motifs and predicted binding sites within biomarker promoter regions.

#### 2.10.2. RNA–Protein Interaction Analysis and Network Integration

Given the critical role of post-transcriptional regulation in gene expression control, we investigated RBP interactions and pseudogene interference using the STARBASE database to map protein–RNA and RNA–RNA regulatory relationships that modulate biomarker mRNA stability and translation efficiency. These interactions are particularly relevant as RBPs can influence mRNA localization, splicing, and degradation, while pseudogenes may act as molecular sponges competing for regulatory factors. The comprehensive regulatory network encompassing biomarkers, miRNAs, lncRNAs, TFs, RBPs, and pseudogenes was visualized and analyzed using Cytoscape (version 3.9.1) [25], creating an integrated molecular regulatory map that illustrates the complex multilayered control mechanisms governing biomarker expression in CRA development.

### 2.11. Therapeutic Drug Prediction and Molecular Docking Analysis

#### 2.11.1. Drug Target Prediction and Network Construction

To identify potential therapeutic interventions targeting our biomarkers, we performed systematic drug prediction using the DSigDB database (https://dsigdb.tanlab.org/DSigDBv1.0/), which contains comprehensive drug–gene signature relationships derived from gene expression profiling experiments. Predicted drug–biomarker interactions were filtered to prioritize FDA-approved compounds to focus on clinically relevant therapeutic options with established safety profiles. The resulting drug–biomarker interaction network was visualized using Cytoscape (version 3.9.1) to identify key therapeutic nodes and potential multitarget drugs that could simultaneously modulate multiple biomarkers within our panel.

#### 2.11.2. Molecular Docking and Binding Affinity Assessment

To validate predicted drug–biomarker interactions and assess binding feasibility, we conducted molecular docking analysis for selected therapeutic compounds. Three-dimensional protein structures of biomarkers were retrieved from the UniProt database (https://www.uniprot.org/) in PDB format, while corresponding drug structures were obtained from the PubChem database (https://pubchem.ncbi.nlm.nih.gov/) in SDF format. Molecular docking simulations were performed using established computational protocols to generate binding poses and calculate docking scores, with scores below −5 kcal/mol indicating potentially favorable protein–drug interactions. The resulting docking complexes were visualized using PyMOL (version 3.1) [26] to examine binding modes, key residue interactions, and structural compatibility.

### 2.12. scRNA-seq Data Acquisition and Preprocessing

The scRNA-seq data utilized in this study were obtained from the GSE161277 dataset [27]. We utilized the scRNA-seq data of three normal tissues, four adenomas, and four carcinomas included in this dataset. We processed the data using the “Seurat” package in R. To ensure the quality of the data, cells with fewer than 200 genes or more than 2500 genes were filtered out, as were cells with a mitochondrial gene expression percentage exceeding 5%. This filtering was performed to remove potential doublets, dead cells, or cells with low transcriptome complexity. The remaining high-quality cells were then normalized using the “NormalizeData” function, and variable features were identified using the “FindVariableFeatures” function with the “vst” method. The data were scaled using the “ScaleData” function, and PCA was performed using the “RunPCA” function to reduce the dimensionality of the gene expression data. The first 20 principal components were assessed for their significance. Subsequently, the “FindNeighbors” function was used to compute the nearest neighbors for each cell, and clustering was performed using the “FindClusters” function. The Uniform Manifold Approximation and Projection (UMAP) was applied using the “RunUMAP” function to visualize the data in two dimensions. The “RunHarmony” function in the “Harmony” package was employed to correct for batch effects across different samples. Cell types were identified based on specific marker genes. The “FindAllMarkers” function was used to identify DEGs between clusters. These markers were then used to manually assign cell type labels to each cluster. The “DimPlot” function was used to visualize the final clustering results, and the “FeaturePlot” function was used to visualize the expression patterns of genes.

### 2.13. Statistical Analysis

All bioinformatics analyses were performed using R programing language (version 4.2.2) with appropriate statistical packages as specified in each section. Group comparisons between CRA and control samples were conducted using the Wilcoxon rank-sum test, while correlation analyses employed Spearman's rank correlation coefficient. Diagnostic model performance was evaluated using ROC analysis with AUC calculation, calibration plots, and DCA. GSEA utilized permutation-based testing with NES and FDR correction for multiple comparisons. Statistical significance was defined as *p*  < 0.05 for all analyses unless otherwise specified.

## 3. Results

### 3.1. Identification of GM With Causal Relationships to CRA

#### 3.1.1. Primary MR Analysis

**Figure 1:**
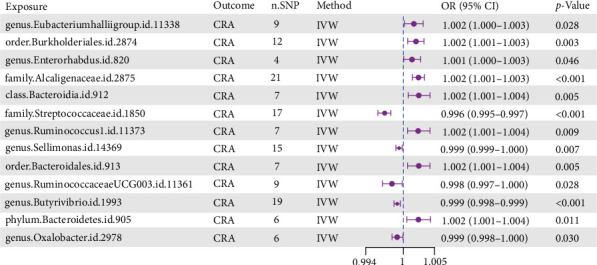
Causal associations between gut microbiota and colorectal adenoma risk identified through Mendelian randomization analysis. Forest plot depicting the causal effects of 12 gut microbiota taxa on colorectal adenoma (CRA) risk using inverse-variance weighted Mendelian randomization. Each point represents the odds ratio (OR) with 95% confidence intervals (CI) for individual bacterial taxa. Taxa to the right of the null line (OR = 1.0) represent risk factors, while those to the left represent protective factors. *p*-Values indicate statistical significance of causal associations. Horizontal bars represent 95% confidence intervals, and purple dots indicate point estimates of odds ratios.

Using the IVW method, we identified 12 GM taxa demonstrating significant causal associations with CRA risk (*p* < 0.05) (Figure 1, Supporting Information 1: Table S1). Among these, eight taxa were identified as risk factors (OR > 1.0, 95% CI excluding 1.0), while four taxa exhibited protective effects (OR < 1.0, 95% CI excluding 1.0). Scatter plot analysis revealed that positive slopes corresponded to risk-associated taxa, whereas negative slopes indicated protective taxa, with intercepts approximating zero, suggesting minimal confounding bias (Supporting Information 2: Figure S1). Effect size distributions confirmed that protective factors exhibited consistently negative SNP effects, while risk factors showed positive effects (Supporting Information 2: Figure S2). The symmetric and uniform distribution of SNPs across all taxa supported adherence to Mendel's second law of independent assortment (Supporting Information 2: Figure S3).

#### 3.1.2. Sensitivity Analysis and Causal Inference Validation

Comprehensive sensitivity analyses confirmed the robustness of our findings. Fixed-effect IVW analysis yielded nonsignificant results (*p* > 0.05) for all 12 taxa, indicating absence of directional pleiotropy (Supporting Information 1: Table S2). MR–Egger regression intercept tests demonstrated no horizontal pleiotropy across all associations (*p* > 0.05) (Supporting Information 1: Table S3). Leave-one-out analysis confirmed that no individual SNP disproportionately influenced the causal estimates, supporting result stability (Supporting Information 2: Figure S4). Steiger directionality tests validated the correct causal direction for all associations (*p*  < 0.01, correct causal direction = TRUE), excluding reverse causality bias (Supporting Information 1: Table S4). Gene mapping of the instrumental SNPs for these 12 causal GM taxa identified 33 host genes potentially mediating the microbiota-CRA causal pathway (Supporting Information 1: Table S5).

### 3.2. Identification and Validation of TMOD2 and DOCK4 as CRA Biomarkers

#### 3.2.1. Differential Expression Analysis and Candidate Gene Selection

**Figure 2:**
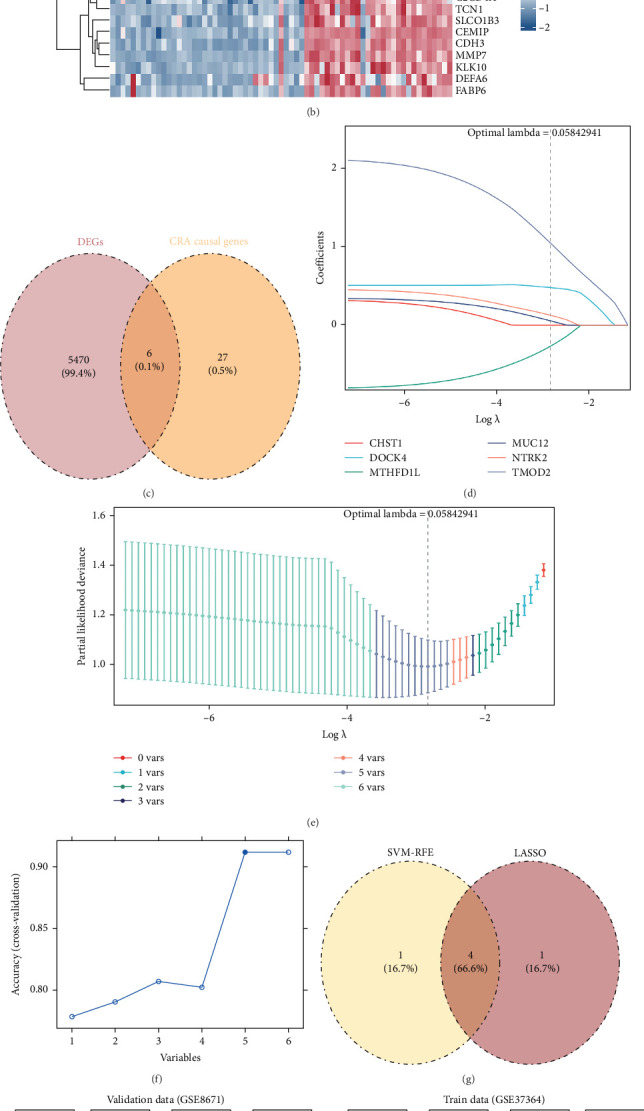
Identification and validation of key differentially expressed genes associated with colorectal adenoma. (A) Volcano plot displaying genome-wide differential gene expression between CRA and control samples. Red points indicate significantly upregulated genes, blue points indicate downregulated genes (adjusted *p*  < 0.05, |log_2_FC| > 1). Key genes are labeled. (B) Heatmap showing expression patterns of top differentially expressed genes (DEGs) across CRA and control samples, with hierarchical clustering revealing distinct molecular signatures between groups. (C) Venn diagram illustrating overlap between DEGs from our analysis (5478 genes) and known CRA causal genes (35 genes), identifying 8 shared candidates. (D) LASSO regression coefficient profiles showing variable selection process across different lambda values. (E) Cross-validation curve for LASSO model optimization, identifying optimal lambda value that minimizes prediction error. (F) Accuracy plot demonstrating model performance improvement with sequential addition of variables, reaching peak accuracy with 5 biomarkers. (G) Venn diagram comparing feature selection results from SVM-RFE (6 genes) and LASSO (5 genes), identifying 4 genes (TMOD2, DOCK4, NTRK2, MUC12) common to both methods. (H) Expression level comparison of the 4 candidate biomarkers between CRA and control groups in training datasets (GSE68947 and GSE37364), showing significant differential expression. (I) ROC curve analysis evaluating individual diagnostic performance of each biomarker in both training datasets (GSE68947 and GSE37364), with AUC values indicating discriminatory accuracy. *⁣*^*∗*^*p* < 0.05, *⁣*^*∗∗*^*p* < 0.01, *⁣*^*∗∗∗∗*^*p* < 0.0001.

Differential expression analysis of the GSE37364 dataset identified 5476 DEGs between CRA and control groups (|log_2_FC| > 0.5, *p*  < 0.05), comprising 2422 upregulated and 3054 downregulated genes in CRA samples (Figure 2A). The expression patterns of the most significantly altered genes are depicted in a heatmap showing the top 20 upregulated and downregulated DEGs (Figure 2B). Integration of the 5476 DEGs with the 33 host genes causally linked to GM-CRA associations yielded six candidate genes for further analysis (Figure 2C).

#### 3.2.2. Machine Learning-Based Feature Selection and Biomarker Identification

Two complementary machine learning approaches were employed for robust feature selection. LASSO regression analysis identified the optimal regularization parameter (*λ* = 0.05842941) that minimized model error, retaining five genes (TMOD2, DOCK4, MUC12, MTHFD1L, and NTRK2) with non-zero regression coefficients (Figure 2D,E). SVM-RFE achieved maximum classification accuracy with five features (TMOD2, DOCK4, CHST1, NTRK2, and MUC12) (Figure 2F). The intersection of both methods identified four consensus biomarker candidates: TMOD2, DOCK4, NTRK2, and MUC12 (Figure 2G).

#### 3.2.3. Biomarker Validation and Diagnostic Performance Assessment

All four candidate biomarkers demonstrated consistent downregulation patterns across both GSE37364 and GSE8671 datasets, with statistically significant differences between CRA and control groups (*p*  < 0.05) (Figure 2H). ROC analysis revealed that TMOD2 and DOCK4 achieved superior diagnostic performance with AUC values exceeding 0.8 in both validation datasets, indicating excellent discriminatory capacity for CRA detection (Figure 2I). Based on their robust performance across multiple validation criteria, TMOD2 and DOCK4 were designated as the primary biomarkers for subsequent analyses.

Among the 12 GM taxa with causal relationships to CRA, instrumental SNPs from genus Butyrivibrio (protective, OR = 0.999) and family Streptococcaceae (protective, OR = 0.996) were mapped to genomic regions containing TMOD2, while SNPs from genus *Eubacterium hallii* group (risk factor, OR = 1.002) and order Burkholderiales (risk factor, OR = 1.002) were linked to DOCK4 loci. The biological connection between these specific taxa and our identified genes is supported by their known roles in intestinal homeostasis: Butyrivibrio produces butyrate that regulates cytoskeletal organization genes like TMOD2, while Burkholderiales-associated inflammatory responses may modulate DOCK4 expression through Rac GTPase signaling pathways.

### 3.3. Construction and Validation of a High-Accuracy Predictive Nomogram

#### 3.3.1. Nomogram Development and Risk Assessment

**Figure 3:**
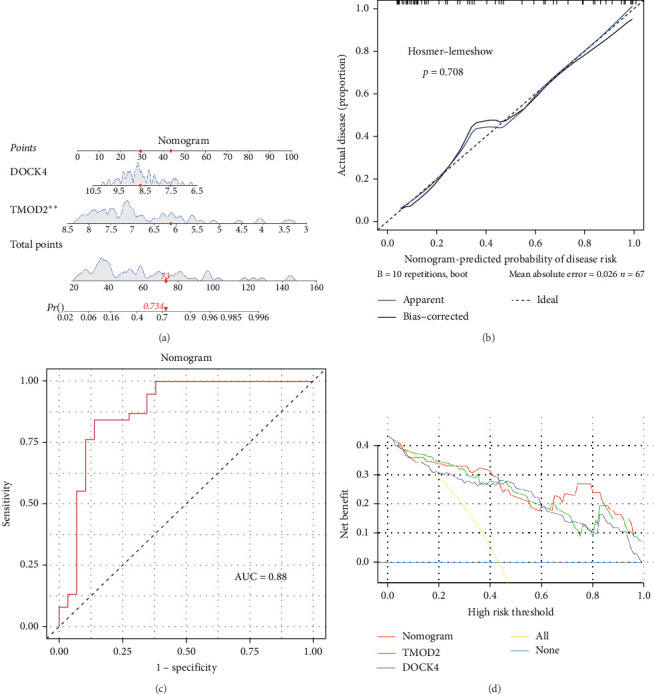
Development and validation of a high-accuracy nomogram for colorectal adenoma risk prediction. (A) Nomogram integrating TMOD2 and DOCK4 expression levels for individualized CRA risk assessment. Each biomarker contributes weighted points based on expression level, with total points corresponding to predicted disease probability. *⁣*^*∗∗*^*p* < 0.01 indicates statistical significance. Example calculation shows 73 total points corresponding to 73.4% CRA probability. (B) Calibration plot demonstrating agreement between nomogram-predicted probabilities and observed CRA outcomes. Hosmer–Lemeshow test (*p*=0.708) indicates excellent calibration with no significant deviation from the ideal prediction line (dashed diagonal). (C) Receiver operating characteristic (ROC) curve evaluating nomogram discriminatory performance, achieving an area under the ROC curve (AUC) = 0.88, indicating excellent diagnostic accuracy. (D) Decision curve analysis (DCA) comparing clinical utility of the nomogram versus individual biomarkers and default strategies. The nomogram (green line) demonstrates superior net benefit across most probability thresholds compared to TMOD2 alone (yellow), DOCK4 alone (blue), treat-all strategy (red), or treat-none strategy (gray).

A clinical nomogram integrating TMOD2 and DOCK4 expression levels was constructed to provide quantitative risk assessment for CRA diagnosis. The nomogram assigns weighted scores to each biomarker based on their contribution to disease risk, with the cumulative score corresponding to predicted CRA probability. For instance, a total score of 73 points corresponds to a 73.4% probability of CRA occurrence, demonstrating the model's capability for individualized risk stratification (Figure 3A).

#### 3.3.2. Model Calibration and Diagnostic Performance

Calibration analysis demonstrated excellent agreement between predicted and observed CRA probabilities, with the Hosmer–Lemeshow goodness-of-fit test yielding a nonsignificant *p*-value of 0.708 (*p* > 0.05), indicating no systematic deviation from perfect calibration (Figure 3B). The nomogram achieved superior discriminatory performance with an AUC of 0.88, substantially exceeding the threshold for excellent diagnostic accuracy (AUC > 0.8) (Figure 3C). DCA revealed that the nomogram consistently provided positive net benefit across most clinically relevant probability thresholds, with superior performance compared to individual biomarkers or default strategies of treating all or no patients (Figure 3D). These comprehensive validation metrics confirm that the TMOD2–DOCK4 nomogram represents a robust and clinically applicable tool for CRA risk prediction with high accuracy and clinical utility.

### 3.4. Chromosomal Localization and Functional Characterization of TMOD2 and DOCK4

#### 3.4.1. Genomic and Subcellular Localization Analysis

**Figure 4:**
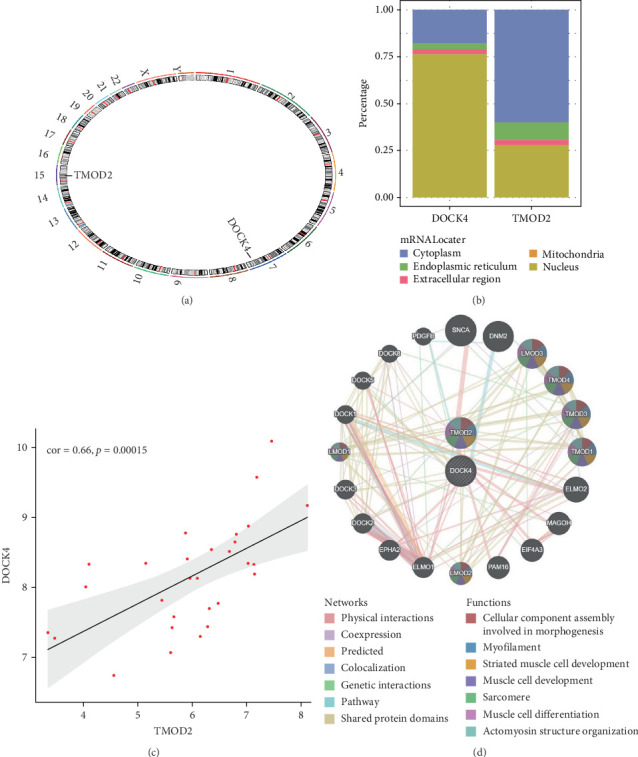
Chromosomal localization and functional characterization of TMOD2 and DOCK4 biomarkers. (A) Chromosomal ideogram showing genomic locations of TMOD2 (chromosome 15) and DOCK4 (chromosome 7). Red and blue markers indicate respective gene positions along chromosomal bands. (B) Subcellular localization prediction analysis using mRNALocater algorithm, showing probability distributions across cellular compartments. DOCK4 demonstrates predominant nuclear localization, while TMOD2 shows primary cytoplasmic distribution. (C) Correlation analysis between TMOD2 and DOCK4 expression levels revealing strong positive correlation (Pearson *r* = 0.66, *p*=0.00015). Gray shading represents 95% confidence interval of the regression line. (D) Protein–protein interaction (PPI) network constructed using STRING database, displaying functional relationships between TMOD2, DOCK4, and 20 associated proteins. Node colors represent different functional categories, while edge colors indicate interaction types (physical interactions, coexpression, predicted, colocalization, genetic interactions, pathway, and shared protein domains). Network analysis reveals enrichment in cellular component assembly, myofilament organization, and muscle cell development pathways.

Chromosomal mapping revealed that TMOD2 is located on human chromosome 15, while DOCK4 is positioned on chromosome 7, indicating their distinct genomic contexts (Figure 4A). Subcellular localization prediction using mRNALocater analysis demonstrated differential intracellular distributions: DOCK4 showed predominant nuclear localization (~75% probability), whereas TMOD2 exhibited primarily cytoplasmic distribution (~75% probability), with both genes showing additional presence in endoplasmic reticulum and mitochondrial compartments (Figure 4B). Despite their different subcellular localizations, expression correlation analysis revealed a strong positive correlation between TMOD2 and DOCK4 (Pearson correlation coefficient *r* = 0.66, *p*=0.00015), suggesting potential functional coordination or coregulation in CRA pathogenesis (Figure 4C).

#### 3.4.2. PPI Network and Functional Annotation

To elucidate the functional context of our biomarkers, we constructed a PPI network incorporating 20 functionally related genes, including key regulatory proteins, such as SNCA (alpha-synuclein) and dynamin-2 (DNM2; Figure 4D). Functional enrichment analysis revealed that the TMOD2–DOCK4 network is predominantly associated with cytoskeletal organization and muscle development processes, including cellular component assembly involved in morphogenesis, myofilament organization, striated muscle cell development, sarcomere assembly, and actomyosin structure organization. These functional associations suggest that TMOD2 and DOCK4 may contribute to CRA pathogenesis through disruption of cellular structural integrity and contractile apparatus organization, highlighting their potential roles in epithelial-mesenchymal transition and tissue remodeling processes characteristic of adenoma progression.

### 3.5. N6-Methyladenosine (m6A) Modification Analysis of TMOD2 and DOCK4

#### 3.5.1. m6A Modification Site Prediction and Structural Mapping

**Figure 5:**
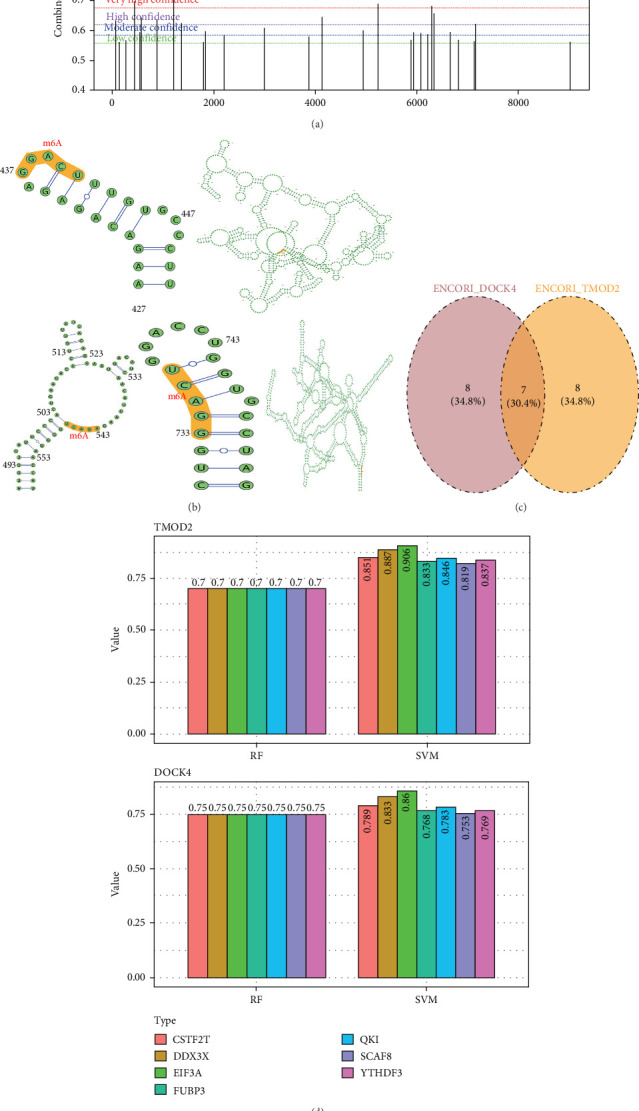
N6-methyladenosine (m6A) modification analysis and reader protein interactions for TMOD2 and DOCK4. (A) Prediction score distributions for m6A modification sites along TMOD2 and DOCK4 sequences. Confidence levels are color-coded (red: very high confidence, blue: high confidence, green: moderate confidence, gray: low confidence). Vertical lines indicate high-confidence m6A sites with scores exceeding threshold values. (B) RNA secondary structure predictions showing spatial locations of high-confidence m6A modification sites. TMOD2 m6A sites (positions 437–441) and DOCK4 sites (positions 543–547 and 733–737) are highlighted in orange circles within predicted stem-loop and single-strand regions. (C) Venn diagram illustrating overlap between predicted m6A reader proteins for TMOD2 and DOCK4, identifying seven shared proteins (QKI, YTHDF3, DDX3X, SCAF8, EIF3A, CSTF2T, and FUBP3) from 15 candidates each. (D) Comparative analysis of protein–RNA interaction prediction scores using random forest (RF) and support vector machine (SVM) classifiers. EIF3A demonstrates highest prediction confidence for both biomarkers across both algorithms. (E) RNA-binding protein motif analysis showing EIF3A binding site distributions across TMOD2 and DOCK4 transcripts. High-scoring binding sites (red peaks) indicate strong interaction potential, with quantitative binding site counts provided in Supporting Information 1: Table S6.

Computational analysis of N6-methyladenosine (m6A) modification sites revealed distinct methylation patterns for both biomarkers. TMOD2 contained three high-confidence m6A sites, while DOCK4 harbored four high-confidence modification sites based on prediction score distributions (Figure 5A). RNA secondary structure analysis localized the TMOD2 m6A sites to nucleotide positions 437–441, whereas DOCK4 m6A sites were mapped to positions 543–547 and 733–737, indicating strategic placement within functionally important RNA regions (Figure 5B). These modification sites predominantly occurred in stem-loop structures and single-stranded regions, suggesting potential roles in RNA stability and translation regulation.

#### 3.5.2. m6A Reader Protein Identification and Interaction Prediction

Systematic prediction identified 15 candidate m6A reader proteins for each biomarker, with intersection analysis revealing seven shared m6A-binding proteins: QKI, YTHDF3, DDX3X, SCAF8, EIF3A, CSTF2T, and FUBP3 (Figure 5C). Machine learning-based interaction prediction using both random forest (RF) and support vector machine (SVM) classifiers demonstrated high confidence scores for all seven proteins, with EIF3A achieving the highest prediction scores across both algorithms for interactions with both biomarkers (Figure 5D). Motif analysis using the RBPsuite database revealed that EIF3A exhibits extensive binding potential with both targets, with 130 predicted binding sites on TMOD2 and 120 sites on DOCK4 (Figure 5E, Supporting Information 1: Table S6). The high-density binding site distribution suggests that EIF3A may serve as a critical post-transcriptional regulator modulating both biomarkers through m6A-dependent mechanisms, potentially coordinating their expression in CRA pathogenesis.

### 3.6. GSEA of TMOD2 and DOCK4

#### 3.6.1. Pathway Enrichment Profiling and Biological Function Assessment

**Figure 6:**
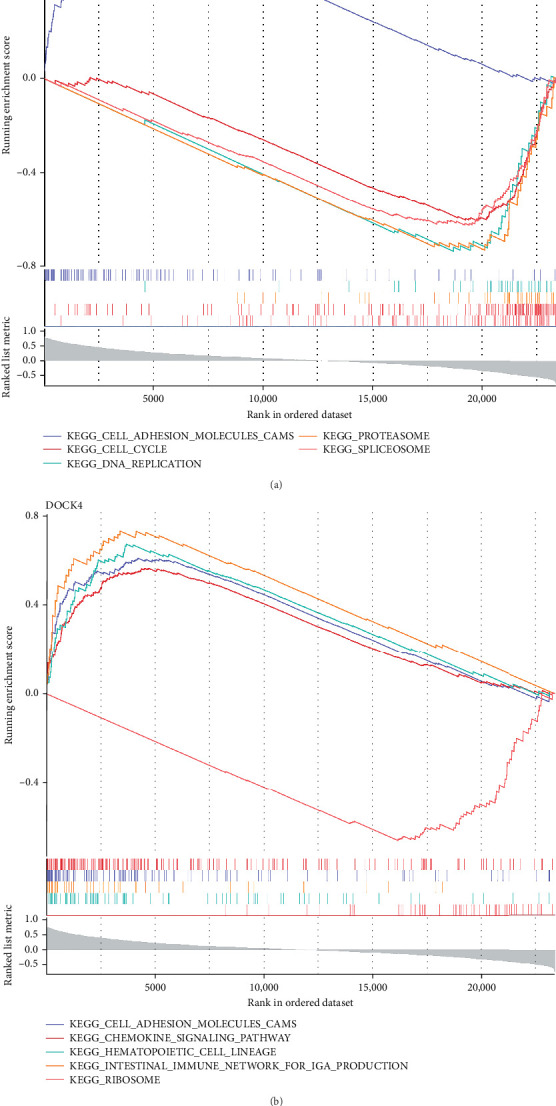
Gene Set Enrichment Analysis (GSEA) revealing distinct functional pathway profiles for TMOD2 and DOCK4. (A) GSEA enrichment plots for TMOD2 showing the top five most significantly enriched pathways. Running enrichment scores (colored lines) indicate pathway activity across ranked gene lists, with positive scores representing pathway upregulation. Key enriched pathways include spliceosome (purple), cell cycle (red), proteasome (orange), DNA replication (green), and cell adhesion molecules (CAMs, blue). The barcode plot below shows gene positions within the ranked dataset, and the gray curve represents the ranking metric correlation. (B) GSEA enrichment plots for DOCK4 displaying the most significantly enriched pathways, including ribosome (orange), hematopoietic cell lineage (blue), intestinal immune network for IgA production (purple), cell adhesion molecules (CAMs, green), and chemokine signaling pathway (red). Both biomarkers demonstrate convergent enrichment in CAMs pathway, suggesting shared roles in cell–cell communication and epithelial integrity maintenance. Complete pathway enrichment results are provided in Supporting Information 1: Table S7.

GSEA revealed distinct pathway enrichment profiles for both biomarkers, reflecting their diverse functional roles in CRA pathogenesis. TMOD2 demonstrated significant enrichment across 103 pathways (*p*  < 0.05), with the most significantly enriched pathways, including spliceosome (involved in RNA processing), cell cycle regulation, proteasome-mediated protein degradation, DNA replication machinery, and cell adhesion molecules (CAMs) signaling (Figure 6A, Supporting Information 1: Table S7). These enrichments suggest TMOD2's involvement in fundamental cellular processes, including transcriptional regulation, cell proliferation control, and intercellular communication networks.

#### 3.6.2. Comparative Pathway Analysis and Shared Functional Networks

DOCK4 exhibited enrichment in 98 significant pathways (*p*  < 0.05), predominantly associated with ribosome biogenesis and protein synthesis, hematopoietic cell lineage differentiation, intestinal immune network for IgA production, CAMs, and chemokine signaling pathways (Figure 6B, Supporting Information 1: Table S7). Notably, both biomarkers converged on the CAMs pathway, indicating a shared functional role in maintaining epithelial integrity and cell–cell communication. The differential enrichment patterns suggest complementary mechanisms: TMOD2 primarily regulating nuclear processes and cell cycle control, while DOCK4 modulating immune responses and cellular communication networks. This functional divergence, coupled with their convergence on CAMs signaling, supports their synergistic role in CRA development through disruption of normal epithelial homeostasis and immune surveillance mechanisms.

### 3.7. Immune Microenvironment Characterization and Biomarker-Immune Cell Interactions

#### 3.7.1. Immune Cell Infiltration Profiling in CRA

**Figure 7:**
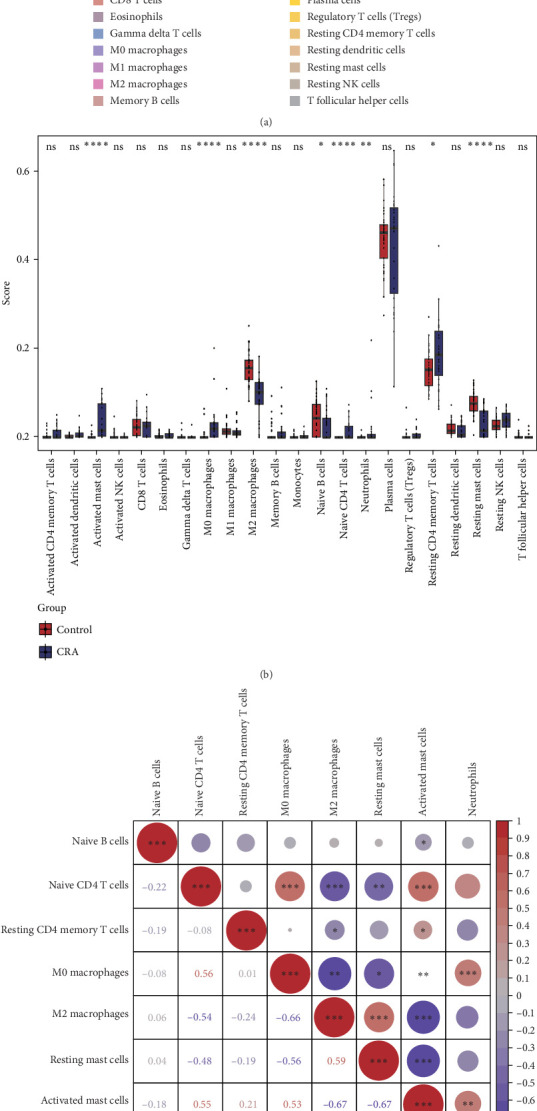
Immune microenvironment characterization and biomarker-immune cell associations in colorectal adenoma. (A) Stacked bar chart displaying immune cell composition profiles across individual samples from GSE37364 dataset. Each column represents one sample, with colors indicating relative proportions of 22 immune cell subtypes estimated using CIBERSORT algorithm. Plasma cells (yellow) constitute the predominant immune population in both CRA and control tissues. (B) Comparative analysis of immune cell infiltration levels between CRA and control groups. Box plots show significantly altered immune cell subtypes (*p*  < 0.05, Wilcoxon rank-sum test). Statistical significance levels: *⁣*^*∗*^*p*  < 0.05, *⁣*^*∗∗*^*p*  < 0.01, *⁣*^*∗∗∗*^*p*  < 0.001, *⁣*^*∗∗∗∗*^*p*  < 0.0001. CRA samples exhibit increased naive CD4^+^ T cells, resting CD4^+^ memory T cells, M0 macrophages, activated mast cells, and neutrophils, while showing decreased naive B cells, M2 macrophages, and resting mast cells. (C) Correlation matrix displaying inter-immune cell relationships. Circle size and color intensity represent correlation strength, with red indicating positive correlations and blue indicating negative correlations. Significant correlations include resting mast cells-M2 macrophages (*r* = 0.59) and activated mast cells-M2 macrophages (*r* = −0.67). (D) Biomarker-immune cell correlation analysis showing associations between DOCK4/TMOD2 expression and immune cell infiltration levels. DOCK4 demonstrates significant negative correlation with resting CD4^+^ memory T cells and positive correlation with M2 macrophages (*p*  < 0.001). Complete correlation results are provided in Supporting Information 1: Tables S8 and S9.

Comprehensive immune cell deconvolution analysis using the GSE37364 dataset revealed distinct immune microenvironment alterations between CRA and control tissues. The immune landscape was dominated by plasma cells in both groups, representing the predominant infiltrating immune population (Figure 7A). Comparative analysis identified significant differential infiltration of eight immune cell subtypes between CRA and control groups (*p*  < 0.05). Specifically, CRA tissues exhibited significant depletion of naive B cells, M2 macrophages, and resting mast cells, concurrent with enhanced infiltration of naive CD4^+^ T cells, resting CD4^+^ memory T cells, M0 macrophages, activated mast cells, and neutrophils (Figure 7B). This immune reprograming suggests a shift from anti-inflammatory (M2-dominated) to proinflammatory (M0/neutrophil-enriched) microenvironment during adenoma development.

#### 3.7.2. Immune Cell Interaction Networks and Biomarker Associations

Inter-immune cell correlation analysis revealed complex regulatory networks within the tumor microenvironment. Notable positive correlations were observed between resting mast cells and M2 macrophages (*r* = 0.59, *p*  < 0.001), while strong negative correlations existed between activated mast cells and both M2 macrophages and resting mast cells (*r* = −0.67, *p*  < 0.001), indicating antagonistic mast cell activation states (Figure 7C, Supporting Information 1: Table S8). Biomarker-immune cell association analysis demonstrated that DOCK4 expression negatively correlated with resting CD4^+^ memory T cells (*r* < −0.3, *p*  < 0.001) and positively associated with M2 macrophages (*r* > 0.3, *p*  < 0.001) (Figure 7D, Supporting Information 1: Table S9). These findings suggest that DOCK4 may serve as a molecular mediator linking epithelial cell dysfunction to immune microenvironment remodeling, potentially promoting M2 macrophage polarization while suppressing memory T cell responses in CRA pathogenesis.

### 3.8. Comprehensive Multimolecular Regulatory Network Analysis

#### 3.8.1. miRNA-lncRNA Regulatory Networks

**Figure 8:**
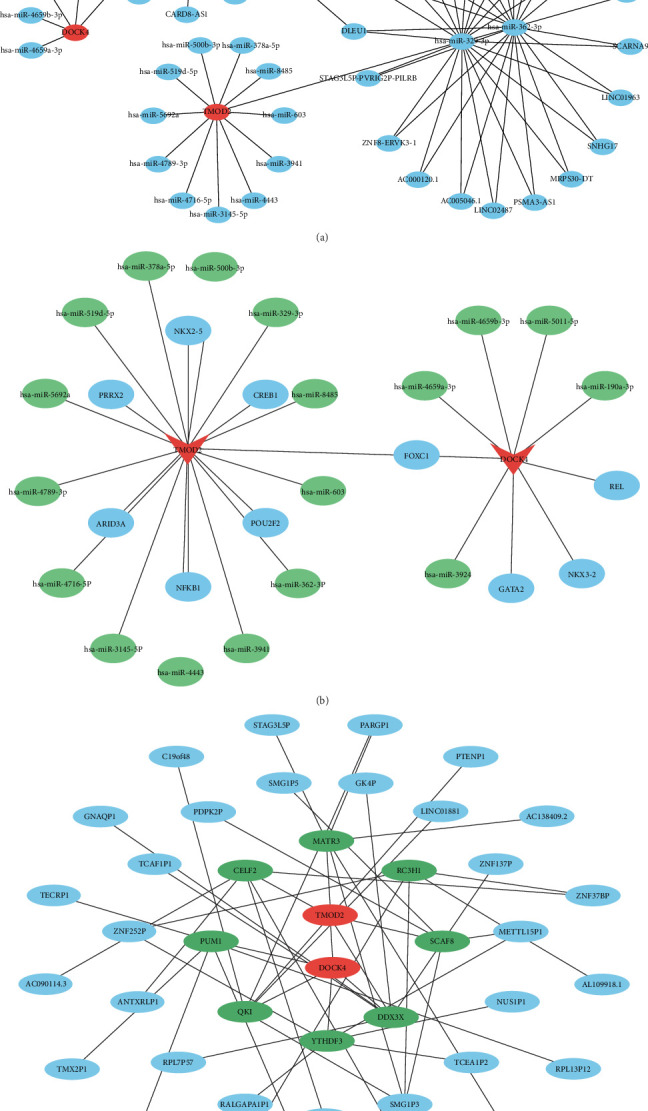
Comprehensive multimolecular regulatory networks governing TMOD2 and DOCK4 expression. (A) Competing endogenous RNA (ceRNA) regulatory network illustrating lncRNA-miRNA-mRNA interactions. Red nodes represent target genes (TMOD2 and DOCK4), blue nodes indicate miRNAs, and the network demonstrates complex post-transcriptional regulation through 60 nodes and 82 interactions. Key regulatory lncRNAs (NEAT1, KCNQ1OT1, SNHG14, and DLEU1) function as molecular sponges competing for miRNA binding. (B) Integrated transcriptional regulatory network combining transcription factors (TFs), miRNAs, and target genes. Green nodes represent TFs, blue nodes indicate miRNAs, and red nodes show biomarkers. FOXC1 emerges as a shared transcriptional regulator for both TMOD2 and DOCK4. The network contains 30 nodes and 29 regulatory relationships. (C) RNA-binding protein (RBP)-pseudogene regulatory network showing post-transcriptional control mechanisms. Green nodes represent pseudogenes, blue nodes indicate RBPs, and red nodes show target biomarkers. The network comprises 42 nodes and 50 interactions, with top-ranked RBPs based on pan-cancer expression profiles. This multilayered regulatory architecture demonstrates the complex molecular mechanisms controlling biomarker expression in CRA pathogenesis.

Systematic miRNA target prediction using miRDB and TarBase databases identified distinct regulatory profiles for both biomarkers. For DOCK4, 195 and 19 miRNAs were predicted respectively, yielding five consensus miRNAs following intersection analysis. Similarly, TMOD2 analysis revealed 334 and 49 predicted miRNAs respectively, with 13 overlapping candidates identified. Upstream lncRNA prediction through the starBase database identified 40 lncRNAs potentially regulating the 18 consensus miRNAs, with the majority being targeted by hsa-miR-3924, hsa-miR-362-3p, and hsa-miR-329-3p. Among these, NEAT1, KCNQ1OT1, SNHG14, and DLEU1 demonstrated high interaction frequencies, suggesting their roles as key regulatory hubs. The resulting competing endogenous RNA (ceRNA) network comprised 60 nodes and 82 interaction relationships, illustrating the complex post-transcriptional regulation of both biomarkers (Figure 8A).

#### 3.8.2. TF and RBP Regulatory Circuits

TF prediction identified four and seven potential regulators for DOCK4 and TMOD2, respectively, with FOXC1 emerging as a shared transcriptional regulator for both genes. Integration of 18 miRNAs, two biomarkers, and 10 TFs generated a comprehensive regulatory network containing 30 nodes and 29 interactions (Figure 8B). DNA-binding motif analysis revealed high-confidence binding sites for all 10 TFs, with elevated base stacking patterns indicating core regulatory regions critical for transcriptional control (Supporting Information 2: Figure S5). Additionally, RBP analysis predicted 109 and 51 RBPs for DOCK4 and TMOD2 respectively. Based on pan-cancer frequency rankings, the top five RBPs from each biomarker were selected for pseudogene prediction, identifying 32 pseudogenes regulated by eight RBPs. This multilayered regulatory network encompassed 42 nodes and 50 interactions, demonstrating the complex post-transcriptional and translational control mechanisms governing biomarker expression (Figure 8C).

### 3.9. Therapeutic Target Identification and Molecular Docking Validation

#### 3.9.1. Drug–Gene Interaction Network Construction and FDA-Approved Drug Prioritization

**Figure 9:**
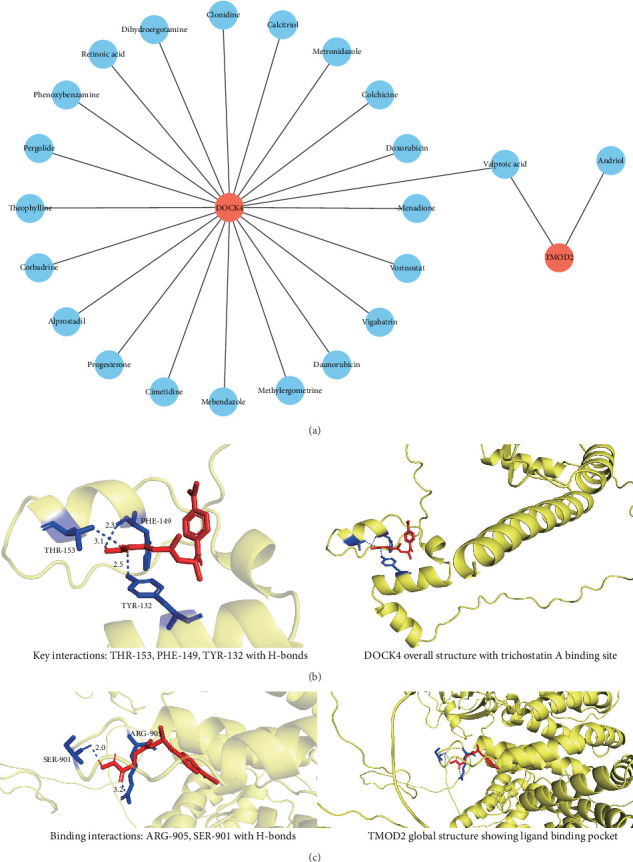
Drug target prediction and molecular docking analysis for therapeutic intervention strategies. (A) Drug–gene interaction network displaying FDA-approved compounds targeting TMOD2 and DOCK4. Red nodes represent biomarkers, blue nodes indicate drugs, with network edges showing 23 interaction relationships among 22 FDA-approved compounds. DOCK4 demonstrates extensive druggability (21 FDA-approved drugs) compared to TMOD2 (2 FDA-approved drugs). Three compounds (benzo[a]pyrene, trichostatin A, and valproic acid) exhibit dual-targeting potential for both biomarkers. (B) Molecular docking conformation of DOCK4 (yellow ribbon structure) in complex with trichostatin A (red stick representation). Key binding residues are highlighted in blue sticks (THR-153, PHE-149, and TYR-132) with binding affinity of −7.4 kcal/mol. Dashed lines indicate hydrogen bonds and favorable intermolecular interactions within the protein's binding pocket. (C) Molecular docking structure of TMOD2 (yellow ribbon) complexed with trichostatin A (red sticks), showing binding interactions with critical residues ARG-905 and SER-901 (blue sticks). The docking score of −6.7 kcal/mol indicates strong protein-ligand affinity. Both complexes demonstrate favorable binding geometries supporting trichostatin A as a dual-target therapeutic candidate for CRA treatment.

Comprehensive drug–gene interaction analysis using the DGIdb database revealed distinct therapeutic targeting profiles for both biomarkers. DOCK4 demonstrated extensive druggability with 74 predicted drug interactions, including 21 FDA-approved compounds, while TMOD2 showed more limited targeting potential with eight predicted drugs, of which two received FDA approval (Supporting Information 1: Table S10). The resulting drug–gene interaction network comprised 23 relationships between the biomarkers and 22 FDA-approved compounds, highlighting potential therapeutic intervention points for CRA treatment (Figure 9A). Notably, three compounds—benzo[a]pyrene, trichostatin A, and valproic acid—exhibited dual-targeting capability for both biomarkers, suggesting potential for combinatorial therapeutic approaches.

#### 3.9.2. Molecular Docking Analysis and Binding Affinity Assessment

Given the promising binding scores (< −5 kcal/mol) observed for valproic acid and benzo[a]pyrene with both targets, comprehensive molecular docking analysis was performed to evaluate trichostatin A interactions with TMOD2 and DOCK4 proteins. Molecular docking simulations revealed favorable binding affinities, with DOCK4-trichostatin A achieving a docking score of −7.4 kcal/mol and TMOD2-trichostatin A scoring −6.7 kcal/mol, both indicating strong protein-ligand interactions. Structural analysis of the DOCK4-trichostatin A complex revealed key binding interactions involving THR-153, PHE-149, and TYR-132 residues within the protein's active site, with the compound forming multiple hydrogen bonds and hydrophobic contacts (Figure 9B). Similarly, the TMOD2-trichostatin A interaction demonstrated stable binding through contacts with MET-95, SER-99, and surrounding hydrophobic residues (Figure 9C). These high-affinity interactions support trichostatin A as a promising dual-target therapeutic candidate for CRA intervention through simultaneous modulation of both biomarkers.

### 3.10. Single-Cell Transcriptional Analysis Reveals Expression and Distribution Patterns of TMOD2 and DOCK4 in Colorectal Carcinogenesis

**Figure 10:**
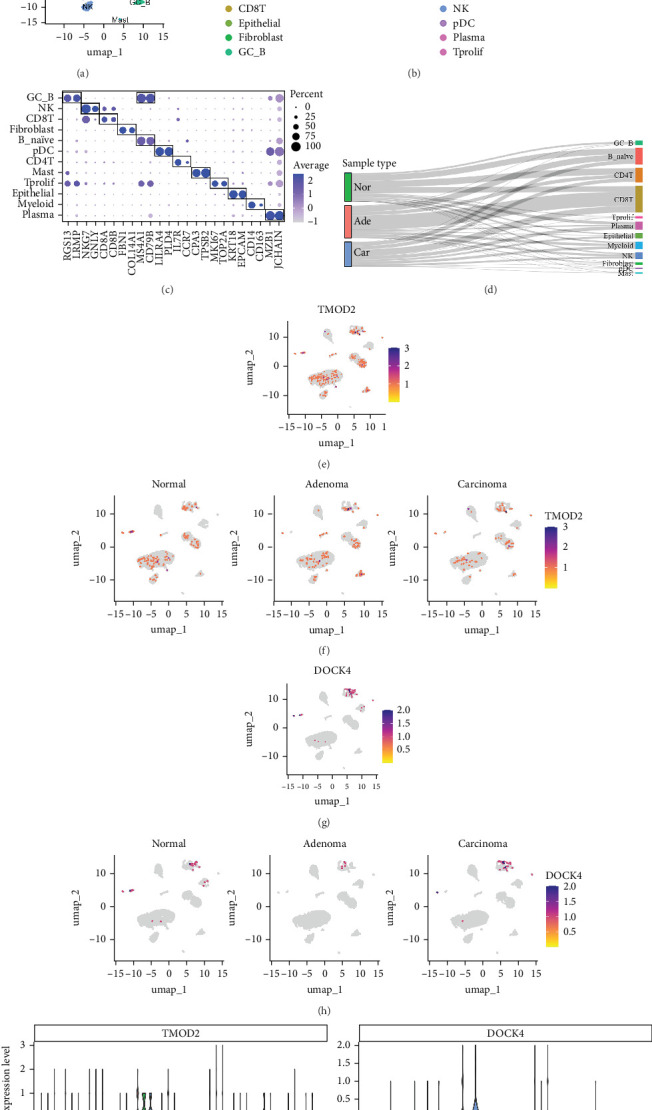
Expression and distribution patterns of TMOD2 and DOCK4 in Colorectal Adenoma in single-cell levels. (A) UMAP visualization of 12 distinct cell clusters identified from scRNA-seq analysis of intestinal tissues, including normal, adenoma, and carcinoma samples. The clusters include CD8T, CD4T, B_naive, Plasma, Myeloid, NK, Epithelial, GC_B, Tprolif, Fibroblast, Mast, and pDC. (B) UMAP visualizations depict the distinct cellular composition across different pathological states. (C) A dot plot provides a comprehensive overview of the expression landscape for markers specific to each cell cluster. (D) A Sankey diagram offers a visual representation of the proportional distribution and affiliation of these cell clusters to their respective sample types (normal, adenoma, and carcinoma). (E) Expression distribution of the TMOD2 across all the cell clusters. (F) Differential expression patterns of TMOD2 across normal, adenoma, and carcinoma samples. (G) Expression distribution of the DOCK4 across all the cell clusters. (H) Differential expression patterns of DOCK4 across normal, adenoma, and carcinoma samples. (I) The violin plots quantitatively compare the expression levels of TMOD2 and DOCK4 across various sample sources and cellular subgroups.

Subsequently, we performed scRNA-seq analysis of intestinal tissues, including normal, adenoma, and carcinoma samples from the GSE161277 dataset to reveal distinct cellular landscapes and to validate the expression patterns of the two genes. A total of 16,136 high-quality cells were included and we identified 12 distinct cell clusters including CD8T, CD4T, B_naive, Plasma, Myeloid, NK, Epithelial, Germinal center B cells (GC_B), Tprolif, Fibroblast, Mast, and pDC (Figure 10A). The UMAP visualizations highlight the distinct cellular composition across the different pathological states, with each cluster exhibiting unique spatial distribution patterns (Figure 10B). A dotplot provides a comprehensive overview of the expression landscape for markers specific to each cell cluster (Figure 10C). The Sankey diagram offers a visual representation of the proportional distribution and affiliation of these cell clusters to their respective sample types (Figure 10D). Figure 10E illustrates the expression distribution of the TMOD2 across all the cell clusters. We observed a broad expression profile of TMOD2 within the tumor microenvironment, with notable exclusion in the Mast cell cluster. Furthermore, the differential expression patterns of TMOD2 across normal, adenoma, and carcinoma samples were depicted in Figure 10F, which suggests its potential involvement in the progression of neoplastic transformation. In contrast, the expression of the DOCK4 gene is predominantly restricted to fibroblast, myeloid, and epithelial cells (Figure 10G). Figure 10H provides an overview of DOCK4 expression across different sample types (normal, adenoma, and carcinoma). The violin plots quantitatively compare the expression levels of TMOD2 and DOCK4 across various sample sources and cellular subgroups, and highlights the differential expression profiles of the two genes in the context of intestinal tissue pathology (Figure 10I).

Collectively, these findings validate the distinct expression patterns of TMOD2 and DOCK4 in colorectal carcinogenesis (from normal tissues to adenoma to cancer), and provide a molecular framework for understanding the role of the two genes in the pathogenesis of intestinal adenomas and carcinomas. The single-cell analysis confirms that TMOD2 demonstrates broader cellular distribution while DOCK4 shows more restricted expression patterns, supporting their complementary roles in different cellular compartments during CRA development.

## 4. Discussion

CRA represent precancerous lesions that constitute critical intervention points in preventing CRC progression, with emerging evidence highlighting the pivotal role of GM in adenoma pathogenesis. Through comprehensive bioinformatics analysis integrating transcriptomic profiling, immune infiltration assessment scRNA-seq validation, and molecular network construction, our investigation identified TMOD2 and DOCK4 as novel biomarkers for CRA diagnosis and therapeutic targeting. These findings establish a robust predictive nomogram achieving 88% diagnostic accuracy while elucidating the complex molecular mechanisms underlying adenoma development through multiomics characterization.

Our analysis revealed TMOD2 and DOCK4 as pivotal regulators in CRA pathogenesis, with both genes demonstrating significant diagnostic potential validated across multiple independent datasets and confirmed through single-cell analysis. TMOD2, encoding tropomodulin 2 and belonging to the tropomodulin family responsible for actin filament capping and cytoskeletal organization [28, 29], remains largely unexplored in colorectal neoplasia. Single-cell analysis revealed that TMOD2 exhibits broad expression across multiple cell types within the tumor microenvironment, with notable exclusion from mast cells, suggesting its fundamental role in maintaining cellular structural integrity across diverse cell populations. Our findings suggest TMOD2 contributes to CRA development through disruption of cell cycle regulation via cytoskeletal-mediated chromosome segregation control [30], enhancement of cellular migration and invasion through actin filament remodeling [31], and modulation of Wnt/*β*-catenin signaling pathways critical for colorectal tumorigenesis [32]. The involvement in immune microenvironment regulation [33] and genomic stability maintenance [34] further underscores TMOD2's multifaceted role in adenoma pathogenesis.

DOCK4, functioning as a guanine nucleotide exchange factor for Rac GTPases [35], demonstrates equally compelling involvement in CRA pathogenesis. Single-cell analysis revealed that DOCK4 expression is predominantly restricted to fibroblasts, myeloid cells, and epithelial cells, indicating its specialized role in specific cellular compartments critical for tissue homeostasis and immune regulation. Through Rac activation, DOCK4 promotes aberrant cell proliferation by accelerating G1/S phase transition [36], facilitates tumor cell migration and invasion via cytoskeletal remodeling [37], and potentially mediates epithelial-mesenchymal transition through transcriptional reprograming [38]. The regulation of VEGF-mediated angiogenesis [39] and immune evasion mechanisms [40] further highlights DOCK4's comprehensive involvement in creating a tumor-permissive microenvironment.

The convergent enrichment of both biomarkers in CAMs pathway underscores its fundamental importance in adenoma pathogenesis, where CAM dysregulation promotes cellular detachment and invasive behavior characteristic of neoplastic progression [41]. Cell cycle pathway enrichment aligns with the established paradigm of uncontrolled proliferation in adenoma development, where dysregulated cyclin-CDK complexes drive excessive cell division [42]. The concurrent involvement of DNA replication machinery suggests compromised genomic stability, potentially accelerating mutation accumulation and facilitating malignant transformation [43].

Spliceosome pathway enrichment reveals an underappreciated dimension of CRA pathogenesis, where aberrant mRNA processing generates oncogenic splice variants [44]. Similarly, proteasome pathway involvement indicates disrupted protein homeostasis, potentially affecting tumor suppressor degradation and oncogene stabilization [45]. The enrichment of chemokine signaling pathways connects our biomarkers to immune system modulation, where altered chemokine gradients may impair antitumor immunity while promoting protumorigenic inflammation [46].

The identification of EIF3A as the highest-affinity m6A reader protein for both biomarkers (130 binding sites on TMOD2 and 120 sites on DOCK4) suggests a coordinated post-transcriptional regulatory mechanism that may be critical for CRA development, providing an additional therapeutic target for intervention [47].

This MR analysis identified 12 GM taxa with significant causal relationships to CRA risk, establishing the genetic foundation for microbiota–host interactions in adenoma development. The protective effects of butyrate-producing bacteria (Butyrivibrio, family Streptococcaceae) and their genetic association with TMOD2 expression suggest that microbial metabolites may preserve epithelial integrity through cytoskeletal regulation. Conversely, the risk-associated taxa (*Eubacterium hallii* group, Burkholderiales) linked to DOCK4 expression may promote proinflammatory signaling through Rac GTPase pathways.

These findings support a model where GM dysbiosis causally contributes to CRA development by altering host transcriptomic programs. The convergence of microbial genetics and transcriptomic signatures on CAM pathways suggests that microbiota-mediated disruption of epithelial cell–cell communication represents a key mechanism in adenoma pathogenesis. This mechanistic framework has important implications for developing microbiota-targeted interventions to prevent CRA progression.

Our comprehensive immune profiling revealed significant alterations in eight immune cell populations, reflecting the complex interplay between neoplastic cells and host immunity. The depletion of naive B cells [48], alongside altered naive CD4^+^ T cells [49] and resting CD4^+^ memory T cells [50], indicates compromised adaptive immunity. The shift from M2 macrophages [51] to M0 macrophages [52] suggests loss of tissue repair functions and enhanced inflammatory responses. The activation status changes in mast cells, from resting [53] to activated states [54], along with increased neutrophil infiltration [55], collectively indicate a proinflammatory microenvironment conducive to tumor progression.

The strong positive correlation between resting mast cells and M2 macrophages, contrasted with negative correlations involving activated mast cells, reveals dynamic mast cell functional states in CRA development [56]. The significant association between DOCK4 expression and both resting CD4^+^ memory T cells and M2 macrophages suggests this biomarker actively shapes the immune landscape [57], potentially suppressing memory T cell responses while promoting immunosuppressive macrophage phenotypes. The single-cell analysis further validates these immune-biomarker interactions by demonstrating DOCK4's specific expression in myeloid cells, providing a cellular basis for its role in immune microenvironment modulation.

Our multilayered regulatory network analysis unveiled complex post-transcriptional control mechanisms governing biomarker expression. The identification of hsa-miR-3924, hsa-miR-362-3p, and hsa-miR-329-3p as key regulatory miRNAs, along with lncRNAs NEAT1, KCNQ1OT1, SNHG14, and DLEU1, establishes a competitive endogenous RNA network potentially dysregulated in CRA. These regulatory molecules have demonstrated significant roles in cancer progression: NEAT1 functions as an oncogenic lncRNA in multiple malignancies [58], while KCNQ1OT1 serves as a tumor promoter through various mechanisms [59]. SNHG14 exhibits oncogenic properties in CRC[60, 61], and DLEU1 demonstrates tumor suppressive functions [62]. The regulatory miRNAs also show disease-relevant functions: miR-362-3p affects cancer cell behavior [63], miR-329-3p influences tumor progression [64], and miR-3924 modulates cellular processes in cancer contexts [65].

Drug prediction analysis identified 22 FDA-approved compounds with therapeutic potential. Daunorubicin and doxorubicin, established anthracycline chemotherapeutics, show promise in CRC treatment [66, 67]. The HDAC inhibitor vorinostat demonstrates antiproliferative effects in colorectal neoplasia [68, 69], while valproic acid exhibits epigenetic modulation capabilities [70, 71]. Colchicine's anti-inflammatory and antimitotic properties suggest potential for adenoma chemoprevention [72]. Notably, trichostatin A demonstrated superior binding affinity to both targets, with established anticancer properties through HDAC inhibition, cell cycle arrest, and apoptosis induction [73, 74].

While our comprehensive bioinformatics approach enhanced by single-cell validation provides valuable insights into CRA pathogenesis, several limitations warrant consideration. The analysis relies heavily on computational predictions and public datasets, which may not fully capture the complexity of individual patient variations or tissue-specific expression patterns. However, the integration of scRNA-seq data significantly strengthens our findings by providing cellular-level validation of biomarker expression patterns. The identified biomarkers and regulatory networks require extensive experimental validation through cell culture studies, animal models, and expanded clinical cohorts to confirm their functional relevance and therapeutic potential.

Additionally, our pathway-level analysis, while comprehensive, represents relatively superficial coverage of each mechanistic aspect. Future investigations should prioritize mechanistic validation of the most promising findings, particularly the immune-biomarker interactions and drug sensitivity predictions. The integration of multiomics approaches, including proteomics, metabolomics, and expanded single-cell sequencing studies will provide deeper insights into the temporal dynamics of adenoma development and the precise roles of identified biomarkers in disease progression.

Despite these limitations, our findings establish TMOD2 and DOCK4 as novel biomarkers with significant diagnostic and therapeutic potential in CRA. The single-cell validation provides crucial confirmation of their cell-type-specific expression patterns and functional relevance across the tumor microenvironment. The comprehensive molecular characterization provides a foundation for targeted intervention strategies and personalized risk assessment, ultimately contributing to improved patient outcomes through early detection and precision medicine approaches.

## 5. Conclusions

Through integrative analysis combining MR of GM genetics with transcriptomics and scRNA-seq validation, we identified TMOD2 and DOCK4 as novel diagnostic biomarkers for CRA, achieving 88% diagnostic accuracy while revealing their fundamental roles in adenoma pathogenesis through cytoskeletal regulation, cell cycle control, and immune microenvironment modulation. Our comprehensive molecular characterization uncovered convergent pathway enrichment in CAMs, DNA replication, and chemokine signaling, alongside significant immune cell population shifts from anti-inflammatory M2 macrophages to proinflammatory states, establishing a mechanistic framework linking epithelial dysfunction to immune dysregulation. Single-cell analysis confirmed cell-type-specific expression patterns, with TMOD2 showing broad distribution across multiple cell types and DOCK4 demonstrating restricted expression in fibroblasts, myeloid, and epithelial cells, providing cellular-level validation of their complementary functional roles. The construction of multilayered regulatory networks involving key miRNAs, lncRNAs, and TFs provides critical insights into post-transcriptional control mechanisms, while drug prediction analysis identified 22 FDA-approved compounds with therapeutic potential, particularly trichostatin A demonstrating superior dual-targeting capabilities validated through molecular docking. These findings establish TMOD2 and DOCK4 as clinically actionable biomarkers that bridge the gap between molecular pathogenesis and therapeutic intervention, with single-cell validation confirming their cellular relevance, offering immediate opportunities for enhanced early detection, risk stratification, and precision medicine approaches in colorectal adenoma management, ultimately advancing our capacity to prevent CRC through targeted intervention at the precancerous stage.

## Data Availability

All data utilized in this study are publicly available through established repositories. Transcriptomic datasets can be accessed from the Gene Expression Omnibus (GEO) database: GSE37364 and GSE8671 (https://www.ncbi.nlm.nih.gov/geo/). Single-cell RNA sequencing data were obtained from GSE161277 (https://www.ncbi.nlm.nih.gov/geo/). Gut microbiota genetic association data are available from the MiBioGen Consortium (https://mibiogen.gcc.rug.nl/). Colorectal adenoma GWAS data were obtained from the IEU OpenGWAS database (identifier: ukb-b-14210, https://gwas.mrcieu.ac.uk/). All analysis code and supplementary data generated in this study are available from the corresponding author upon reasonable request. No custom datasets were generated that require additional data sharing arrangements. The complete bioinformatics pipeline and detailed analysis protocols are available in the supplementary methods section.

## Supporting Information

Supporting Information 1 Table S1. Complete Mendelian randomization results for 12 causal gut microbiota taxa with odds ratios and *p*-values. Table S2. Fixed-effect inverse-variance weighted analysis for directional pleiotropy testing. Table S3. MR–Egger regression intercept test results for horizontal pleiotropy evaluation. Table S4. Steiger directionality test results validating causal direction. Table S5. Gene mapping results for instrumental SNPs identifying 33 host genes mediating microbiota-CRA pathways. Table S6. RNA-binding protein motif analysis with EIF3A binding site distributions for TMOD2 and DOCK4. Table S7. Gene set enrichment analysis results for TMOD2 (103 pathways) and DOCK4 (98 pathways). Table S8. Inter-immune cell correlation matrix for 22 immune cell subtypes. Table S9. Biomarker-immune cell correlation analysis for TMOD2/DOCK4 expression. Table S10. Drug–gene interaction analysis for FDA-approved compounds targeting TMOD2 and DOCK4.

Supporting Information 2 Figure S1. SNP-exposure and SNP-outcome scatter plots for gut microbiota-CRA Mendelian randomization analysis. Figure S2. Effect size distribution plots for protective and risk-associated gut microbiota taxa. Figure S3. SNP distribution plots confirming Mendel's second law adherence. Figure S4. Leave-one-out sensitivity analysis plots for causal estimate stability. Figure S5. DNA-binding motif analysis and base stacking patterns for TMOD2 and DOCK4 regulatory regions.

Supporting Information 3 STROBE-MR checklist of recommended items to address in reports of Mendelian randomization studies.

## References

[1] Sung H., Ferlay J., Siegel R. L. (2021). Global Cancer Statistics 2020: GLOBOCAN Estimates of Incidence and Mortality Worldwide for 36 Cancers in 185 Countries. *CA: A Cancer Journal for Clinicians*.

[2] Wang Q.-P., He X.-X., Xu T., Ji W., Qian J.-M., Li J.-N. (2020). Polyp Recurrence After Colonoscopic Polypectomy. *Chinese Medical Journal*.

[3] Gagnière J., Raisch J., Veziant J. (2016). Gut Microbiota Imbalance and Colorectal Cancer. *World Journal of Gastroenterology*.

[4] Deng D., Zhao L., Song H. (2025). Microbiome Analysis of Gut Microbiota in Patients With Colorectal Polyps and Healthy Individuals. *Scientific Reports*.

[5] Wu X.-R., He X.-H., Xie Y.-F. (2025). Characteristics of Gut Microbiota Dysbiosis in Patients With Colorectal Polyps. *World Journal of Gastrointestinal Oncology*.

[6] Nshanian M., Gruber J. J., Geller B. S. (2025). Short-Chain Fatty Acid Metabolites Propionate and Butyrate are Unique Epigenetic Regulatory Elements Linking Diet, Metabolism and Gene Expression. *Nature Metabolism*.

[7] Chen J., Li S., Bu S. (2025). Associations of Physical Frailty and Polygenic Score With Incident Heart Failure in Cardiovascular Patients: Unraveling the Mediating Role of Inflammation. *GeroScience*.

[8] Kurilshikov A., Medina-Gomez C., Bacigalupe R. (2021). Large-Scale Association Analyses Identify Host Factors Influencing Human Gut Microbiome Composition. *Nature Genetics*.

[9] Sun J., Shi R., Zhou Z. (2025). Identification of CACNB1 Protein as an Actionable Therapeutic Target for Hepatocellular Carcinoma via Metabolic Dysfunction Analysis in Liver Diseases: An Integrated Bioinformatics and Machine Learning Approach for Precise Therapy. *International Journal of Biological Macromolecules*.

[10] Hemani G., Zheng J., Elsworth B. (2018). The Mr-Base Platform Supports Systematic Causal Inference Across the Human Phenome. *eLife*.

[11] Rühlemann M. C., Hermes B. M., Bang C. (2021). Genome-Wide Association Study in 8,956 German Individuals Identifies Influence of Abo Histo-Blood Groups on Gut Microbiome. *Nature Genetics*.

[12] Qin Y., Havulinna A. S., Liu Y. (2022). Combined Effects of Host Genetics and Diet on Human Gut Microbiota and Incident Disease in a Single Population Cohort. *Nature Genetics*.

[13] Ritchie M. E., Phipson B., Wu D. (2015). Limma Powers Differential Expression Analyses for Rna-Sequencing and Microarray Studies. *Nucleic Acids Research*.

[14] Gustavsson E. K., Zhang D., Reynolds R. H., Garcia-Ruiz S., Ryten M. (2022). Ggtranscript: An R Package for the Visualization and Interpretation of Transcript Isoforms Using Ggplot2. *Bioinformatics*.

[15] Gu Z., Eils R., Schlesner M. (2016). Complex Heatmaps Reveal Patterns and Correlations in Multidimensional Genomic Data. *Bioinformatics*.

[16] Chen H., Boutros P. C. (2011). VennDiagram: A Package for the Generation of Highly-Customizable Venn and Euler Diagrams in R. *BMC Bioinformatics*.

[17] Fang P., Sun T., Pandey A. K. (2023). Understanding Water Conservation vs. Profligation Traits in Vegetable Legumes Through a Physio-Transcriptomic-Functional Approach. *Horticulture Research*.

[18] Zhang Z., Zhao Y., Canes A., Steinberg D., Lyashevska O. (2019). Predictive Analytics With Gradient Boosting in Clinical Medicine. *Annals of Translational Medicine*.

[19] Noguera-Castells A., García-Prieto C. A., Álvarez-Errico D., Esteller M. (2023). Validation of the New EPIC DNA Methylation Microarray (900K EPIC v2) for High-Throughput Profiling of the Human DNA Methylome. *Epigenetics*.

[20] Jacobs J. P., Lagishetty V., Hauer M. C. (2023). Multi-Omics Profiles of the Intestinal Microbiome in Irritable Bowel Syndrome and its Bowel Habit Subtypes. *Microbiome*.

[21] Wu C., Luo Y., Chen Y., Qu H., Zheng L., Yao J. (2022). Development of a Prognostic Gene Signature for Hepatocellular Carcinoma. *Cancer Treatment and Research Communications*.

[22] Chong W., Ren H., Chen H. (2024). Clinical Features and Molecular Landscape of Cuproptosis Signature-Related Molecular Subtype in Gastric Cancer. *iMeta*.

[23] Zein R. A., Akhtar H. (2025). Getting Started With the Graded Response Model: An Introduction and Tutorial in R. *International Journal of Psychology*.

[24] Deng K.-G., Zhao H., Zuo P.-X. (2019). Association Between KIAA0319 SNPs and Risk of Dyslexia: A Meta-Analysis. *Journal of Genetics*.

[25] Qin M., Xing Y., Sun M. (2024). An Exploration of the Antioxidative and Anti-Inflammatory Role of Lactiplantibacillus Plantarum 106 Via Improving Mitochondrial Function. *Foods*.

[26] Fakih Z., Germain H. (2025). Implication of Ribosomal Protein in Abiotic and Biotic Stress. *Planta*.

[27] Zheng X., Song J., Yu C. (2022). Single-Cell Transcriptomic Profiling Unravels the Adenoma-Initiation Role of Protein Tyrosine Kinases During Colorectal Tumorigenesis. *Signal Transduction and Targeted Therapy*.

[28] Zhao M. L., Wang J. X., Bian X. K. (2023). Hexavalent Chromium Causes Centrosome Amplification by Inhibiting the Binding Between TMOD2 and NPM2. *Toxicology Letters*.

[29] Omotade O. F., Rui Y., Lei W. (2018). Tropomodulin Isoform-Specific Regulation of Dendrite Development and Synapse Formation. *The Journal of Neuroscience*.

[30] Gray K. T., Suchowerska A. K., Bland T. (2016). Tropomodulin Isoforms Utilize Specific Binding Functions to Modulate Dendrite Development. *Cytoskeleton*.

[31] Peng Y., Dong S., Yang Z. (2021). Identification of Docetaxel-Related Biomarkers for Prostate Cancer. *Andrologia*.

[32] Pascual-Alonso A., Xiol C., Smirnov D., Kopajtich R., Prokisch H., Armstrong J. (2024). Multi-Omics in MECP2 Duplication Syndrome Patients and Carriers. *European Journal of Neuroscience*.

[33] Wu X., Li C., Wang Z. (2022). A Bioinformatic Analysis Study of M7G Regulator-Mediated Methylation Modification Patterns and Tumor Microenvironment Infiltration in Glioblastoma. *BMC Cancer*.

[34] Majed S. O. (2022). RNA Sequencing-Based Total RNA Profiling; The Oncogenic MiR-191 Identification as a Novel Biomarker for Breast Cancer. *Cellular and Molecular Biology*.

[35] Li H., Wang M., Zhou H., Lu S., Zhang B. (2020). Long Noncoding RNA EBLN3P Promotes the Progression of Liver Cancer via Alteration of microRNA-144-3p/DOCK4 Signal. *Cancer Management and Research*.

[36] Qin T., Yang J., Huang D. (2021). DOCK4 Stimulates MUC2 Production Through its Effect on Goblet Cell Differentiation. *Journal of Cellular Physiology*.

[37] Huang L., Chambliss K. L., Gao X. (2019). SR-B1 Drives Endothelial Cell LdL Transcytosis via DOCK4 to Promote Atherosclerosis. *Nature*.

[38] Westbrook J. A., Wood S. L., Cairns D. A. (2019). Identification and Validation of DOCK4 as a Potential Biomarker for Risk of Bone Metastasis Development in Patients With Early Breast Cancer. *Journal of Pathology*.

[39] Yazbeck P., Cullere X., Bennett P. (2022). DOCK4 Regulation of Rho Gtpases Mediates Pulmonary Vascular Barrier Function. *Arteriosclerosis, Thrombosis, and Vascular Biology*.

[40] Zhao Q., Zhong J., Lu P. (2021). DOCK4 is a Platinum-Chemosensitive and Prognostic-Related Biomarker in Ovarian Cancer. *PPAR Research*.

[41] Hu Q., Saleem K., Pandey J., Charania A. N., Zhou Y., He C. (2023). Cell Adhesion Molecules in Fibrotic Diseases. *Biomedicines*.

[42] Glaviano A., Singh S. K., Lee E. H. C. (2025). Cell Cycle Dysregulation in Cancer. *Pharmacological Reviews*.

[43] Gu L., Hickey R. J., Malkas L. H. (2023). Therapeutic Targeting of DNA Replication Stress in Cancer. *Genes*.

[44] Li R., Chen J., Shen X. (2024). A Study of the Clinical Significance of mSEPT9 in Monitoring Recurrence and Prognosis in Patients With Surgically Treated Colorectal Cancer. *PLOS ONE*.

[45] Xia J., Xu M., Hu H. (2024). 5,7,4′-Trimethoxyflavone Triggers Cancer Cell PD-L1 Ubiquitin-Proteasome Degradation and Facilitates Antitumor Immunity by Targeting Hrd1. *MedComm*.

[46] Atretkhany K.-S. N., Drutskaya M. S., Nedospasov S. A., Grivennikov S. I., Kuprash D. V. (2016). Chemokines, Cytokines and Exosomes Help Tumors to Shape Inflammatory Microenvironment. *Pharmacology & Therapeutics*.

[47] Huo C., Wu D., Li X. (2024). eIf3a Mediates Malignant Biological Behaviors in Colorectal Cancer Through the PI3K/AKT Signaling Pathway. *Cancer Biology & Therapy*.

[48] Che L.-H., Liu J.-W., Huo J.-P. (2021). A Single-Cell Atlas of Liver Metastases of Colorectal Cancer Reveals Reprogramming of the Tumor Microenvironment in Response to Preoperative Chemotherapy. *Cell Discovery*.

[49] Shen J., Gong X., Tan S. (2025). CDK1 Acts as a Prognostic Biomarker Associated With Immune Infiltration in Pan-Cancer, Especially in Gastrointestinal Tumors. *Current Medicinal Chemistry*.

[50] Zhang S., Jiang Y., Shi L. (2024). Identification and Analysis of Key Genes Related to Efferocytosis in Colorectal Cancer. *BMC Medical Genomics*.

[51] Zhang M., Li X., Zhang Q., Yang J., Liu G. (2023). Roles of Macrophages on Ulcerative Colitis and Colitis-Associated Colorectal Cancer. *Frontiers in Immunology*.

[52] Zhang X., Yue L., Cao L. (2024). Tumor Microenvironment-Responsive Macrophage-Mediated Immunotherapeutic Drug Delivery. *Acta Biomaterialia*.

[53] Xie Z., Niu L., Zheng G. (2023). Single-Cell Analysis Unveils Activation of Mast Cells in Colorectal Cancer Microenvironment. *Cell and Bioscience*.

[54] Liu X., Li X., Wei H., Liu Y., Li N. (2023). Mast Cells in Colorectal Cancer Tumour Progression, Angiogenesis, and Lymphangiogenesis. *Frontiers in Immunology*.

[55] Khan U., Chowdhury S., Billah M. M., Islam K. M. D., Thorlacius H., Rahman M. (2021). Neutrophil Extracellular Traps in Colorectal Cancer Progression and Metastasis. *International Journal of Molecular Sciences*.

[56] Zhong J., Qin Y., Yu P. (2022). The Landscape of the Tumor-Infiltrating Immune Cell and Prognostic Nomogram in Colorectal Cancer. *Frontiers in Genetics*.

[57] Sun Y., Liu L., Fu Y. (2023). Metabolic Reprogramming Involves in Transition of Activated/Resting Cd4+ Memory T Cells and Prognosis of Gastric Cancer. *Frontiers in Immunology*.

[58] Shen X., Ye Z., Wu W. (2021). lncRNA NEAT1 Facilitates the Progression of Colorectal Cancer Via the KDM5A/Cul4A and Wnt Signaling Pathway. *International Journal of Oncology*.

[59] Duan Q., Cai L., Zheng K. (2020). lncRNA KCNQ1OT1 Knockdown Inhibits Colorectal Cancer Cell Proliferation, Migration and Invasiveness via thePI3K/AKT Pathway. *Oncology Letters*.

[60] Wang X., Yang P., Zhang D., Lu M., Zhang C., Sun Y. (2021). LncRNA SNHG14 Promotes Cell Proliferation and Invasion in Colorectal Cancer Through Modulating miR-519b-3p/DDX5 Axis. *Journal of Cancer*.

[61] Di W., Weinan X., Xin L. (2019). Long Noncoding RNA SNHG14 Facilitates Colorectal Cancer Metastasis Through Targeting EZH2-Regulated EPHA7. *Cell Death & Disease*.

[62] Lv T., Liu H., Wu Y., Huang W. (2021). Knockdown of lncRNA DLEU1 inhibits the Tumorigenesis of Oral Squamous Cell Carcinoma via Regulation of mIR-149-5p/CDK6 Axis. *Molecular Medicine Reports*.

[63] Christensen L. L., Tobiasen H., Holm A. (2013). MiRNA-362-3p Induces Cell Cycle Arrest Through Targeting of E2F1, USF2 and PTPN1 and is Associated With Recurrence of Colorectal Cancer. *International Journal of Cancer*.

[64] Li Y., Liu W., Liu C., Wang G., Zhou X. (2025). LncRNA SNHG25 Facilitates Colorectal Cancer Progression by Upregulating PPP2R2D Expression Through Sponging miR-329-3p. *Cytotechnology*.

[65] Hu Z., Li L., Cheng P. (2020). lncRNA MSC-AS1 Activates Wnt/*β*-Catenin Signaling Pathway to Modulate Cell Proliferation and Migration in Kidney Renal Clear Cell Carcinoma Via miR-3924/WNT5A. *Journal of Cellular Biochemistry*.

[66] Zali F., Ahmadi M., Ahmadyousefi M., Khodadadi Kohlan A., Alizadeh N., Soleimani M. (2025). Enhancing Chemotherapy for Colorectal Cancer: EGFR-Conjugated Ferritin Nanocages for Targeted Doxorubicin Delivery. *Journal of Drug Targeting*.

[67] Pan D. C., Krishnan V., Salinas A. K. (2021). Hyaluronic Acid-Doxorubicin Nanoparticles for Targeted Treatment of Colorectal Cancer. *Bioengineering & Translational Medicine*.

[68] Wilson P. M., Labonte M. J., Martin S. C. (2013). Sustained Inhibition of Deacetylases Is Required for the Antitumor Activity of the Histone Deactylase Inhibitors Panobinostat and Vorinostat in Models of Colorectal Cancer. *Investigational New Drugs*.

[69] Patra S., Praharaj P. P., Klionsky D. J., Bhutia S. K. (2022). Vorinostat in Autophagic Cell Death: A Critical Insight into Autophagy-Mediated, -Associated and -Dependent Cell Death for Cancer Prevention. *Drug Discovery Today*.

[70] Ghecham A., Senator A., Pawlowska E., Bouafia W., Błasiak J. (2019). Epigenetic Modifiers 5-Aza-2′-Deoxycytidine and Valproic Acid Differentially Change Viability, DNA Damage and Gene Expression in Metastatic and Non-Metastatic Colon Cancer Cell Lines. *Acta Biochimica Polonica*.

[71] Franco-Juárez E. X., González-Villasana V., Camacho-Moll M. E. (2024). Mechanistic Insights About Sorafenib-, Valproic Acid- and Metformin-Induced Cell Death in Hepatocellular Carcinoma. *International Journal of Molecular Sciences*.

[72] Sivakumar G. (2013). Colchicine Semisynthetics: Chemotherapeutics for Cancer?. *Current Medicinal Chemistry*.

[73] Meng J., Zhang H. H., Zhou C. X., Li C., Zhang F., Mei Q.-B. (2012). The Histone Deacetylase Inhibitor Trichostatin a Induces Cell Cycle Arrest and Apoptosis in Colorectal Cancer Cells Via P53-Dependent and -Independent Pathways. *Oncology Reports*.

[74] Dai L., He G., Zhang K., Guan X., Wang Y., Zhang B. (2018). Trichostatin a Induces P53-Dependent Endoplasmic Reticulum Stress in Human Colon Cancer Cells. *Oncology Letters*.

