# Supplementary material for: TMOD2 and DOCK4 as Novel Gut Microbiota-Associated Biomarkers for Colorectal Adenoma: Integrated Transcriptomic Analysis and Therapeutic Target Identification
Source: Mediators Inflamm. 2025 Dec 2;2025:6267309. doi: 10.1155/mi/6267309 (PMC12688639; doi:10.1155/mi/6267309)
Supplement: Supporting Information 3 — STROBE-MR checklist of recommended items to address in reports of Mendelian randomization studies. [file 6267309.f3.docx]

**STROBE-MR checklist of recommended items to address in reports of Mendelian randomization studies**^1^ ^2^

| **Item No.** | **Section** | **Checklist item** | **Page No.** | **Relevant text from manuscript** |
| --- | --- | --- | --- | --- |
| 1 | **TITLE and ABSTRACT** | Indicate Mendelian randomization (MR) as the study’s design in the title and/or the abstract if that is a main purpose of the study | 1 | Genetic evidence reveals causal role of gut microbiota in colorectal adenoma through novel transcriptomic biomarkers and therapeutic targets |
|  | **INTRODUCTION** |  |  |  |
| 2 | **Background** | Explain the scientific background and rationale for the reported study. What is the exposure? Is a potential causal relationship between exposure and outcome plausible? Justify why MR is a helpful method to address the study question | 2-3 | Colorectal cancer (CRC) remains the third most common malignancy worldwide, with colorectal adenomas (CRA) serving as its primary precancerous gateway. While affecting 20-30% of adults globally, these seemingly benign lesions harbor a troubling secret: 10-15% will inevitably progress to invasive carcinoma..... |
| 3 | **Objectives** | State specific objectives clearly, including pre-specified causal hypotheses (if any). State that MR is a method that, under specific assumptions, intends to estimate causal effects | 2-3 | Traditional observational studies cannot resolve this chicken-and-egg dilemma due to inherent confounding factors and reverse causality. Mendelian randomization (MR) offers an elegant solution by leveraging genetic variants as natural randomization tools, effectively mimicking randomized controlled trials while circumventing ethical constraints. |
|  | **METHODS** |  | 4-5 | We conducted two-sample MR analysis to investigate causal relationships between GM composition and CRA development. GM taxonomic features served as exposure variables, with CRA as the outcome..... |
| 4 | **Study design and data sources** | Present key elements of the study design early in the article. Consider including a table listing sources of data for all phases of the study. For each data source contributing to the analysis, describe the following: |  |  |
|  | a) | Setting: Describe the study design and the underlying population, if possible. Describe the setting, locations, and relevant dates, including periods of recruitment, exposure, follow-up, and data collection, when available. | 4-5 | GM genetic association data were obtained from the MiBioGen Consortium (https://mibiogen.gcc.rug.nl/), representing the largest available genome-wide association study (GWAS) of human gut microbiota composition... |
|  | b) | Participants: Give the eligibility criteria, and the sources and methods of selection of participants. Report the sample size, and whether any power or sample size calculations were carried out prior to the main analysis | 4-5 | This comprehensive dataset encompasses 18,340 individuals of predominantly European ancestry across 24 cohorts, ensuring robust statistical power for downstream analyses... |
|  | c) | Describe measurement, quality control and selection of genetic variants | 4 | This extensive dataset comprised 1,391 CRA cases and 461,542 controls, with genome-wide association signals across 9,851,867 SNP loci, providing sufficient statistical power for robust Mendelian randomization analysis..... |
|  | d) | For each exposure, outcome, and other relevant variables, describe methods of assessment and diagnostic criteria for diseases | 4 | CRA-associated genetic data (identifier: ukb-b-14210) were retrieved from the IEU OpenGWAS database (https://gwas.mrcieu.ac.uk/), derived from the UK Biobank |
|  | e) | Provide details of ethics committee approval and participant informed consent, if relevant |  | NA |
| 5 | **Assumptions** | Explicitly state the three core IV assumptions for the main analysis (relevance, independence and exclusion restriction) as well assumptions for any additional or sensitivity analysis | 5-6 | Our analysis adhered to three fundamental MR assumptions: (1) instrumental variables (IVs) demonstrate robust association with the exposure; (2) IVs influence the outcome exclusively through the exposure pathway (exclusion restriction); and (3) IVs remain independent of confounding factors affecting the exposure-outcome relationship... |
| 6 | **Statistical methods: main analysis** | Describe statistical methods and statistics used |  |  |
|  | a) | Describe how quantitative variables were handled in the analyses (i.e., scale, units, model) | 5 | Valid IVs were extracted from GM GWAS summary statistics using the extract_instruments function from the TwoSampleMR R package (version 0.6.4)... |
|  | b) | Describe how genetic variants were handled in the analyses and, if applicable, how their weights were selected | 5 | We implemented stringent screening criteria to ensure instrument validity: (1) genome-wide significant association with GM features (P < 1×10⁻⁵)9, 10; (2) independence ensured through linkage disequilibrium clumping (r² < 0.001, clumping window = 10 kb); (3) absence of direct association with CRA outcome (proxy variants allowed: rsq = 0.8); (4) instrument strength validation using F-statistics > 10, calculated as F = R²(N-K-1)/K(1-R²), where R² represents cumulative explained variance, N denotes sample size, and K indicates SNP count; (5) exclusion of palindromic SNPs and GM features with fewer than 3 valid instruments; and (6) minor allele frequency threshold > 0.01 to ensure adequate statistical power..... |
|  | c) | Describe the MR estimator (e.g. two-stage least squares, Wald ratio) and related statistics. Detail the included covariates and, in case of two-sample MR, whether the same covariate set was used for adjustment in the two samples | 5 | Effect alleles and effect sizes were harmonized across exposure and outcome datasets using the harmonise_data function. .... |
|  | d) | Explain how missing data were addressed |  | na |
|  | e) | If applicable, indicate how multiple testing was addressed |  | NA |
| 7 | **Assessment of assumptions** | Describe any methods or prior knowledge used to assess the assumptions or justify their validity | 3-4 | Traditional observational studies cannot resolve this chicken-and-egg dilemma due to inherent confounding factors and reverse causality. Mendelian randomization (MR) offers an elegant solution by leveraging genetic variants as natural randomization tools, effectively mimicking randomized controlled trials while circumventing ethical constraints..... |
| 8 | **Sensitivity analyses and additional analyses** | Describe any sensitivity analyses or additional analyses performed (e.g. comparison of effect estimates from different approaches, independent replication, bias analytic techniques, validation of instruments, simulations) | 5-6 | Comprehensive sensitivity analyses were performed to validate result robustness: (1) Cochran's Q heterogeneity test (mr_heterogeneity, P > 0.05 indicating homogeneity); (2) MR-Egger intercept test for horizontal pleiotropy (mr_pleiotropy_test, P > 0.05 indicating absence of pleiotropy)... |
| 9 | **Software and pre-registration** |  |  |  |
|  | a) | Name statistical software and package(s), including version and settings used | 5 | the TwoSampleMR R package (version 0.6.4).. |
|  | b) | State whether the study protocol and details were pre-registered (as well as when and where) |  | NA |
|  | **RESULTS** |  |  |  |
| 10 | **Descriptive data** |  |  |  |
|  | a) | Report the numbers of individuals at each stage of included studies and reasons for exclusion. Consider use of a flow diagram | 3-4 | GM genetic association data were obtained from the MiBioGen Consortium (https://mibiogen.gcc.rug.nl/), representing the largest available genome-wide association study (GWAS) of human gut microbiota composition. This comprehensive dataset encompasses 18,340 individuals of predominantly European ancestry across 24 cohorts.. |
|  | b) | Report summary statistics for phenotypic exposure(s), outcome(s), and other relevant variables (e.g. means, SDs, proportions) | 14 | The symmetric and uniform distribution of SNPs across all taxa supported adherence to Mendel's second law of independent assortment. |
|  | c) | If the data sources include meta-analyses of previous studies, provide the assessments of heterogeneity across these studies |  | NA |
|  | d) | For two-sample MR:  i.  Provide justification of the similarity of the genetic variant-exposure associations between the exposure and outcome samples  ii.  Provide information on the number of individuals who overlap between the exposure and outcome studies |  | NA |
| 11 | **Main results** |  |  |  |
|  | a) | Report the associations between genetic variant and exposure, and between genetic variant and outcome, preferably on an interpretable scale | 14 | Using the IVW method, we identified 12 gut microbiota taxa demonstrating significant causal associations with CRA risk (P < 0.05) (Figure 1, Table S1)... |
|  | b) | Report MR estimates of the relationship between exposure and outcome, and the measures of uncertainty from the MR analysis, on an interpretable scale, such as odds ratio or relative risk per SD difference | 14 | Among these, 8 taxa were identified as risk factors (odds ratio [OR] > 1.0, 95% confidence interval [CI] excluding 1.0), while 4 taxa exhibited protective effects (OR < 1.0, 95% CI excluding 1.0). Scatter plot analysis revealed that positive slopes corresponded to risk-associated taxa, whereas negative slopes indicated protective taxa, with intercepts approximating zero, suggesting minimal confounding bias... |
|  | c) | If relevant, consider translating estimates of relative risk into absolute risk for a meaningful time period |  | NA |
|  | d) | Consider plots to visualize results (e.g. forest plot, scatterplot of associations between genetic variants and outcome versus between genetic variants and exposure) | 14 | Using the IVW method, we identified 12 gut microbiota taxa demonstrating significant causal associations with CRA risk (P < 0.05) (Figure 1, Table S1). |
| 12 | **Assessment of assumptions** |  |  |  |
|  | a) | Report the assessment of the validity of the assumptions | 14 | Comprehensive sensitivity analyses confirmed the robustness of our findings. Fixed-effect inverse-variance weighted analysis yielded non-significant results (P > 0.05) for all 12 taxa, indicating absence of directional pleiotropy (Table S2)... |
|  | b) | Report any additional statistics (e.g., assessments of heterogeneity across genetic variants, such as *I^2^*, Q statistic or E-value) | 14 | MR-Egger regression intercept tests demonstrated no horizontal pleiotropy across all associations (P > 0.05) (Table S3). Leave-one-out analysis confirmed that no individual SNP disproportionately influenced the causal estimates, supporting result stability (Supplementary Figure S4). |
| 13 | **Sensitivity analyses and additional analyses** |  |  |  |
|  | a) | Report any sensitivity analyses to assess the robustness of the main results to violations of the assumptions | 14 | sensitivity analyses confirmed the robustness of our findings. Fixed-effect inverse-variance weighted analysis yielded non-significant results (P > 0.05) for all 12 taxa, indicating absence of directional pleiotropy... |
|  | b) | Report results from other sensitivity analyses or additional analyses | 14 | Leave-one-out analysis confirmed that no individual SNP disproportionately influenced the causal estimates, supporting result stability.. |
|  | c) | Report any assessment of direction of causal relationship (e.g., bidirectional MR) | 14 | Steiger directionality tests validated the correct causal direction for all associations (P < 0.01, correct causal direction = TRUE), excluding reverse causality bias |
|  | d) | When relevant, report and compare with estimates from non-MR analyses |  | NA |
|  | e) | Consider additional plots to visualize results (e.g., leave-one-out analyses) | 14 | Leave-one-out analysis confirmed that no individual SNP disproportionately influenced the causal estimates, supporting result stability (Supplementary Figure S4). |
|  | **DISCUSSION** |  | 22-25 | Colorectal adenomas represent precancerous lesions that constitute critical intervention points in preventing colorectal cancer progression, with emerging evidence highlighting the pivotal role of gut microbiota in adenoma pathogenesis.. |
| 14 | **Key results** | Summarize key results with reference to study objectives | 25 | Through integrative bioinformatics analysis, we identified TMOD2 and DOCK4 as novel diagnostic biomarkers for colorectal adenomas, achieving 88% diagnostic accuracy while revealing their fundamental roles in adenoma pathogenesis through cytoskeletal regulation, cell cycle control, and immune microenvironment modulation. . ... |
| 15 | **Limitations** | Discuss limitations of the study, taking into account the validity of the IV assumptions, other sources of potential bias, and imprecision. Discuss both direction and magnitude of any potential bias and any efforts to address them | 24-25 | several limitations warrant consideration. The analysis relies heavily on computational predictions and public datasets, which may not fully capture the complexity of individual patient variations or tissue-specific expression patterns. .. |
| 16 | **Interpretation** |  |  |  |
|  | a) | Meaning: Give a cautious overall interpretation of results in the context of their limitations and in comparison with other studies | 25 | Despite these limitations, our findings establish TMOD2 and DOCK4 as novel biomarkers with significant diagnostic and therapeutic potential in colorectal adenomas.. |
|  | b) | Mechanism: Discuss underlying biological mechanisms that could drive a potential causal relationship between the investigated exposure and the outcome, and whether the gene-environment equivalence assumption is reasonable. Use causal language carefully, clarifying that IV estimates may provide causal effects only under certain assumptions | 22-25 | The involvement in immune microenvironment regulation and genomic stability maintenance31 further underscores TMOD2's multifaceted role in adenoma pathogenesis... |
|  | c) | Clinical relevance: Discuss whether the results have clinical or public policy relevance, and to what extent they inform effect sizes of possible interventions | 25 | Despite these limitations, our findings establish TMOD2 and DOCK4 as novel biomarkers with significant diagnostic and therapeutic potential in colorectal adenomas. The comprehensive molecular characterization provides a foundation for targeted intervention strategies and personalized risk assessment, ultimately contributing to improved patient outcomes through early detection and precision medicine approaches.,. |
| 17 | **Generalizability** | Discuss the generalizability of the study results (a) to other populations, (b) across other exposure periods/timings, and (c) across other levels of exposure | 15 | our findings establish TMOD2 and DOCK4 as novel biomarkers with significant diagnostic and therapeutic potential in colorectal adenomas.. |
|  | **OTHER INFORMATION** |  |  |  |
| 18 | **Funding** | Describe sources of funding and the role of funders in the present study and, if applicable, sources of funding for the databases and original study or studies on which the present study is based | 26 | This study was funded by the... |
| 19 | **Data and data sharing** | Provide the data used to perform all analyses or report where and how the data can be accessed, and reference these sources in the article. Provide the statistical code needed to reproduce the results in the article, or report whether the code is publicly accessible and if so, where | 27 | All data utilized in this study are publicly available through established repositories.... |
| 20 | **Conflicts of Interest** | All authors should declare all potential conflicts of interest | 27 | The authors declare no conflicts of interest.. |

This checklist is copyrighted by the Equator Network under the Creative Commons Attribution 3.0 Unported (CC BY 3.0) license.

1. Skrivankova VW, Richmond RC, Woolf BAR, Yarmolinsky J, Davies NM, Swanson SA, et al. Strengthening the Reporting of Observational Studies in Epidemiology using Mendelian Randomization (STROBE-MR) Statement. JAMA. 2021;under review.

2. Skrivankova VW, Richmond RC, Woolf BAR, Davies NM, Swanson SA, VanderWeele TJ, et al. Strengthening the Reporting of Observational Studies in Epidemiology using Mendelian Randomisation (STROBE-MR): Explanation and Elaboration. BMJ. 2021;375:n2233.
