# Supplementary material for: TMOD2 and DOCK4 as Novel Gut Microbiota-Associated Biomarkers for Colorectal Adenoma: Integrated Transcriptomic Analysis and Therapeutic Target Identification
Source: Mediators Inflamm. 2025 Dec 2;2025:6267309. doi: 10.1155/mi/6267309 (PMC12688639; doi:10.1155/mi/6267309)
Supplement: Supporting Information 2 — Figure S1. SNP-exposure and SNP-outcome scatter plots for gut microbiota-CRA Mendelian randomization analysis. Figure S2. Effect size distribution plots for protective and risk-associated gut microbiota taxa. Figure S3. SNP distribution plots confirming Mendel's second law adherence. Figure S4. Leave-one-out sensitivity analysis plots for causal estimate stability. Figure S5. DNA-binding motif analysis and base stacking patterns for TMOD2 and DOCK4 regulatory regions. [file 6267309.f2.pdf]

Supplementary Figure 1

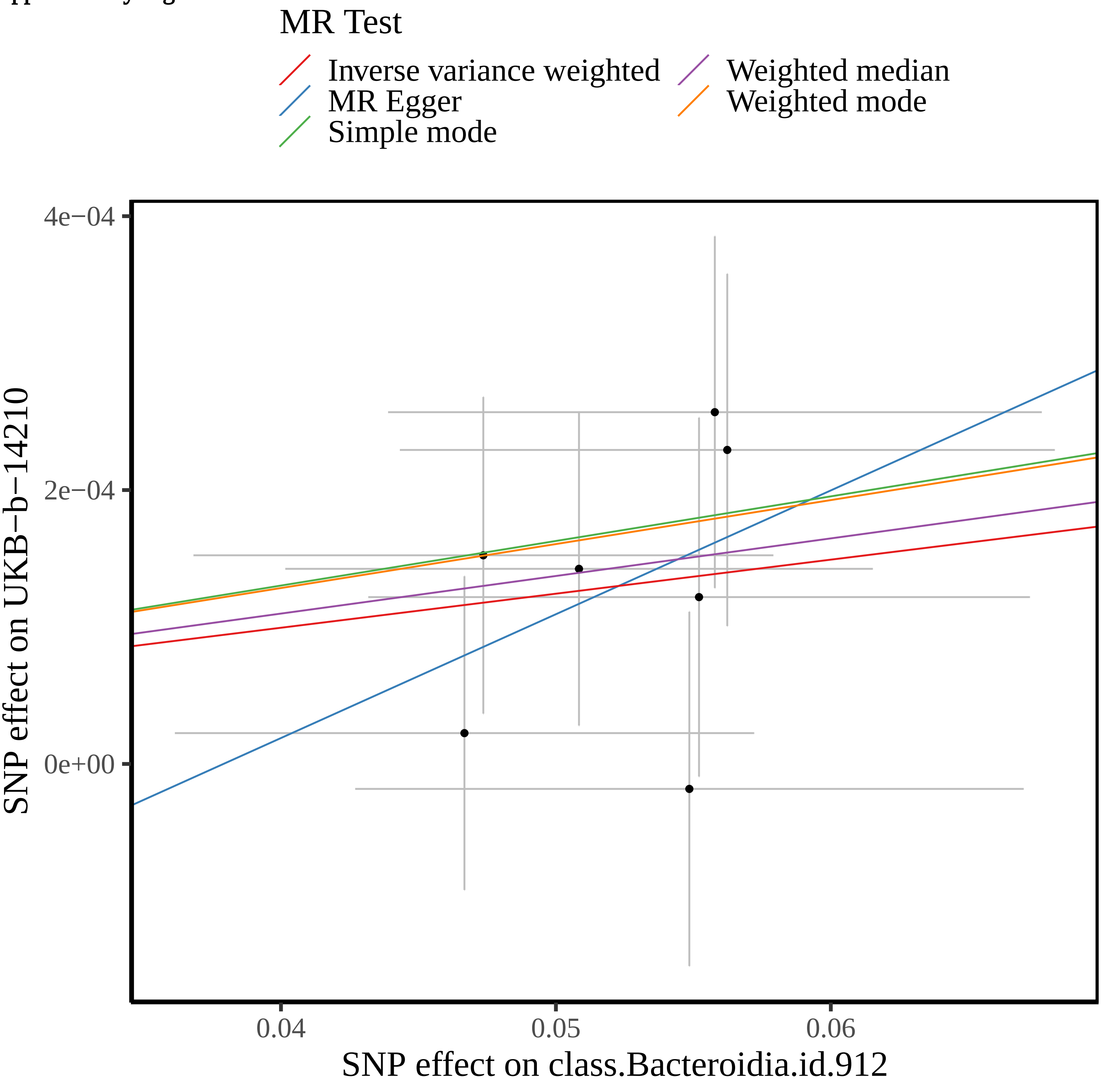

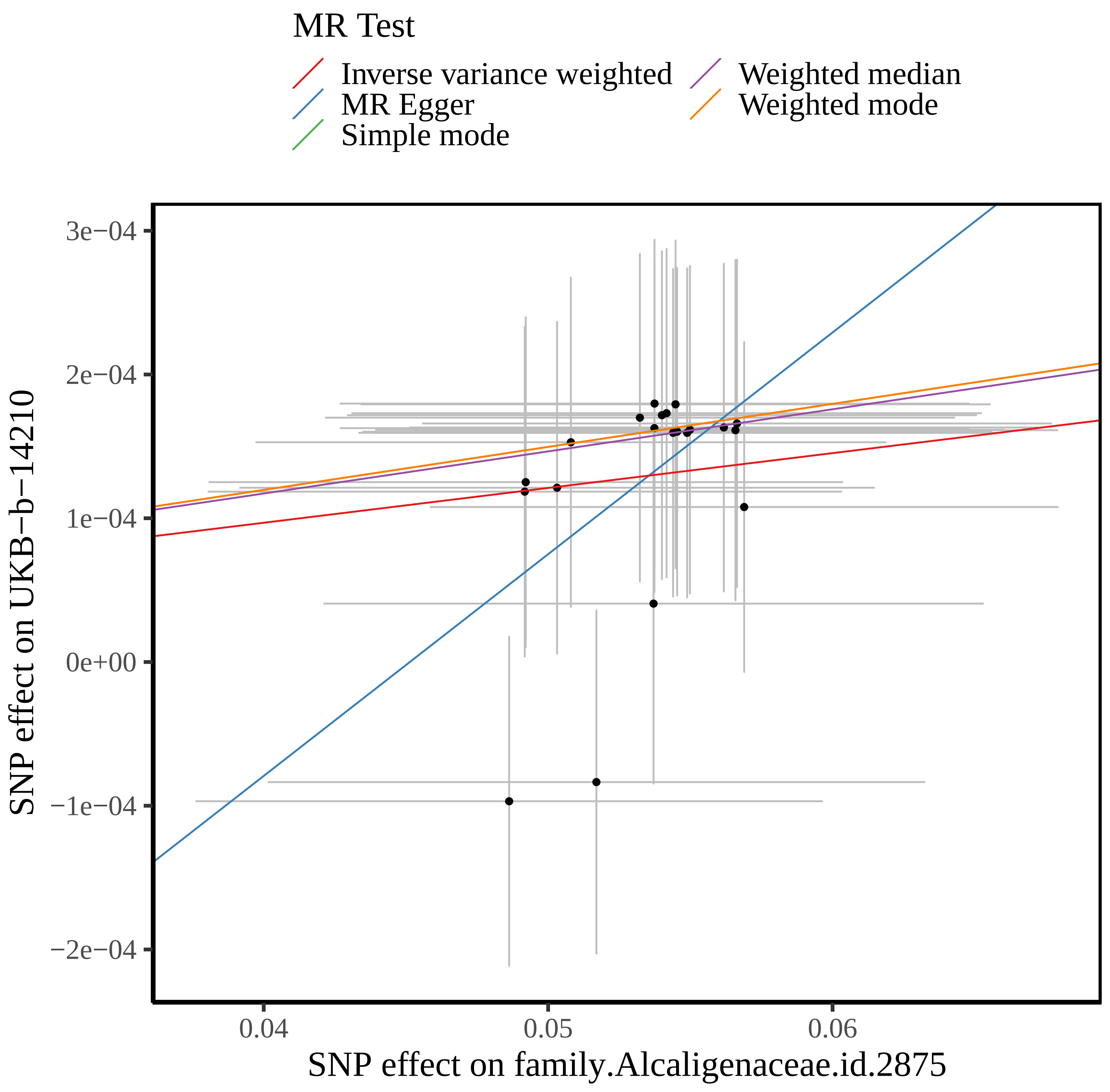

MR Test
Inverse variance weighted
MR Egger
Simple mode
Weighted median
Weighted mode
3e−04
2e−04
1e−04
0e+00
−1e−04
−2e−04
SNP effect on UKB−b−14210
0.04
0.05
0.06
SNP effect on family.Alcaligenaceae.id.2875

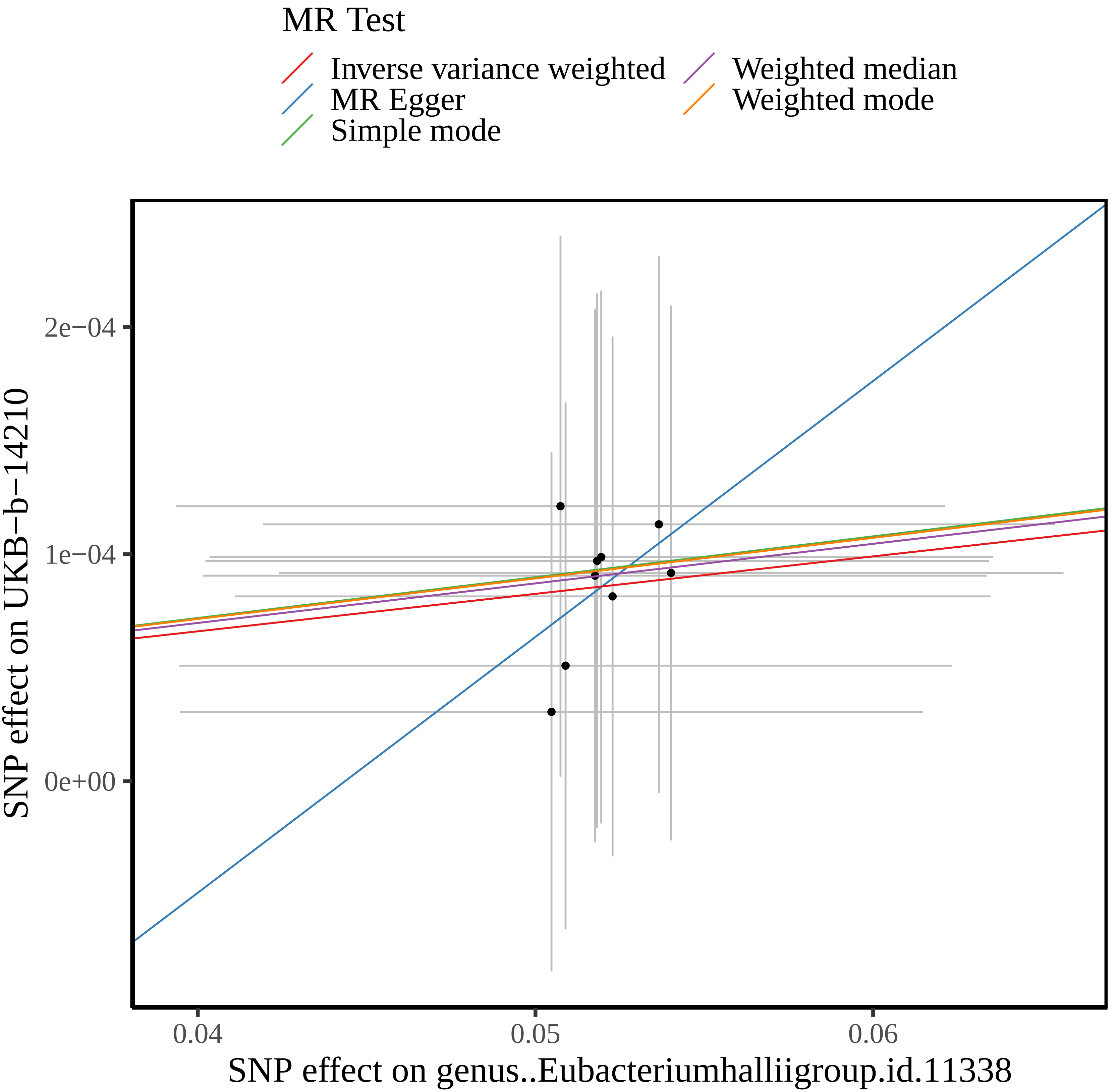

MR Test
Inverse variance weighted
MR Egger
Simple mode
Weighted median
Weighted mode
SNP effect on UKB−b−14210
2e−04
1e−04
0e+00
0.04
0.05
0.06
SNP effect on genus..Eubacteriumhalliigroup.id.11338

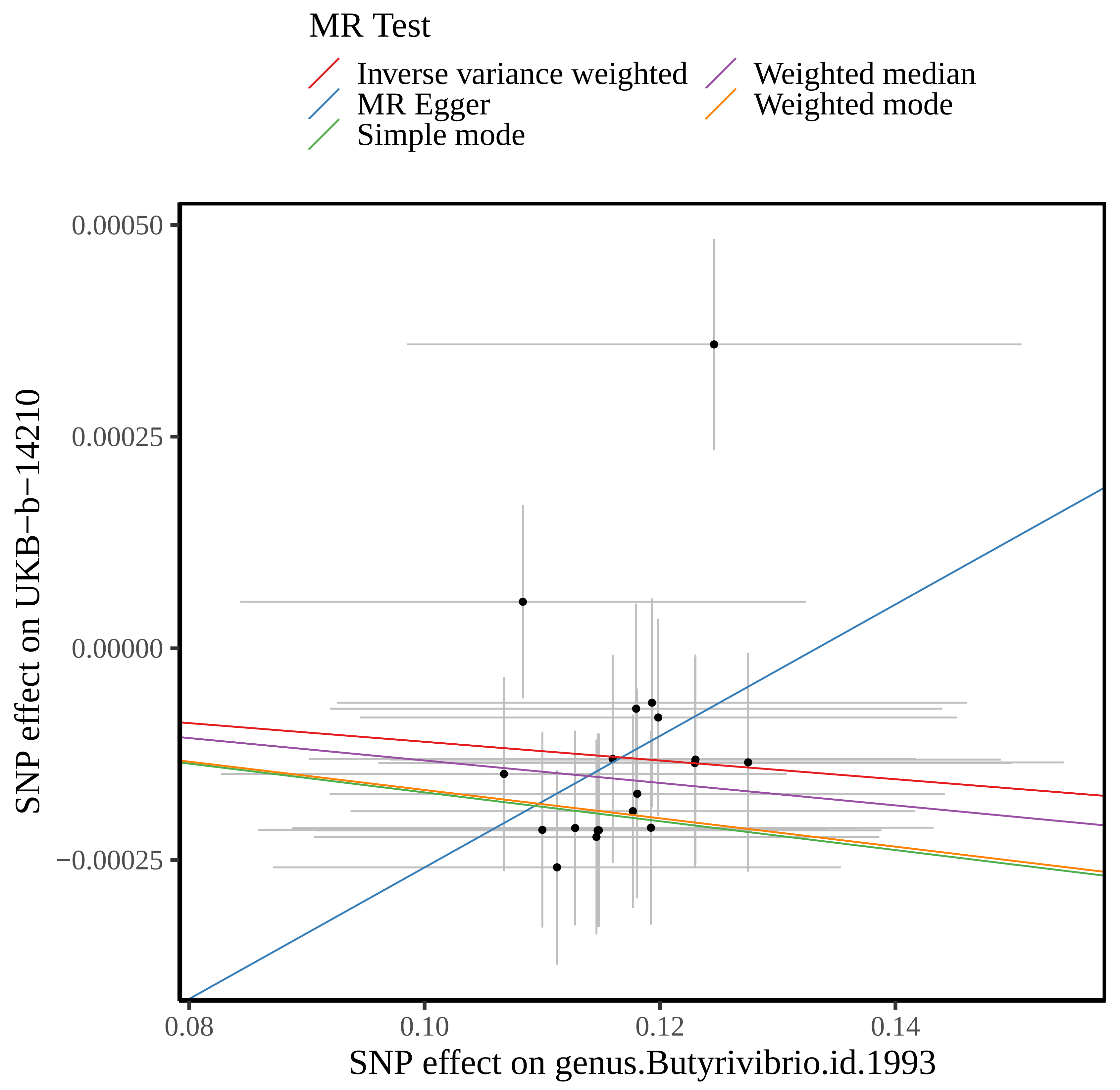
MR Test
Inverse variance weighted
MR Egger
Simple mode
Weighted median
Weighted mode
0.00050
0.00025
0.00000
−0.00025
0.08
0.10
0.12
0.14
SNP effect on UKB−b−14210
SNP effect on genus.Butyrivibrio.id.1993

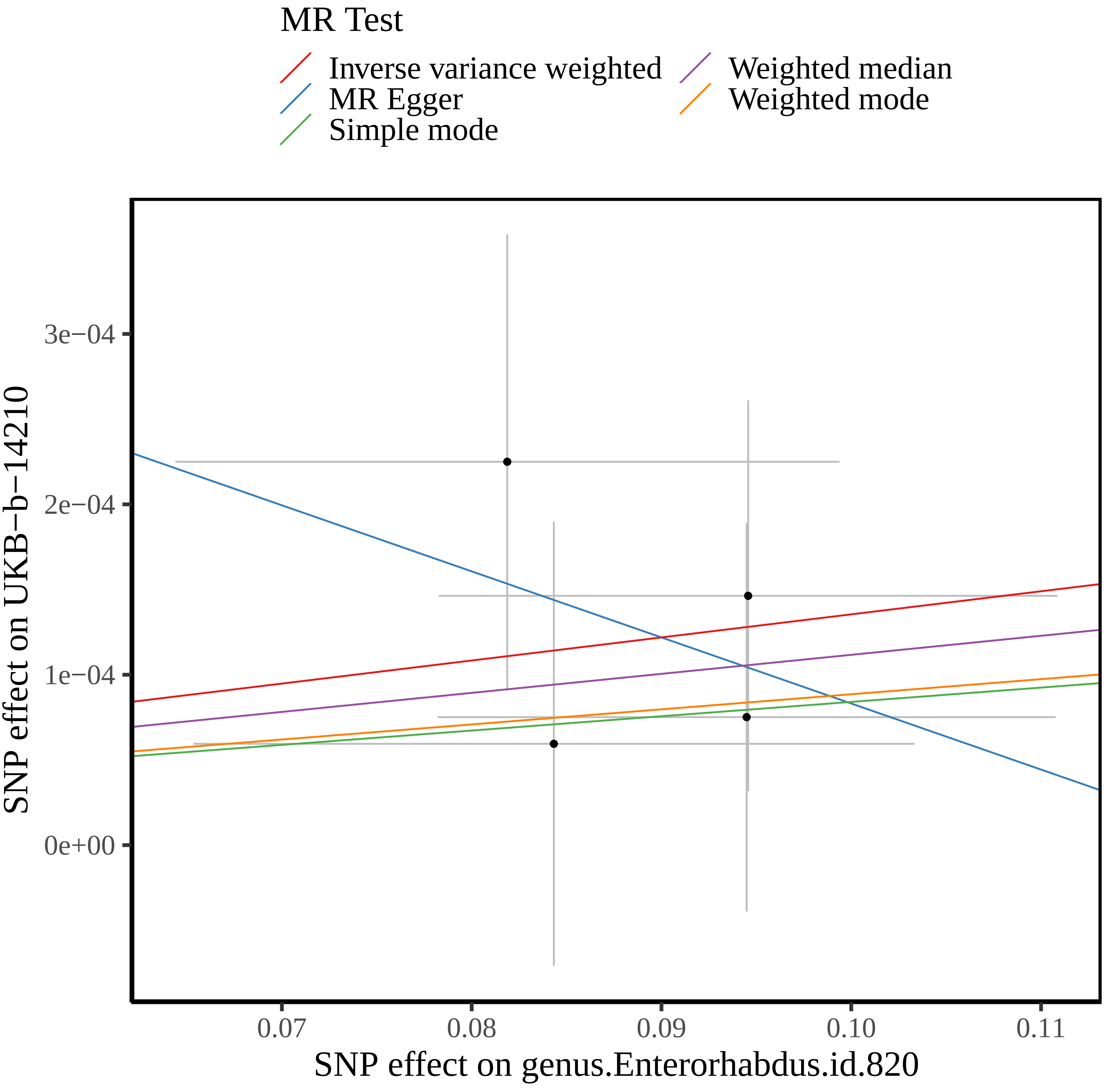
MR Test
Inverse variance weighted
MR Egger
Simple mode
Weighted median
Weighted mode
3e−04
2e−04
1e−04
0e+00
SNP effect on UKB−b−14210
0.07
0.08
0.09
0.10
0.11
SNP effect on genus.Enterorhabdus.id.820

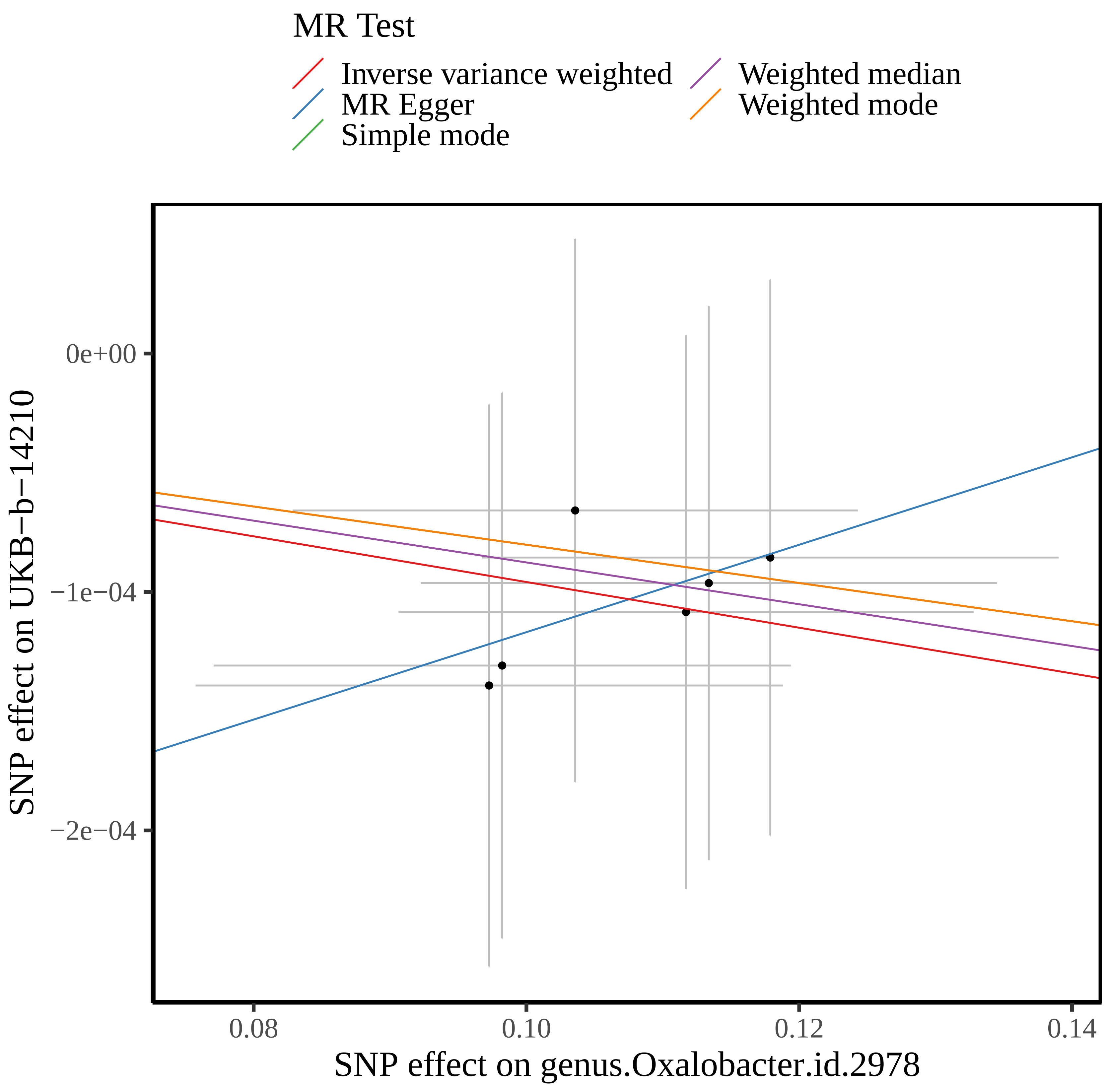
MR Test
Inverse variance weighted
MR Egger
Simple mode
Weighted median
Weighted mode
0e+00
−1e−04
−2e−04
SNP effect on UKB−b−14210
0.08
0.10
0.12
0.14
SNP effect on genus.Oxalobacter.id.2978

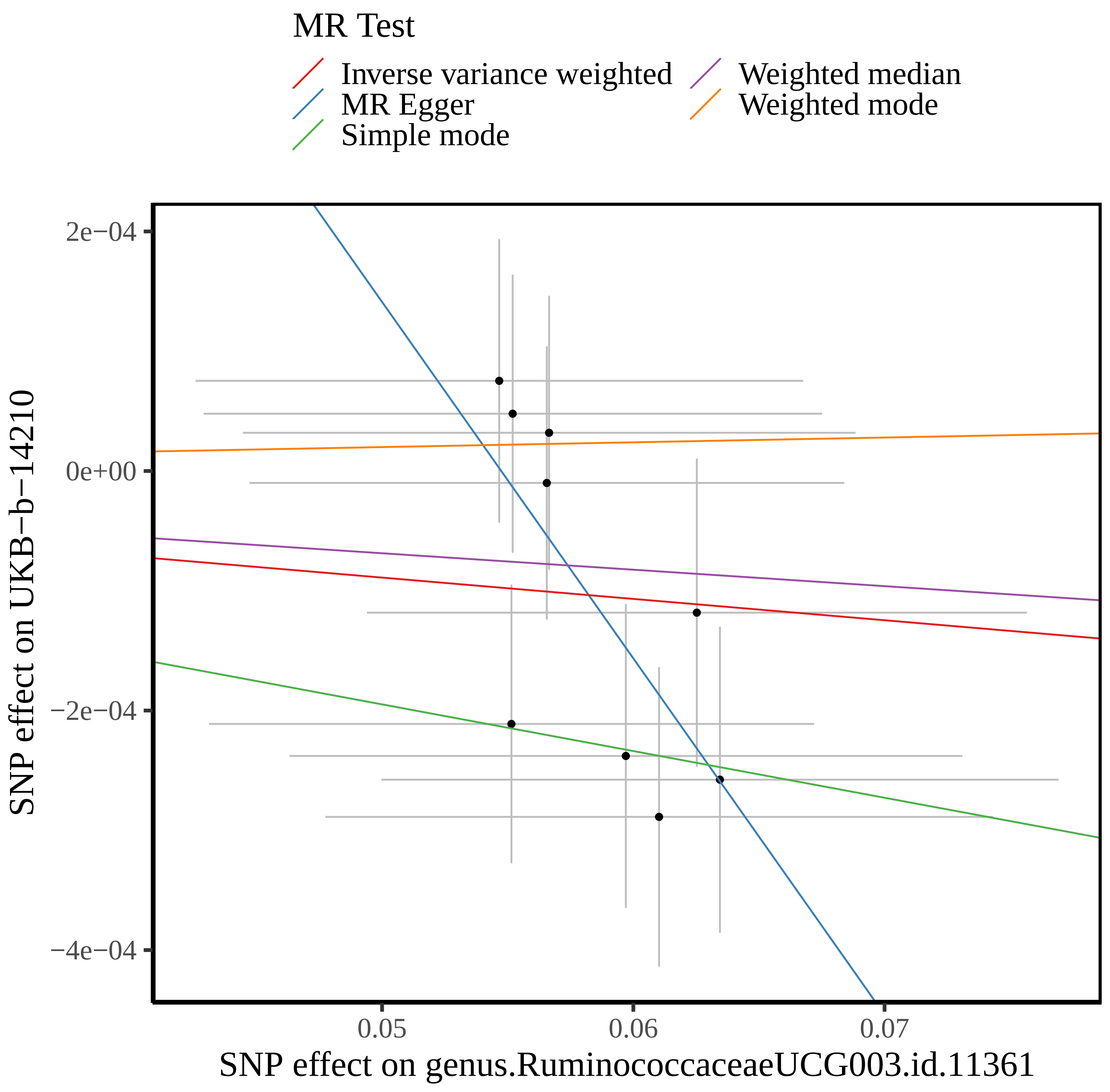

MR Test
Inverse variance weighted
MR Egger
Simple mode
Weighted median
Weighted mode
2e−04
0e+00
−2e−04
−4e−04
0.05
0.06
0.07
SNP effect on UKB−b−14210
SNP effect on genus.RuminococcaceaeUCG003.id.11361

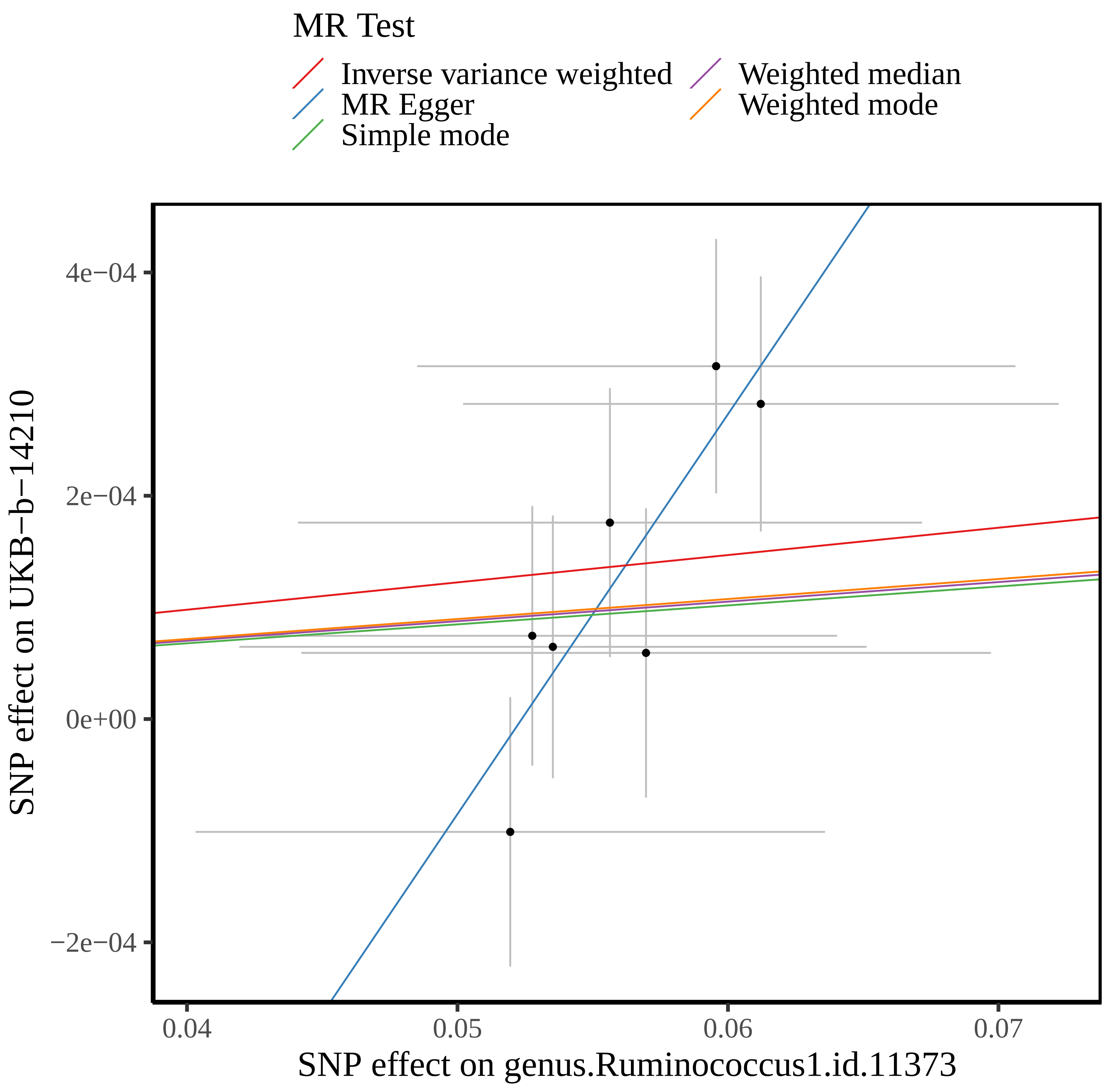
MR Test
Inverse variance weighted
MR Egger
Simple mode
Weighted median
Weighted mode
4e−04
2e−04
0e+00
−2e−04
0.04
0.05
0.06
0.07
SNP effect on UKB−b−14210
SNP effect on genus.Ruminococcus1.id.11373

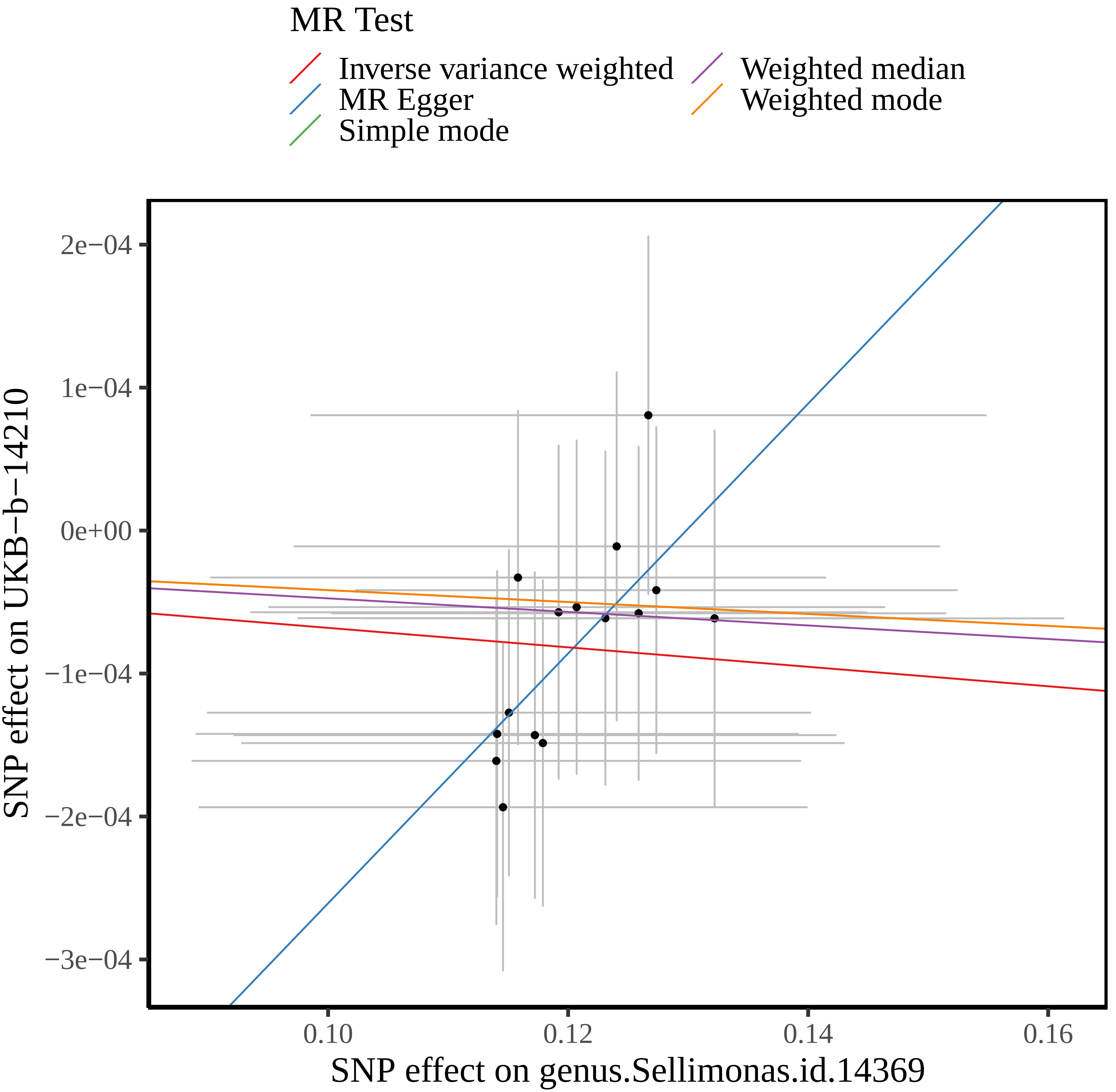

MR Test
Inverse variance weighted
MR Egger
Simple mode
Weighted median
Weighted mode
2e−04
1e−04
0e+00
−1e−04
−2e−04
−3e−04
0.10
0.12
0.14
0.16
SNP effect on genus.Sellimonas.id.14369
SNP effect on UKB−b−14210

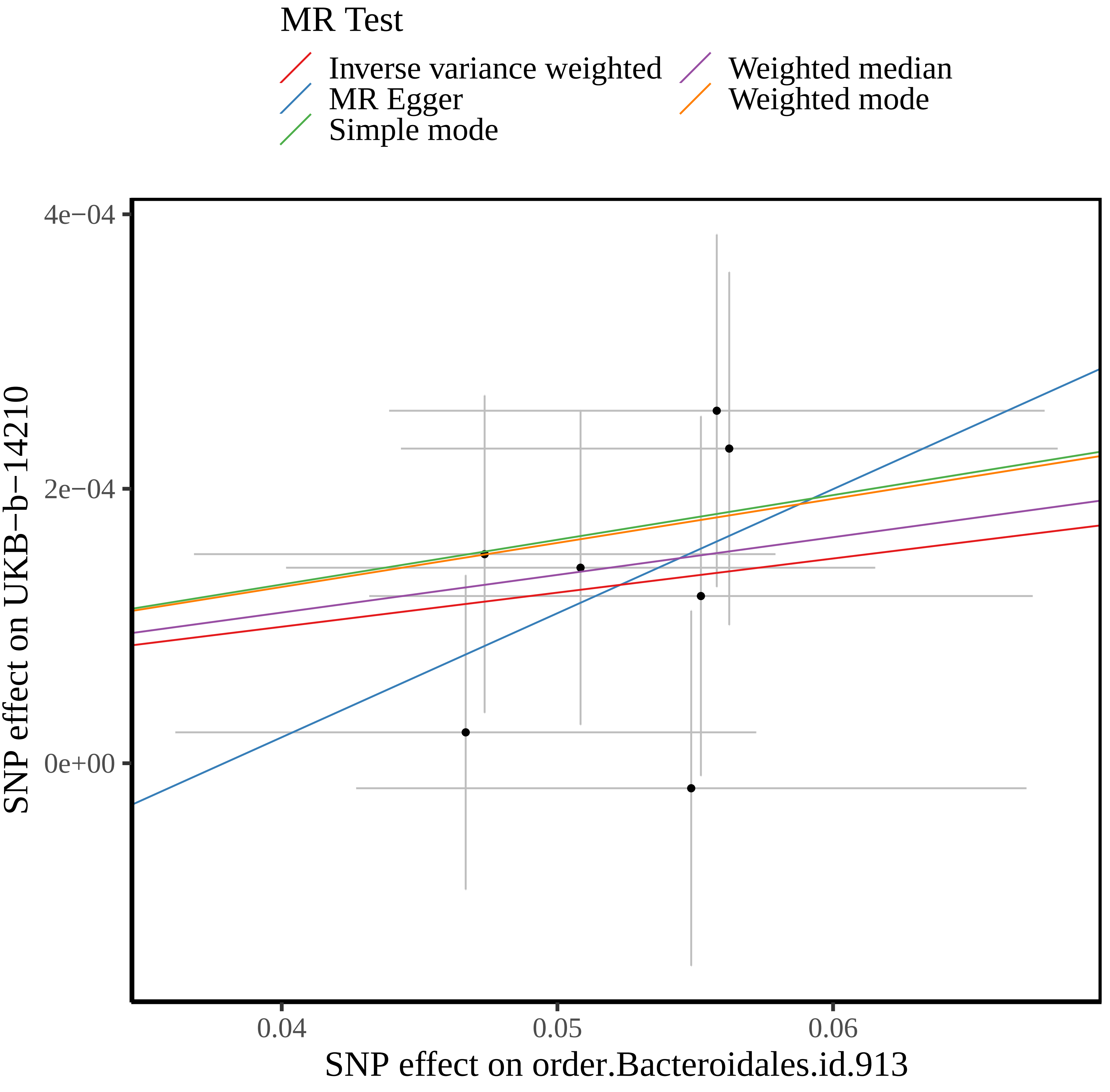

MR Test
Inverse variance weighted
MR Egger
Simple mode
Weighted median
Weighted mode
4e−04
2e−04
0e+00
SNP effect on UKB−b−14210
0.04
0.05
0.06
SNP effect on order.Bacteroidales.id.913

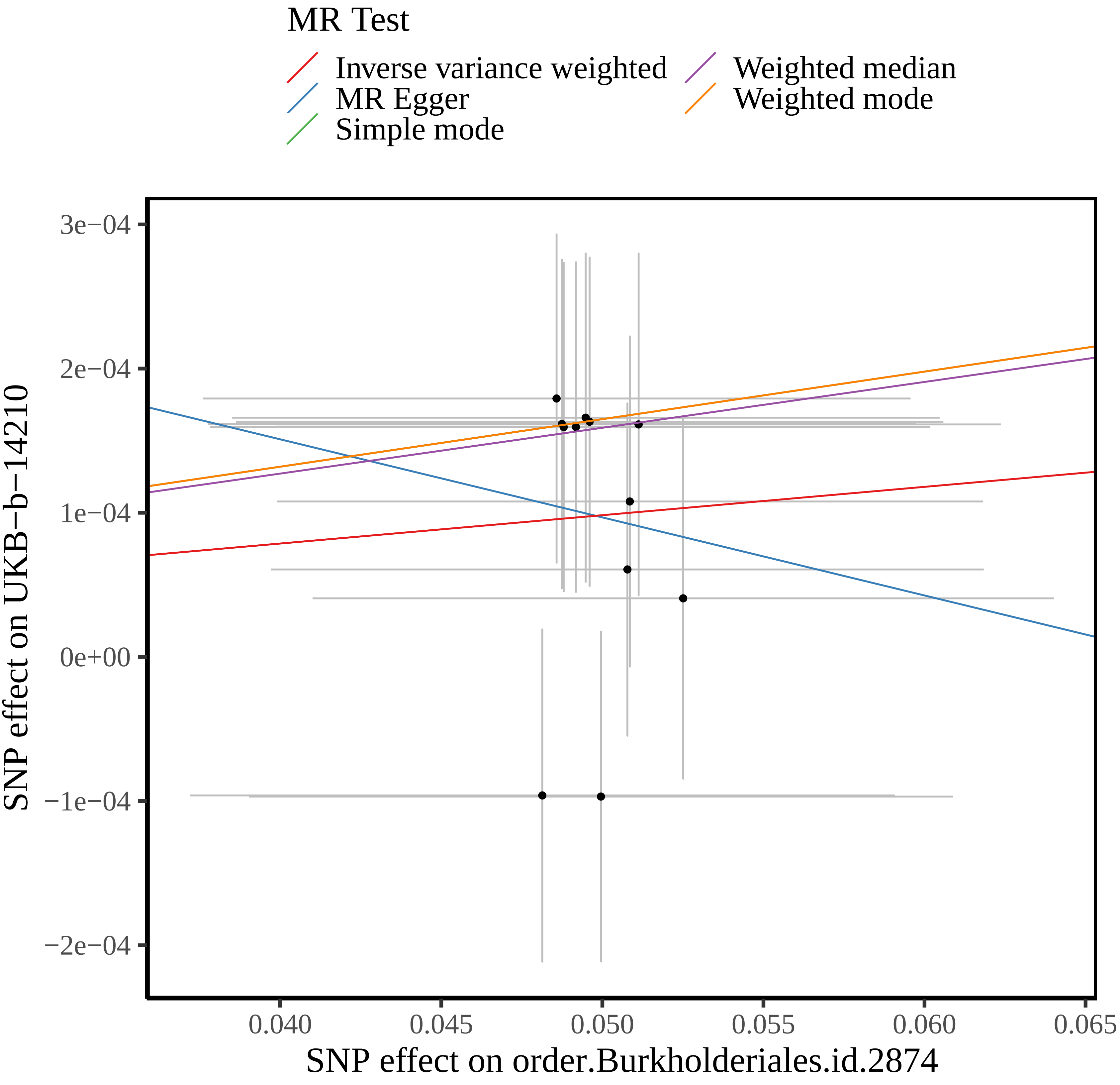
MR Test
Inverse variance weighted
MR Egger
Simple mode
Weighted median
Weighted mode
3e−04
2e−04
1e−04
0e+00
−1e−04
−2e−04
SNP effect on UKB−b−14210
0.040
0.045
0.050
0.055
0.060
0.065
SNP effect on order.Burkholderiales.id.2874

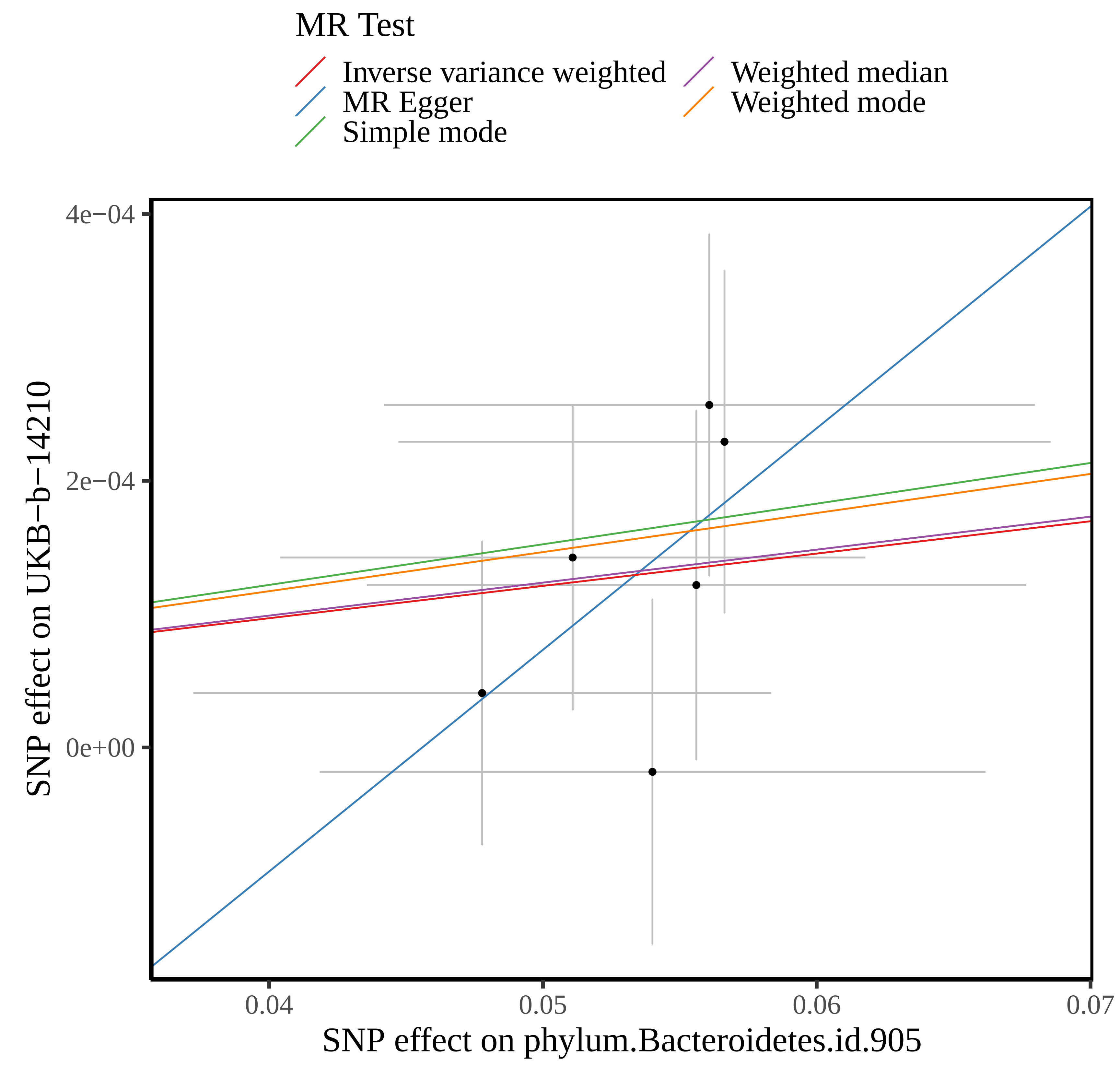

MR Test
Inverse variance weighted
MR Egger
Simple mode
Weighted median
Weighted mode
4e−04
2e−04
0e+00
SNP effect on UKB−b−14210
0.04
0.05
0.06
0.07
SNP effect on phylum.Bacteroidetes.id.905

Supplementary Figure 2

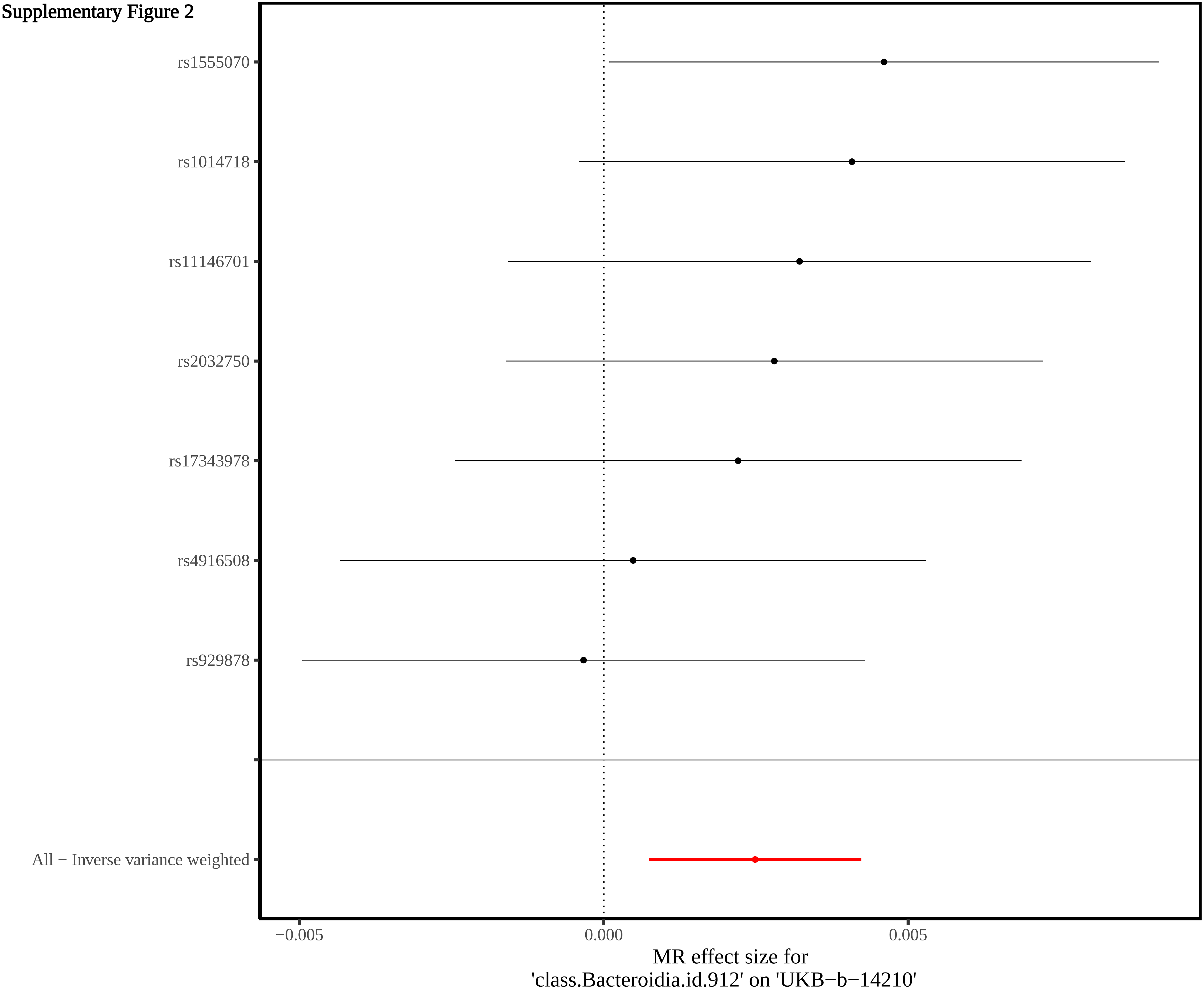

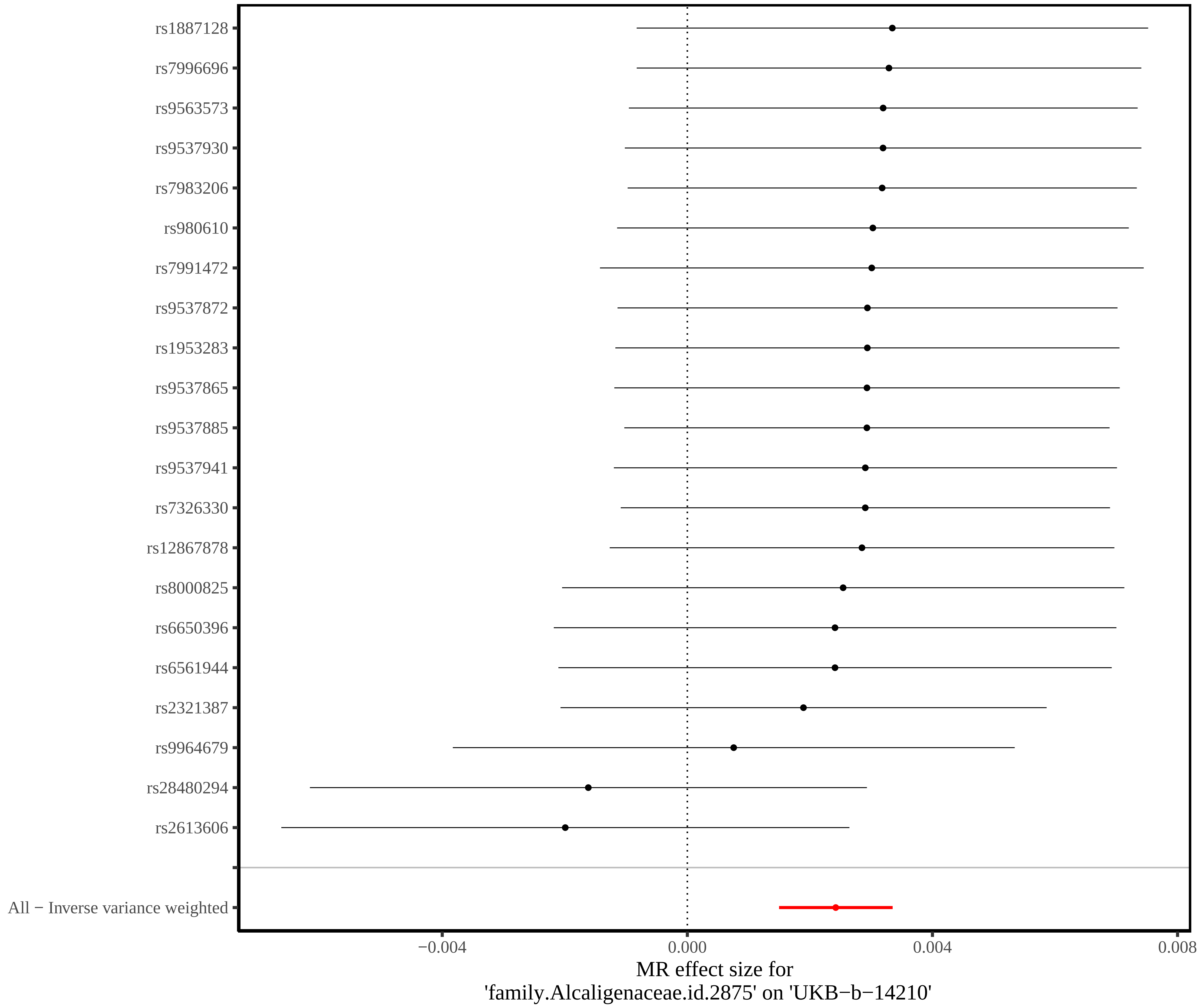
rs1887128
rs7996696
rs9563573
rs9537930
rs7983206
rs980610
rs7991472
rs9537872
rs1953283
rs9537865
rs9537885
rs9537941
rs7326330
rs12867878
rs8000825
rs6650396
rs6561944
rs2321387
rs9964679
rs28480294
rs2613606
All − Inverse variance weighted
−0.004
0.000
0.004
0.008
MR effect size for
'family.Alcaligenaceae.id.2875' on 'UKB−b−14210'

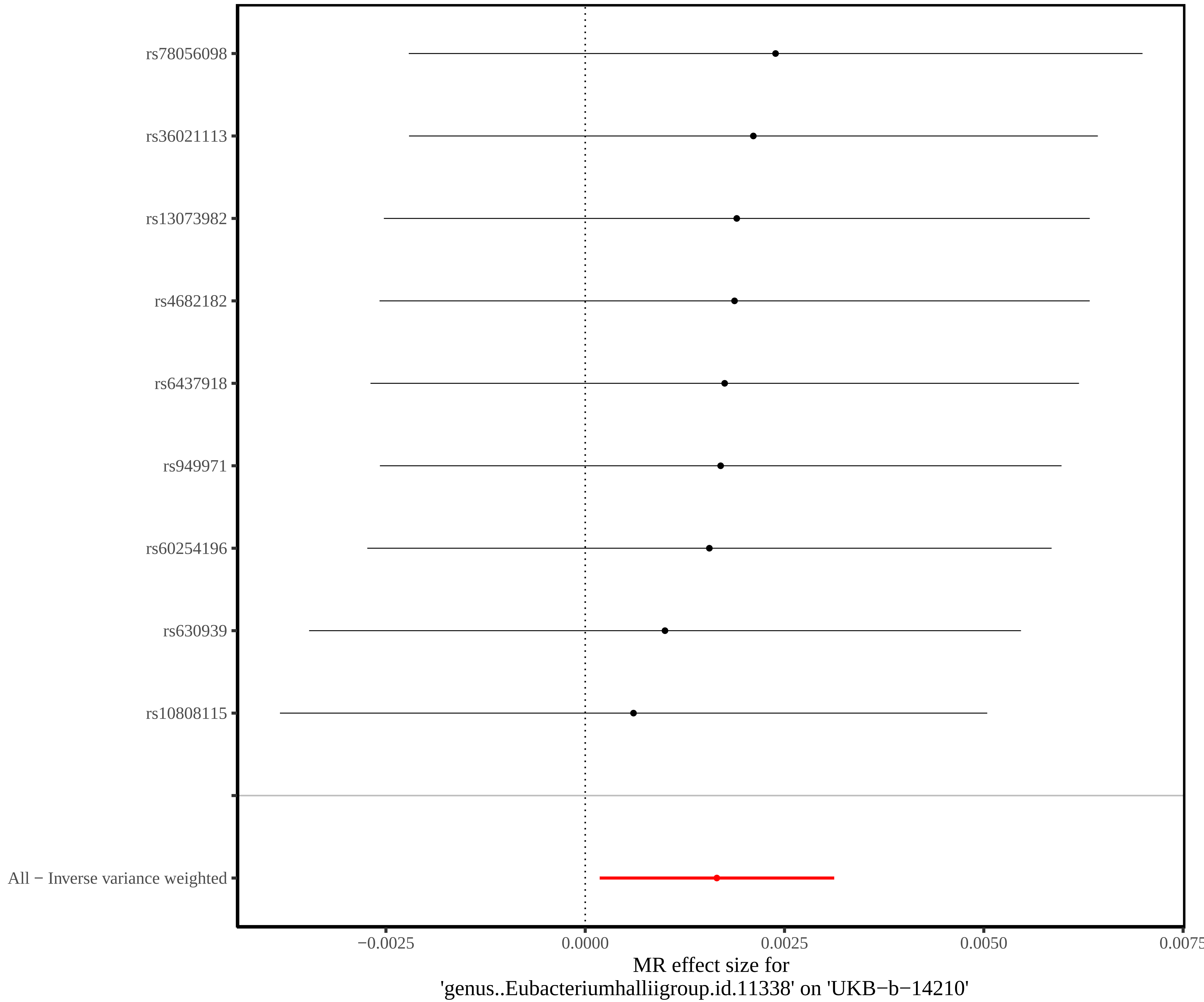

rs78056098
rs36021113
rs13073982
rs4682182
rs6437918
rs949971
rs60254196
rs630939
rs10808115
All − Inverse variance weighted
−0.0025
0.0000
0.0025
0.0050
MR effect size for
'genus..Eubacteriumhalliigroup.id.11338' on 'UKB−b−14210'

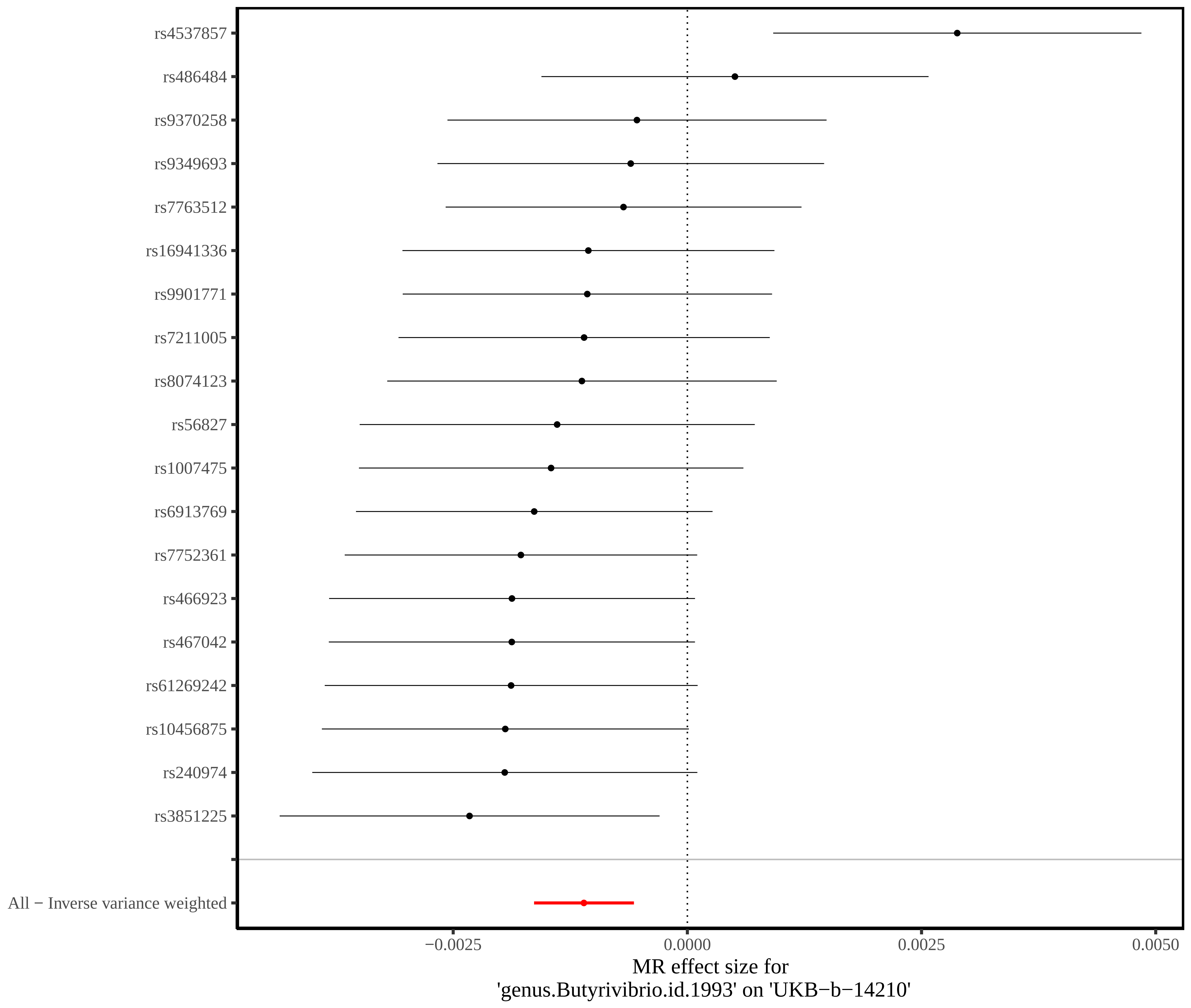
rs4537857
rs486484
rs9370258
rs9349693
rs7763512
rs16941336
rs9901771
rs7211005
rs8074123
rs56827
rs1007475
rs6913769
rs7752361
rs466923
rs467042
rs61269242
rs10456875
rs240974
rs3851225
All − Inverse variance weighted
−0.0025
0.0000
0.0025
0.0050
MR effect size for
'genus.Butyrivibrio.id.1993' on 'UKB−b−14210'

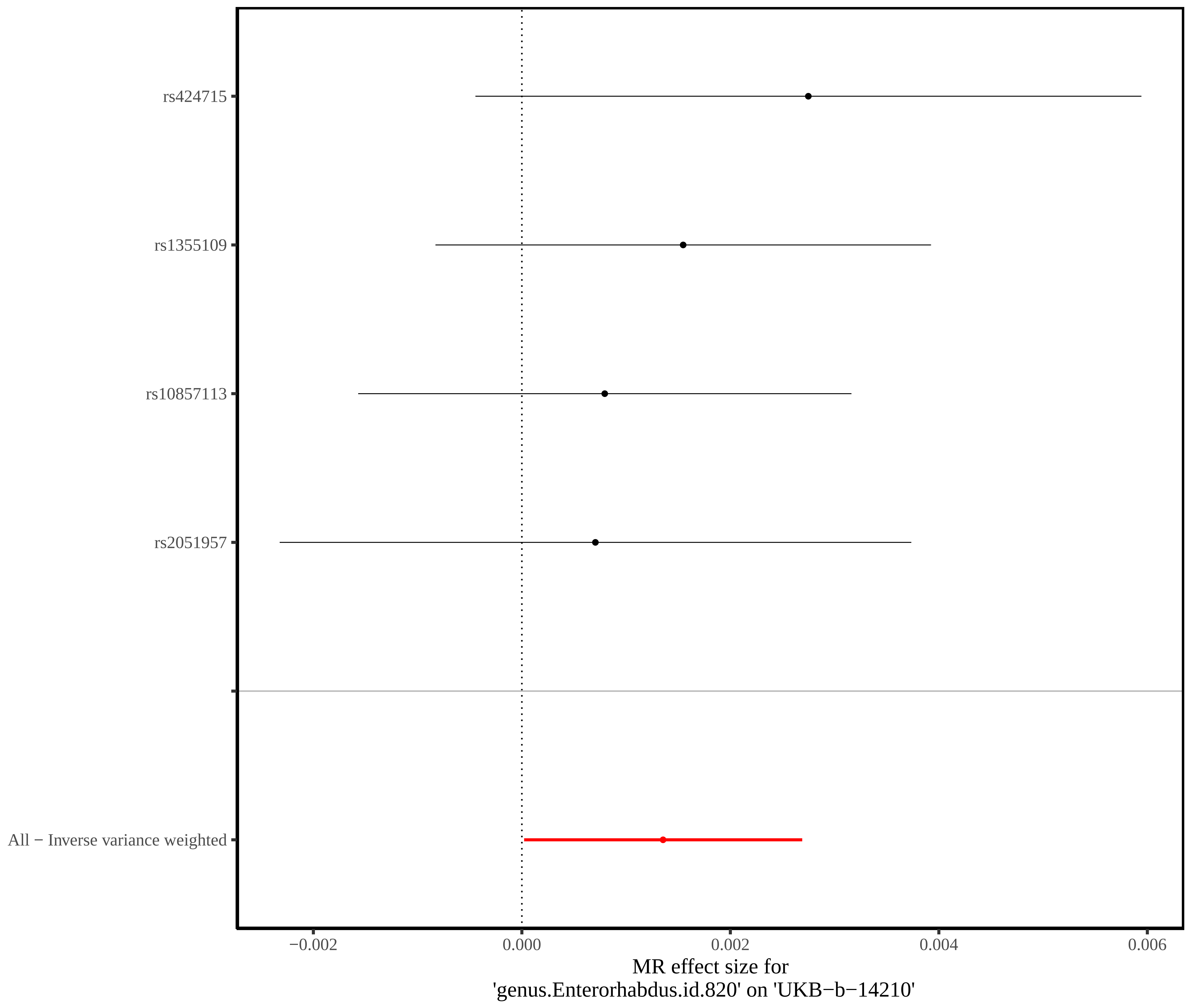

rs424715
rs1355109
rs10857113
rs2051957
All − Inverse variance weighted
−0.002
0.000
0.002
0.004
0.006
MR effect size for
'genus.Enterorhabdus.id.820' on 'UKB−b−14210'

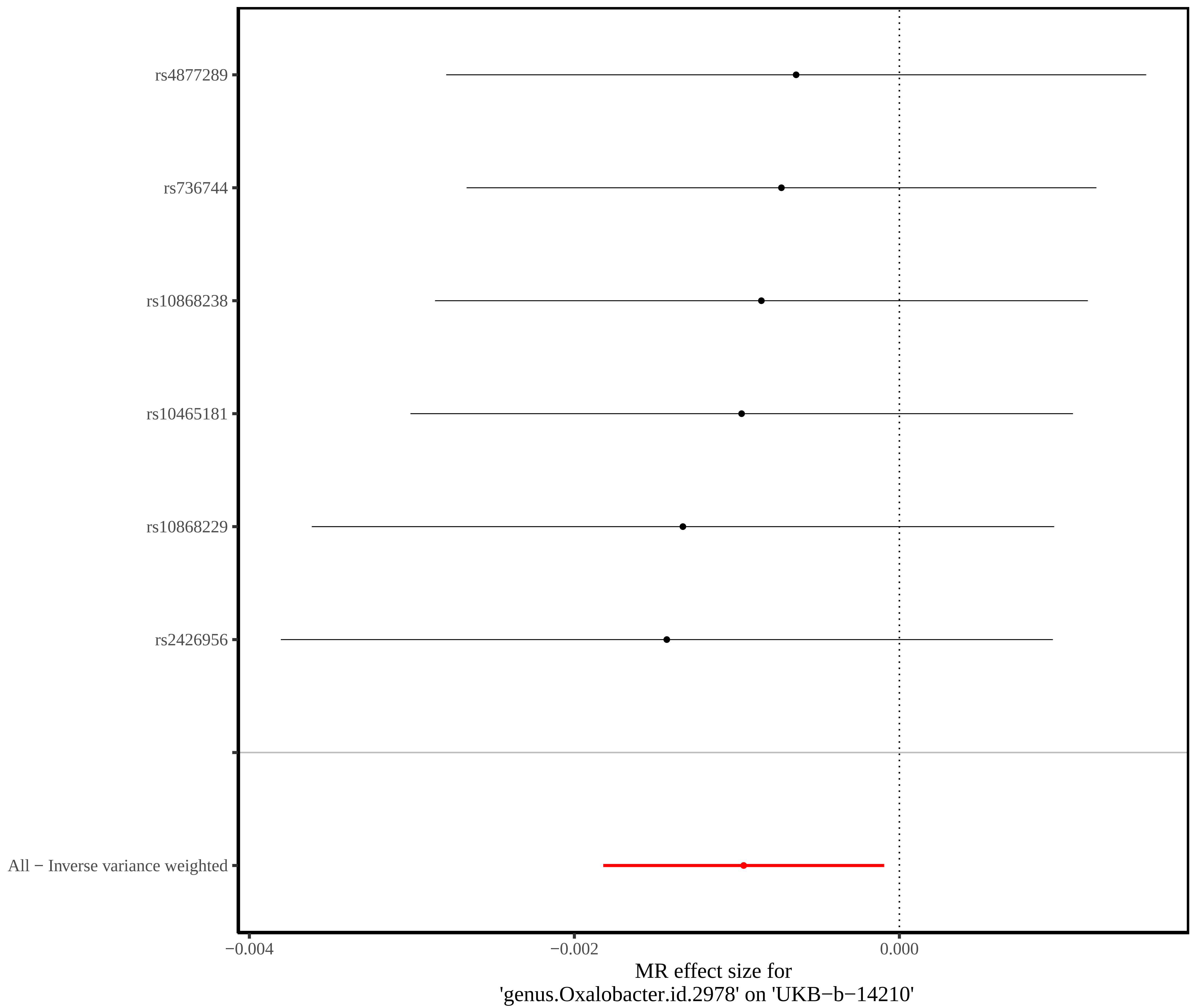
rs4877289
rs736744
rs10868238
rs10465181
rs10868229
rs2426956
All − Inverse variance weighted
−0.004
−0.002
0.000
MR effect size for
'genus.Oxalobacter.id.2978' on 'UKB−b−14210'

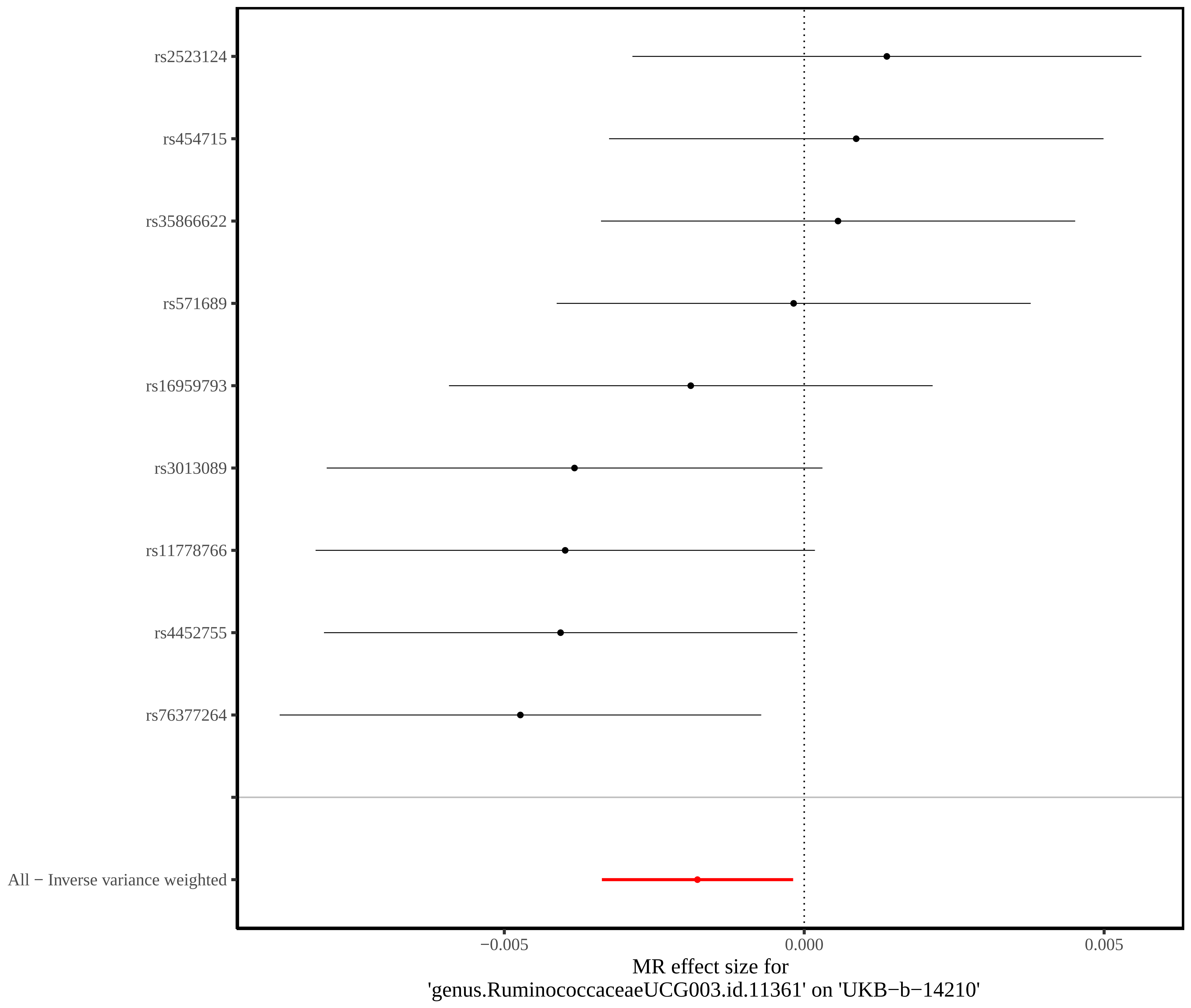
rs2523124
rs454715
rs35866622
rs571689
rs16959793
rs3013089
rs11778766
rs4452755
rs76377264
All − Inverse variance weighted
−0.005
0.000
0.005
MR effect size for
'genus.RuminococcaceaeUCG003.id.11361' on 'UKB−b−14210'

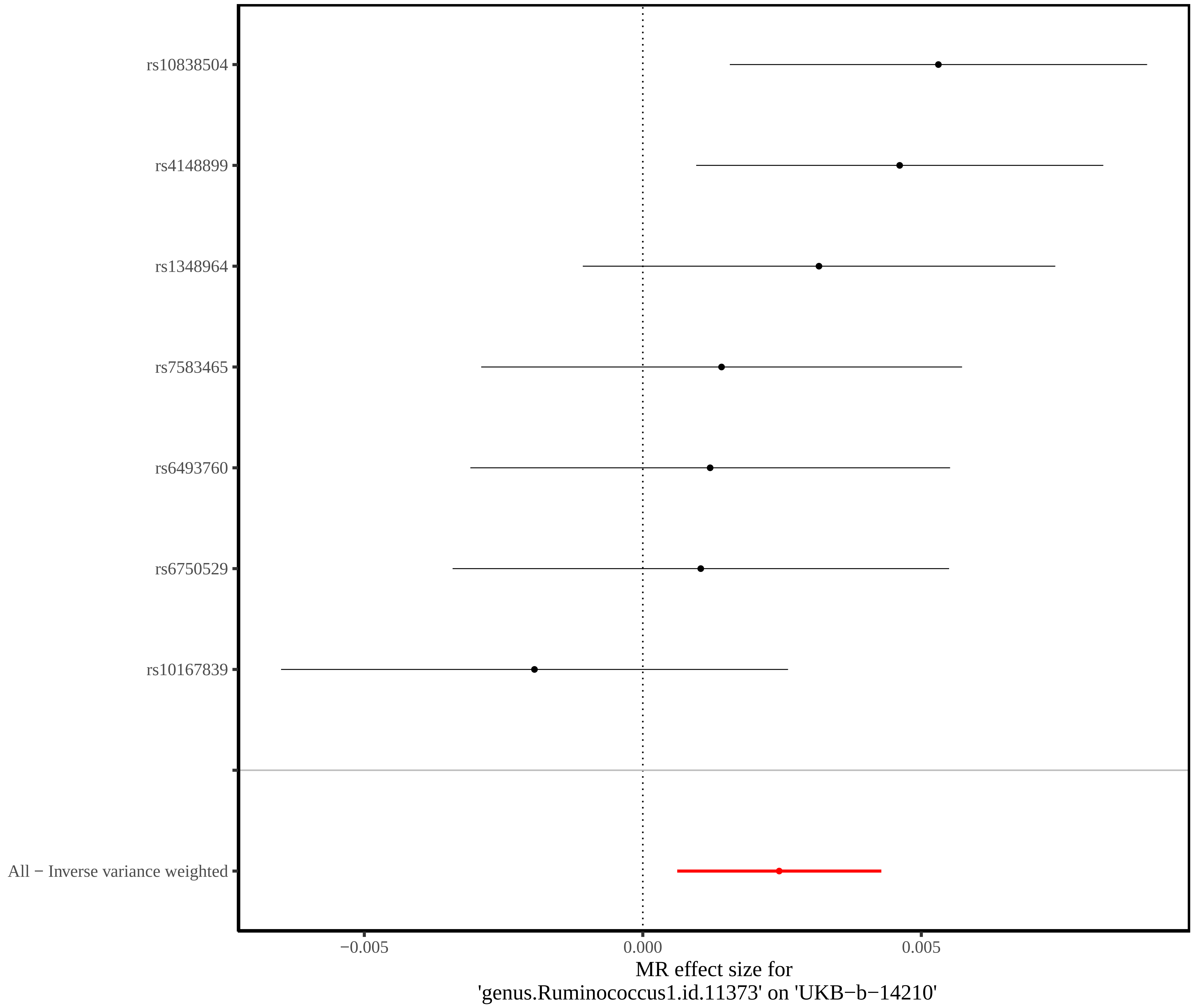
rs10838504
rs4148899
rs1348964
rs7583465
rs6493760
rs6750529
rs10167839
All − Inverse variance weighted
−0.005
0.000
0.005
MR effect size for
'genus.Ruminococcus1.id.11373' on 'UKB−b−14210'

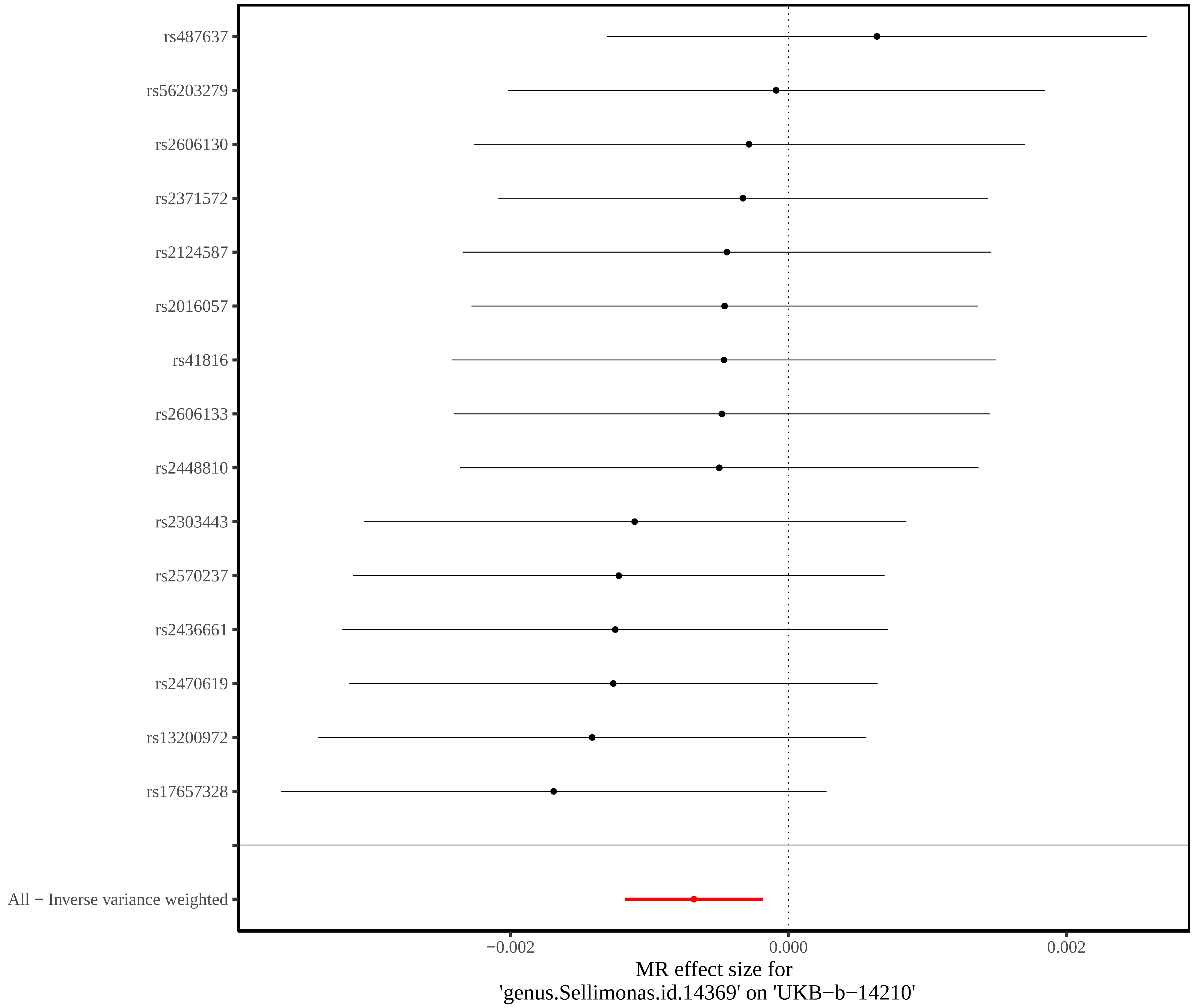
rs487637
rs56203279
rs2606130
rs2371572
rs2124587
rs2016057
rs41816
rs2606133
rs2448810
rs2303443
rs2570237
rs2436661
rs2470619
rs13200972
rs17657328
All − Inverse variance weighted
−0.002
0.000
0.002
MR effect size for
'genus.Sellimonas.id.14369' on 'UKB−b−14210'

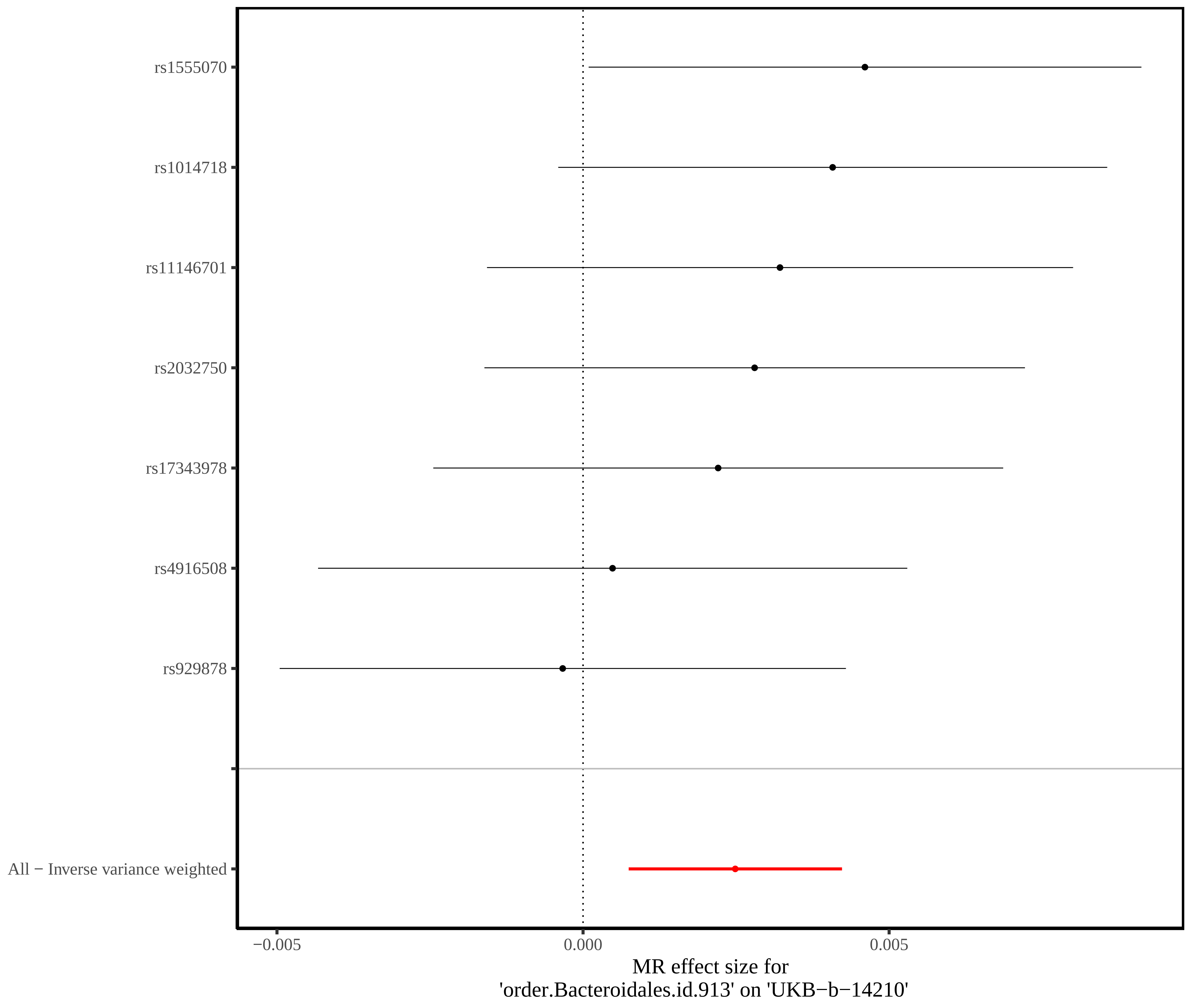

rs1555070
rs1014718
rs11146701
rs2032750
rs17343978
rs4916508
rs929878
All − Inverse variance weighted
−0.005
0.000
0.005
MR effect size for
'order.Bacteroidales.id.913' on 'UKB−b−14210'

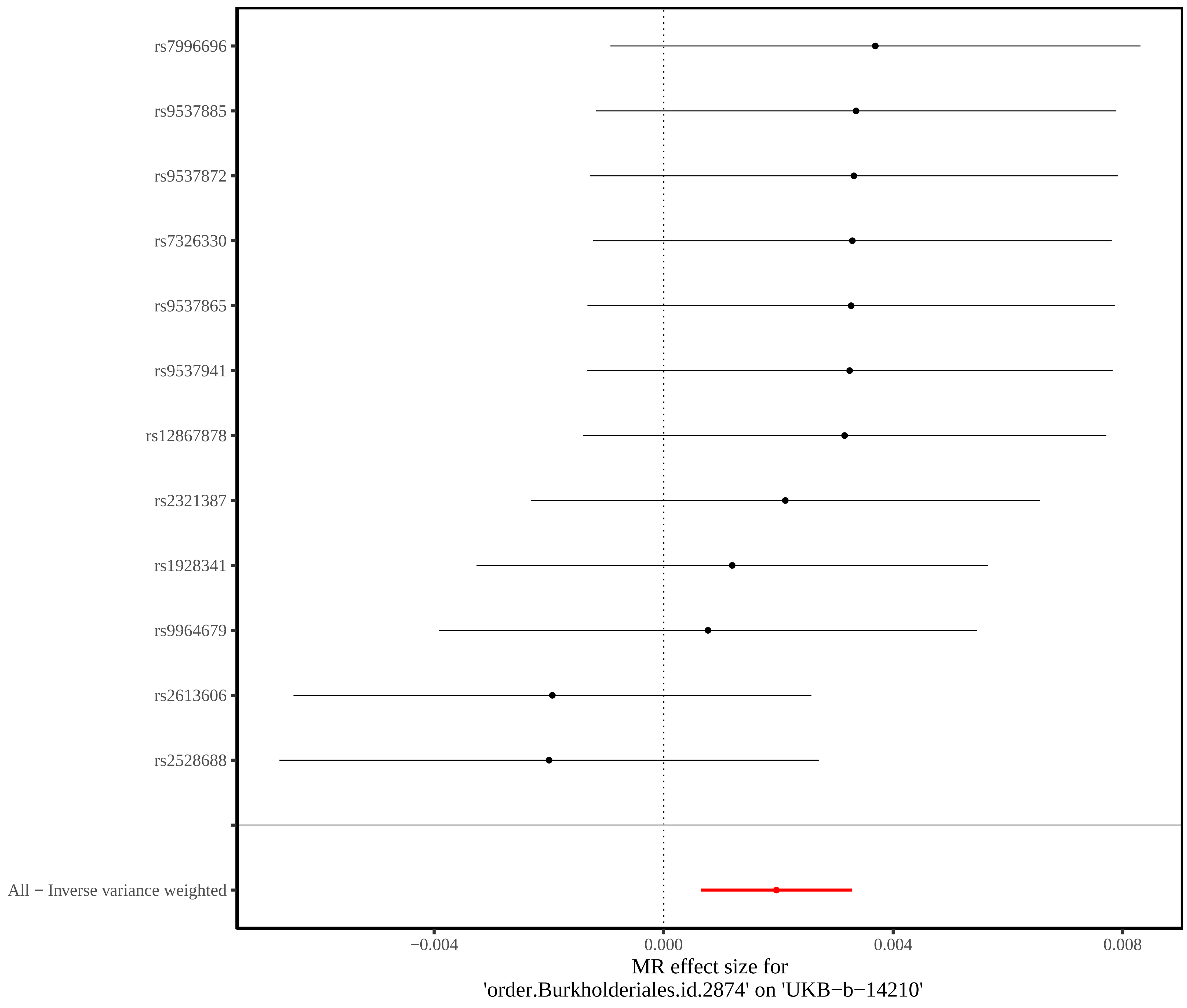
rs7996696
rs9537885
rs9537872
rs7326330
rs9537865
rs9537941
rs12867878
rs2321387
rs1928341
rs9964679
rs2613606
rs2528688
All − Inverse variance weighted
−0.004
0.000
0.004
0.008
MR effect size for
'order.Burkholderiales.id.2874' on 'UKB−b−14210'

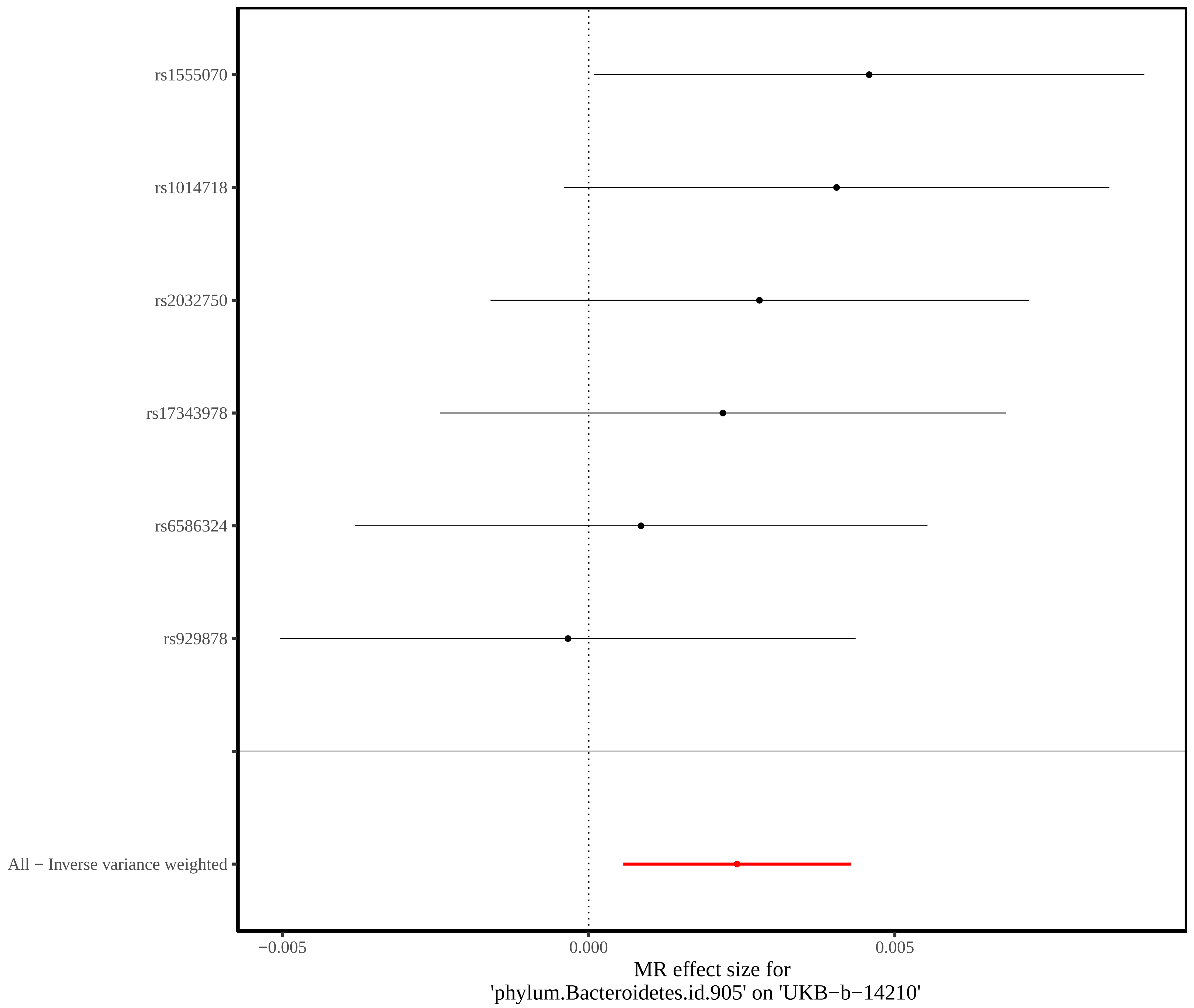
rs1555070
rs1014718
rs2032750
rs17343978
rs6586324
rs929878
All − Inverse variance weighted
−0.005
0.000
0.005
MR effect size for
'phylum.Bacteroidetes.id.905' on 'UKB−b−14210'

Supplementary Figure 3

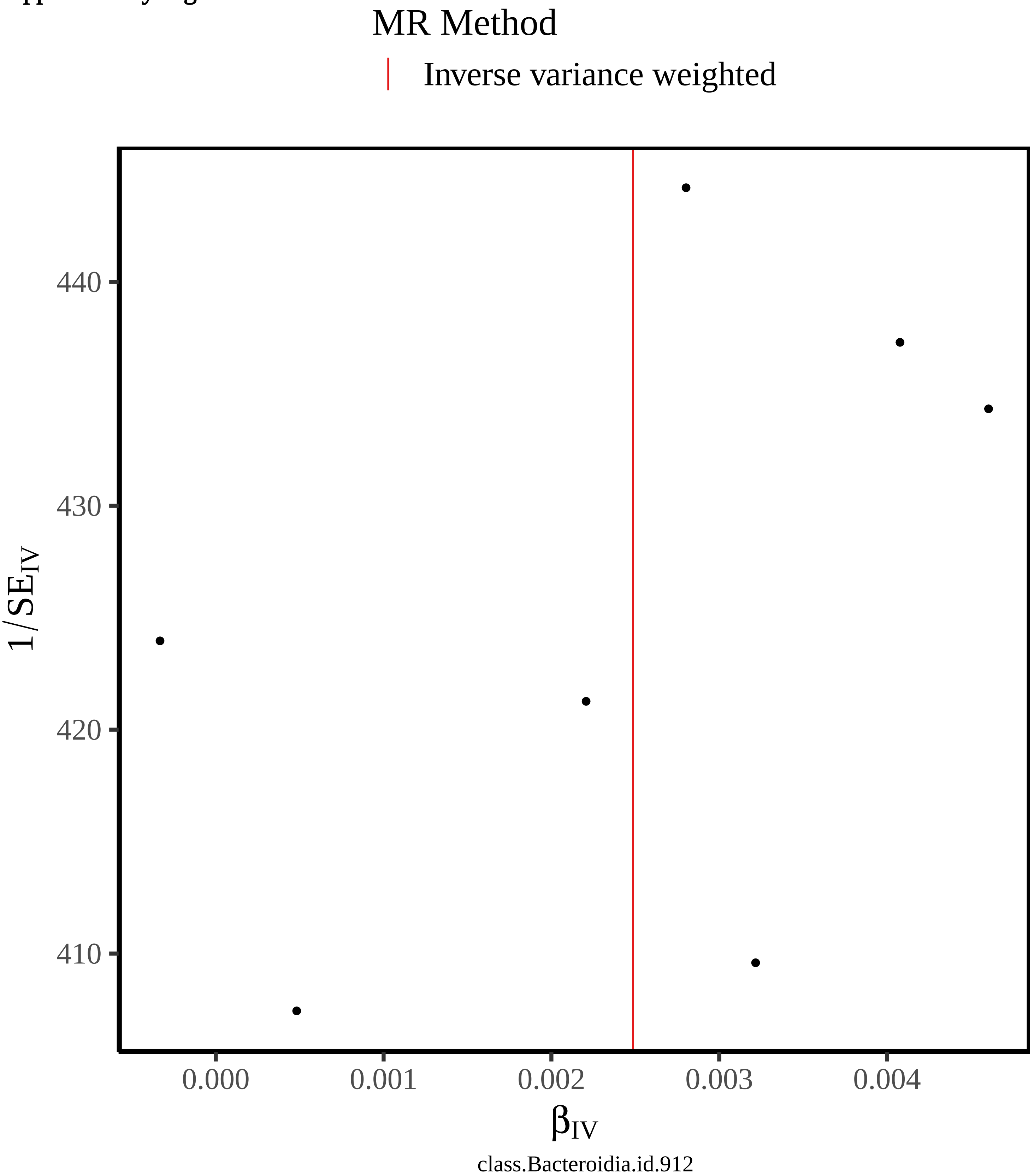

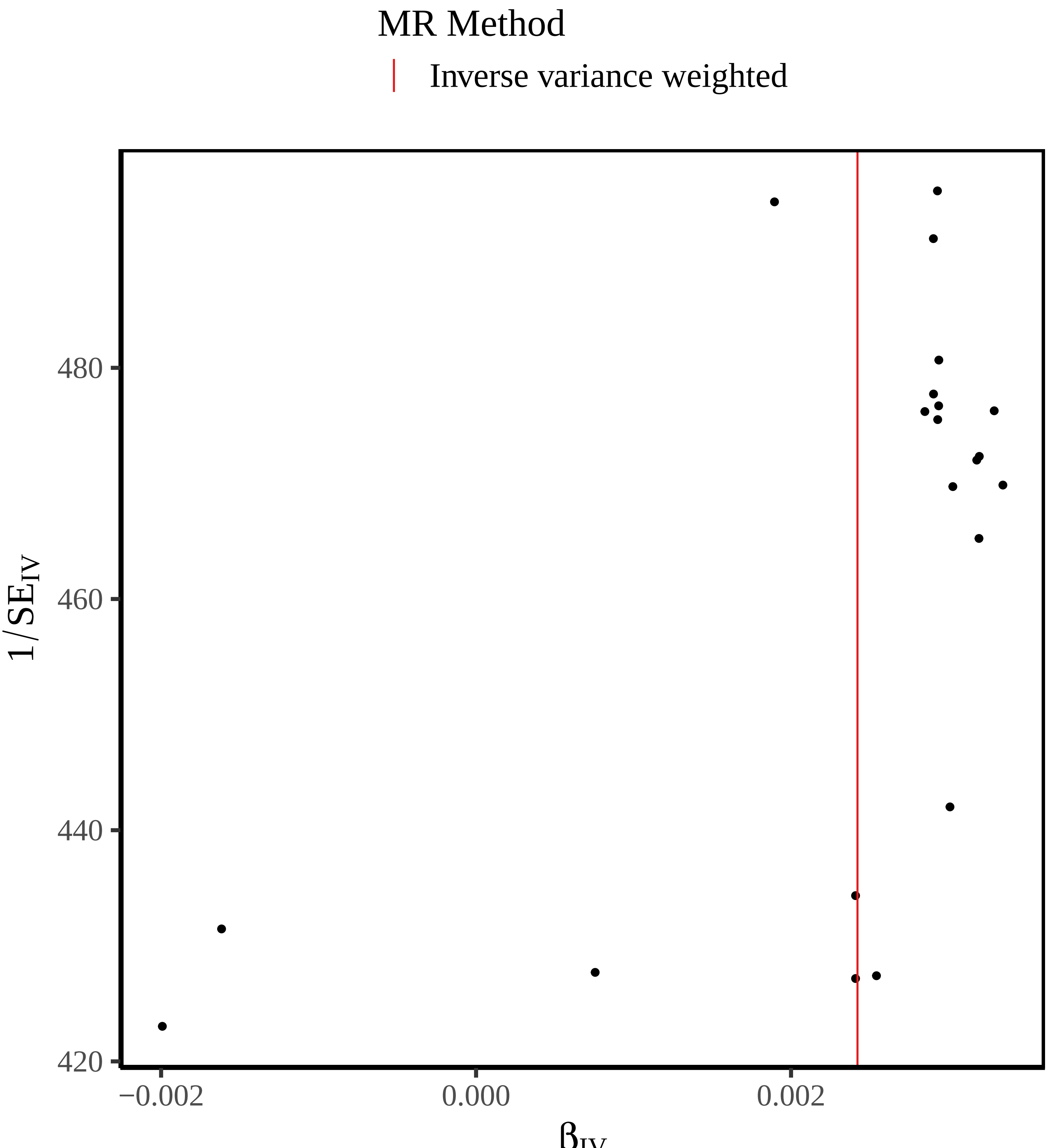

family.Alcaligenaceae.id.2875

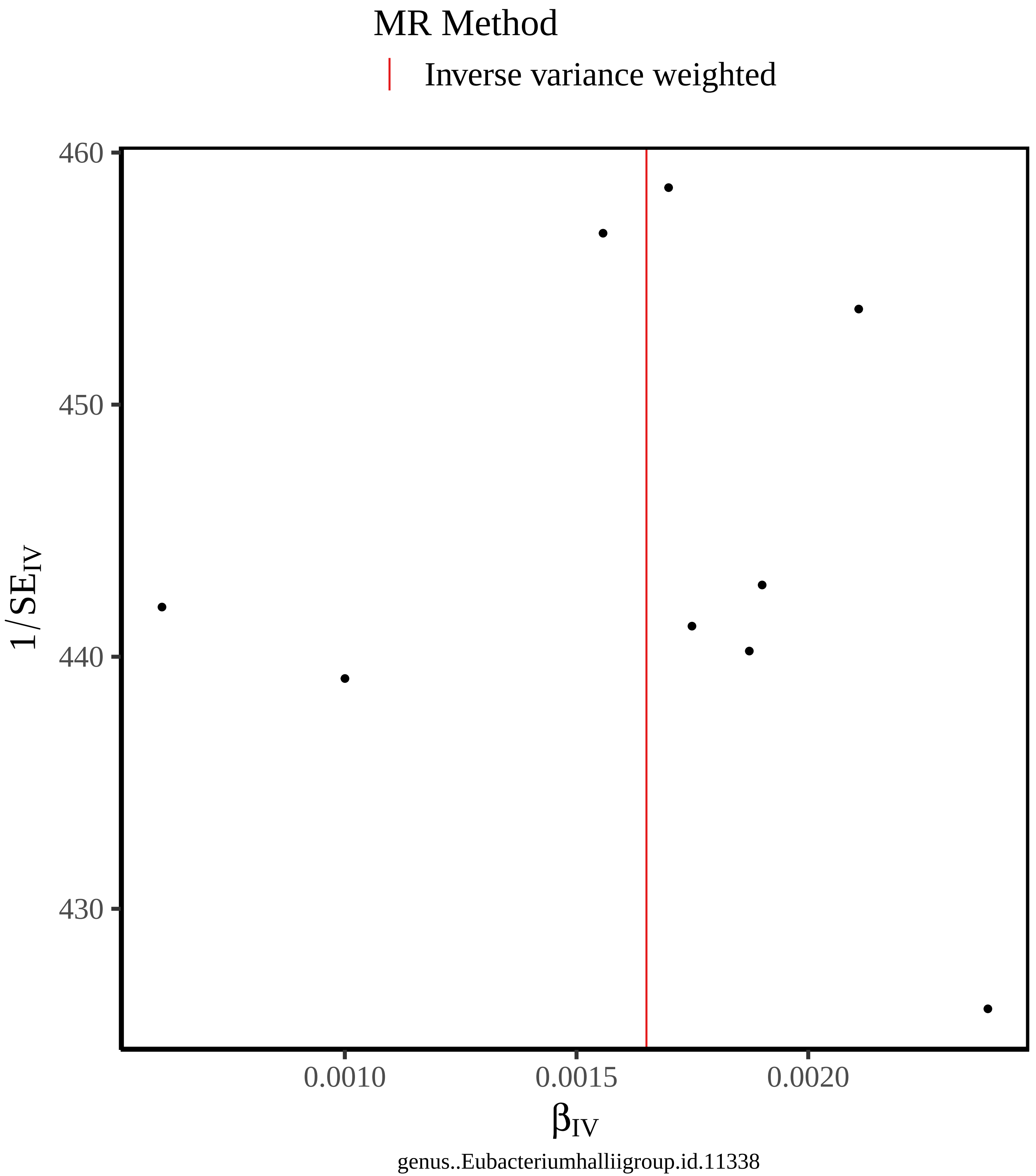
MR Method
Inverse variance weighted
460
450
440
430
1/SE$_{IV}$
0.0010
0.0015
0.0020
β$_{IV}$
genus..Eubacteriumhalliigroup.id.11338

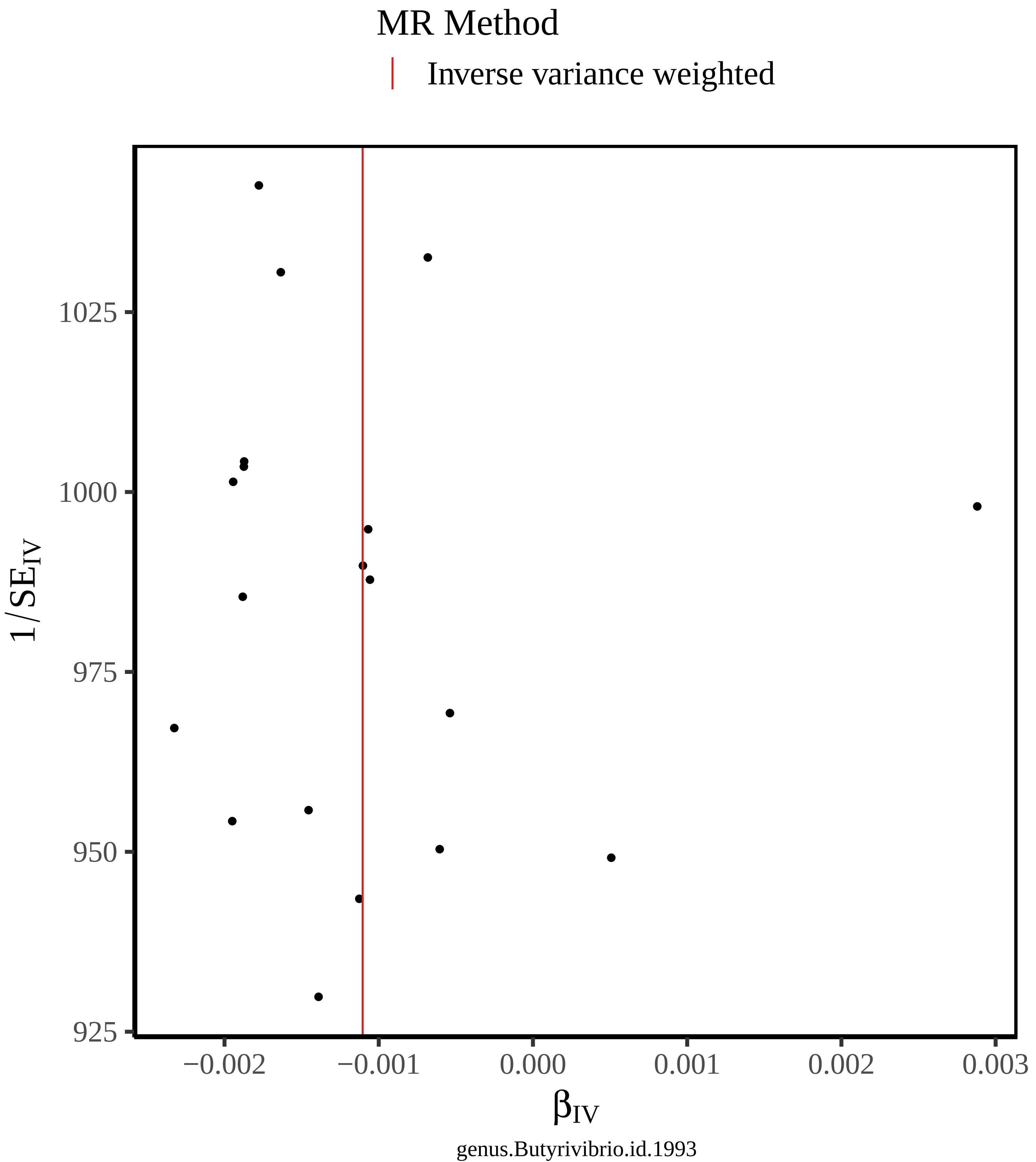
MR Method
Inverse variance weighted
1/SE_IV
1025
1000
975
950
925
−0.002
−0.001
0.000
0.001
0.002
0.003
β_IV
genus.Butyrivibrio.id.1993

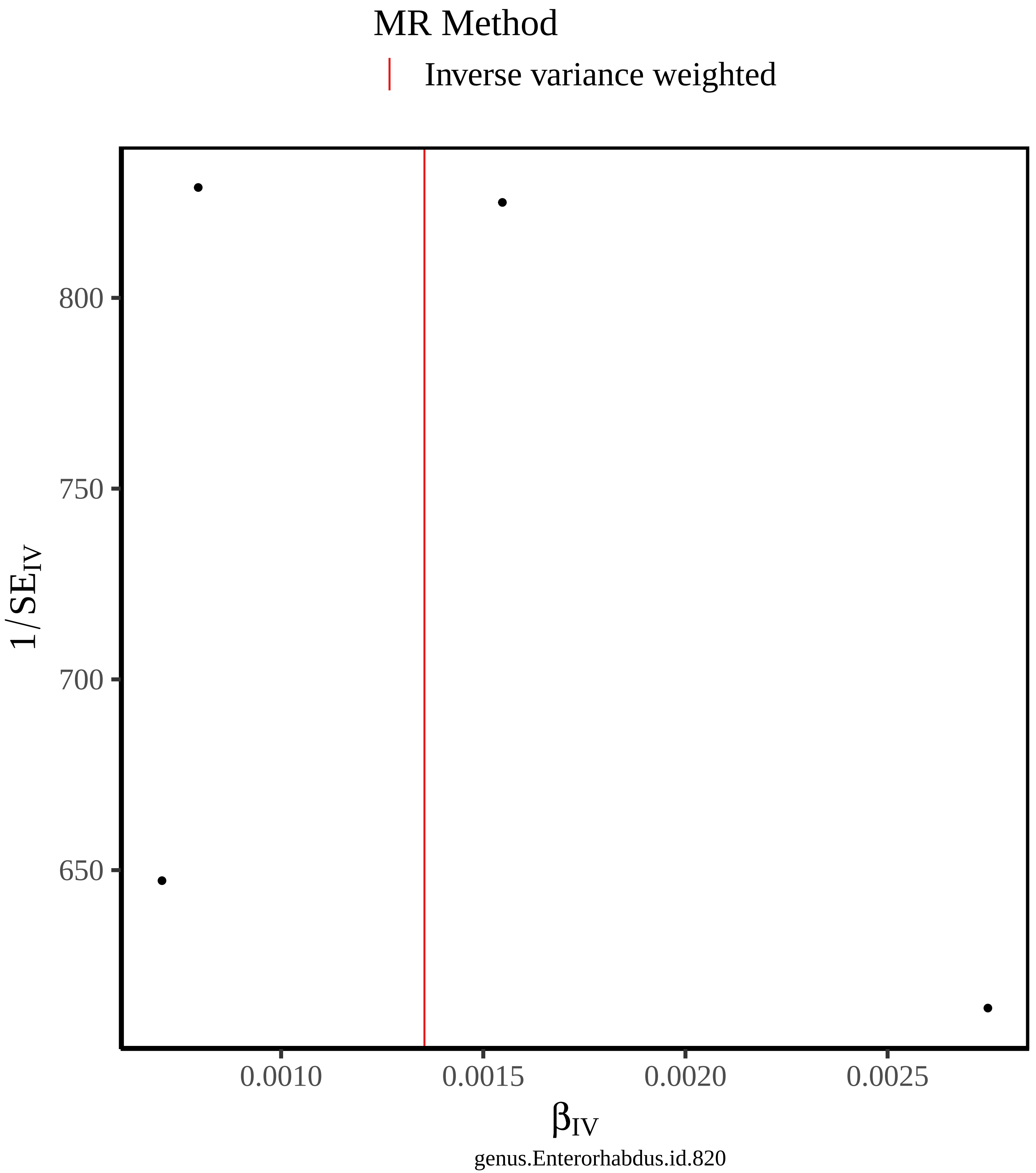

MR Method
Inverse variance weighted
800
750
700
650
$1/SE_{IV}$
0.0010
0.0015
0.0020
0.0025
β_{IV}
genus.Enterorhabdus.id.820

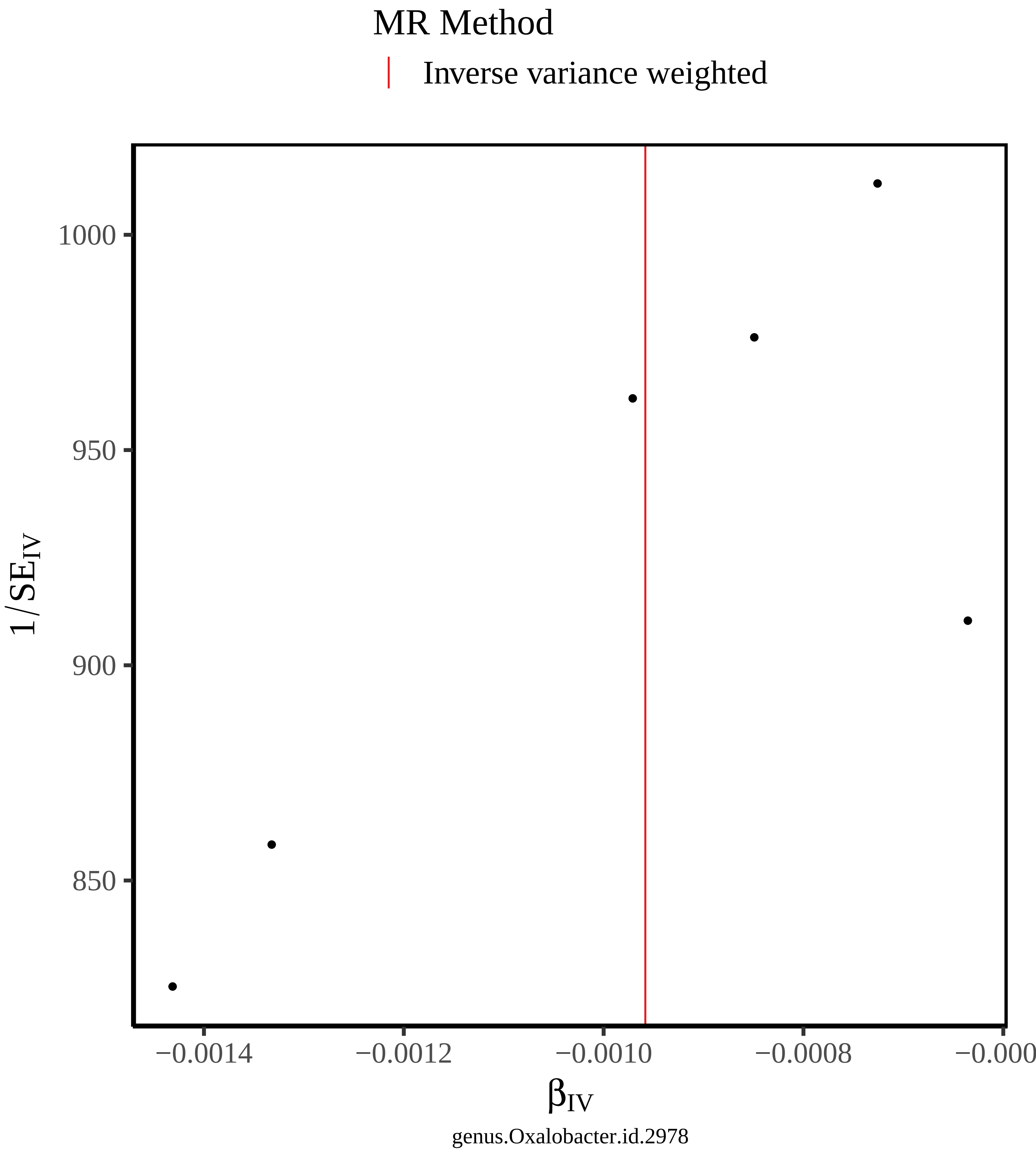
MR Method
Inverse variance weighted
1000
950
900
850
1/SE_IV
−0.0014
−0.0012
−0.0010
−0.0008
−0.000
β_IV
genus.Oxalobacter.id.2978

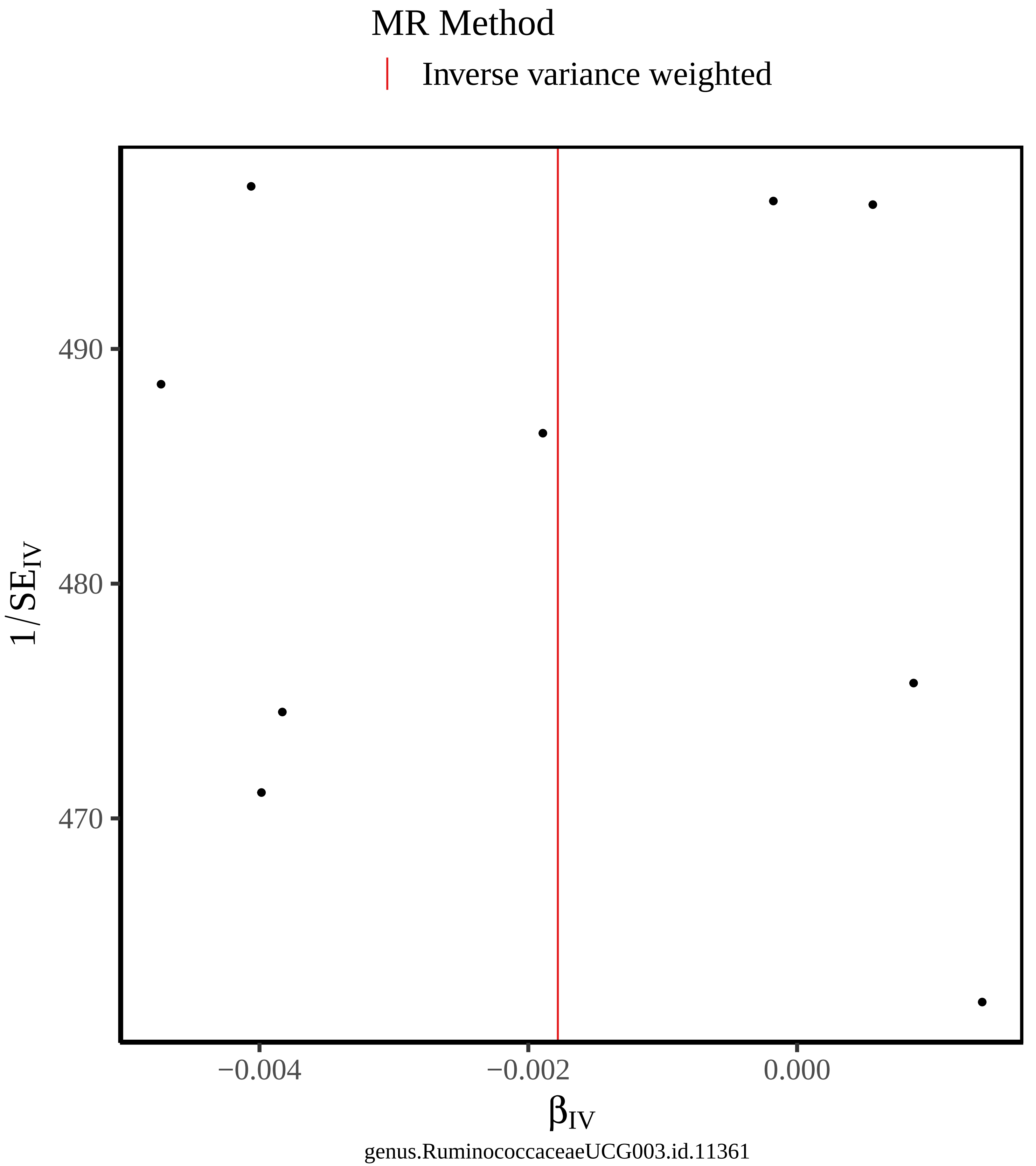
MR Method
Inverse variance weighted
490
480
470
1/SE$_{IV}$
−0.004
−0.002
0.000
β$_{IV}$
genus.RuminococcaceaeUCG003.id.11361

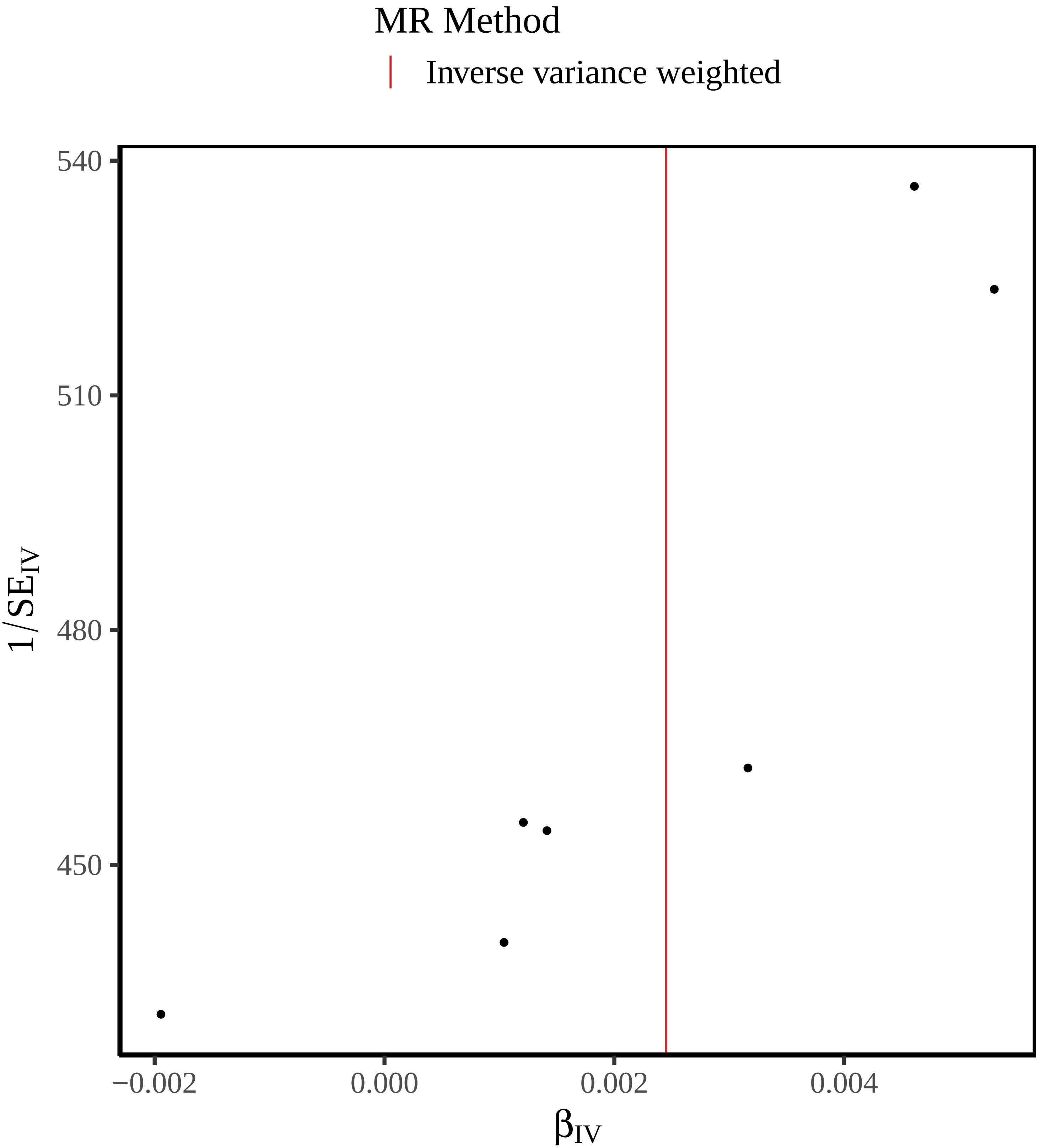

genus.Ruminococcus1.id.11373

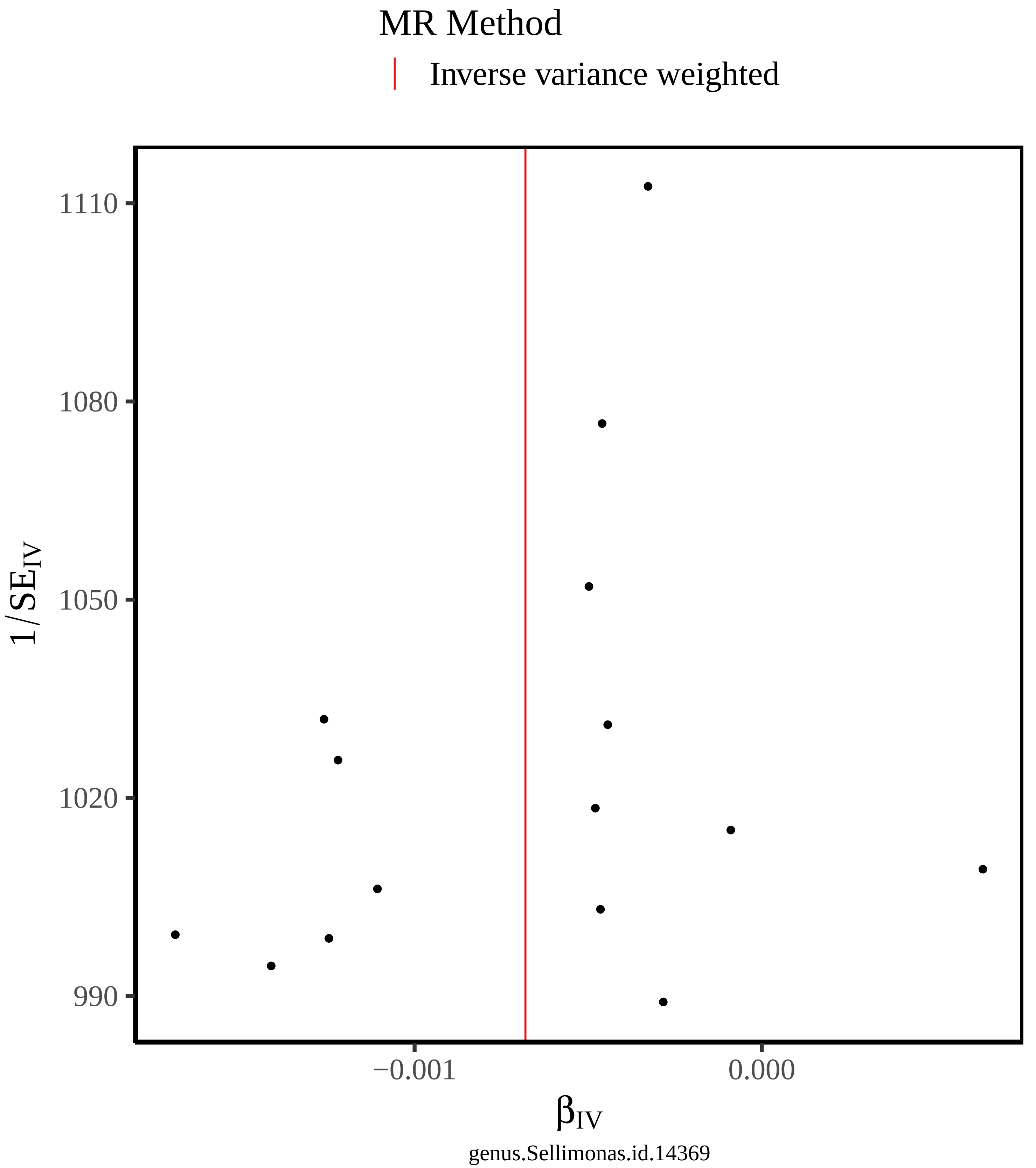
MR Method
Inverse variance weighted
1110
1080
1050
1020
990
1/SE$_{IV}$
−0.001
0.000
β$_{IV}$
genus.Sellimonas.id.14369

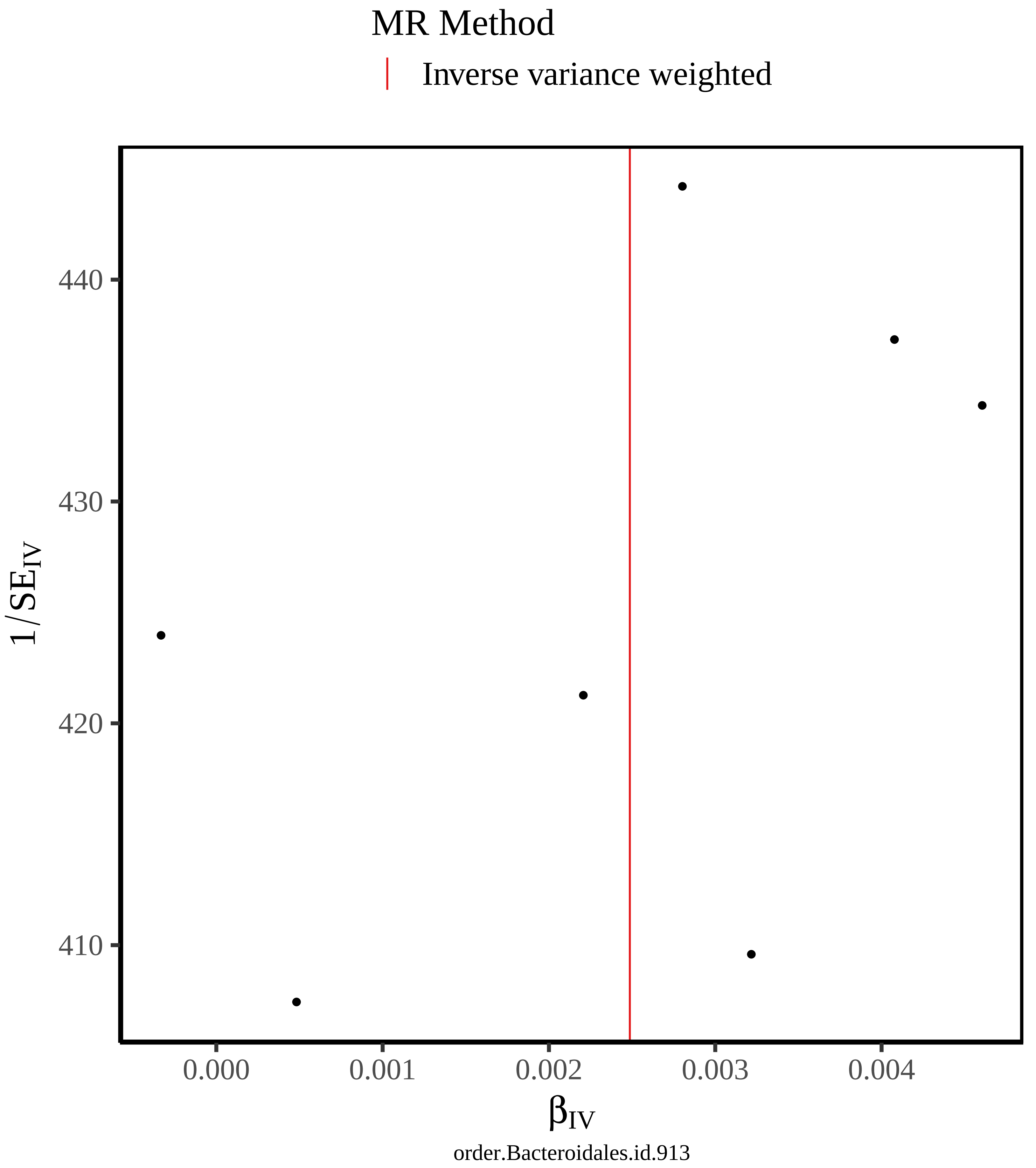
MR Method
Inverse variance weighted
440
430
420
410
$1/SE_{IV}$
0.000
0.001
0.002
0.003
0.004
β_{IV}
order.Bacteroidales.id.913

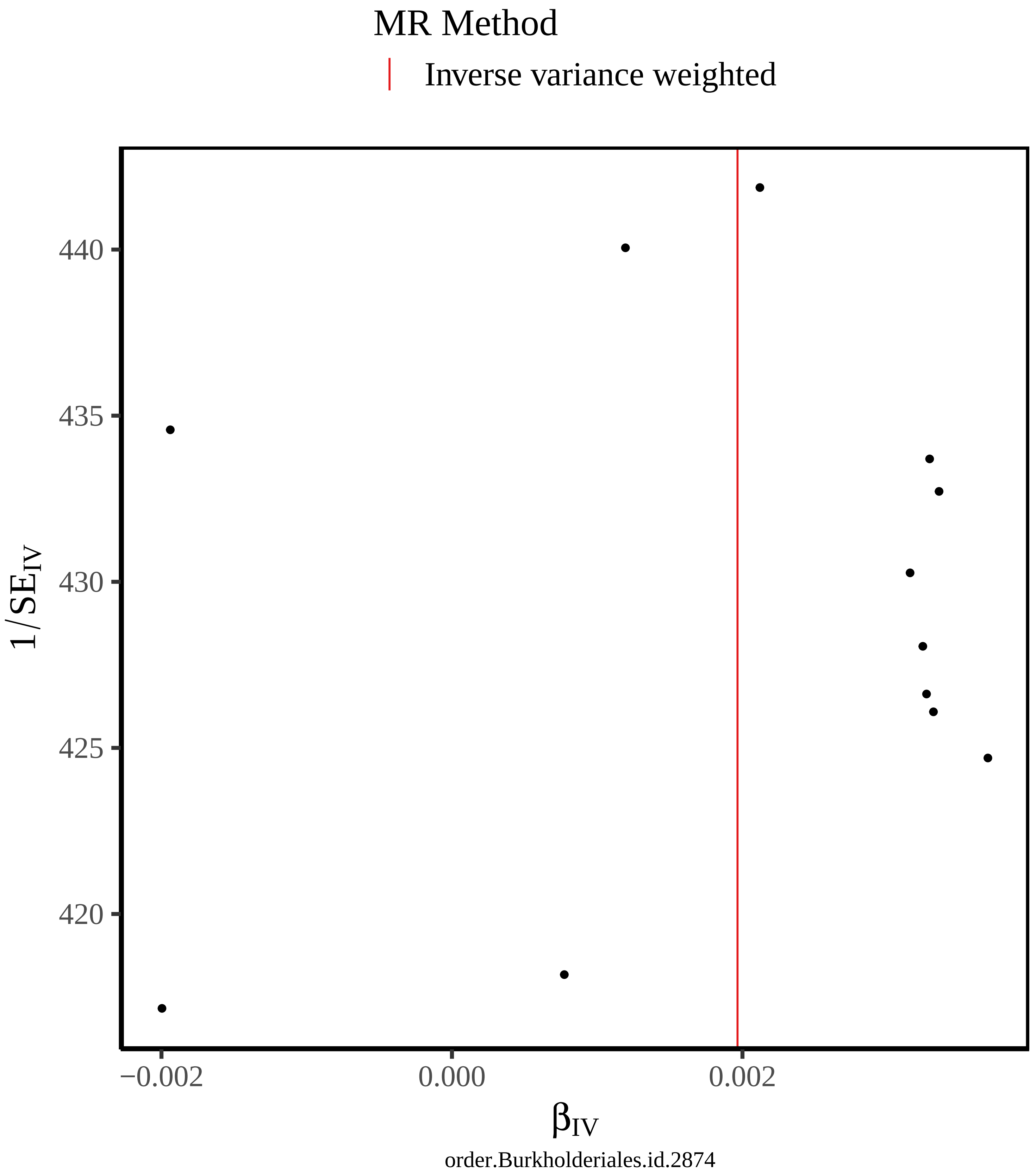
MR Method
Inverse variance weighted
440
435
430
425
420
1/SE_IV
−0.002
0.000
0.002
β_IV
order.Burkholderiales.id.2874

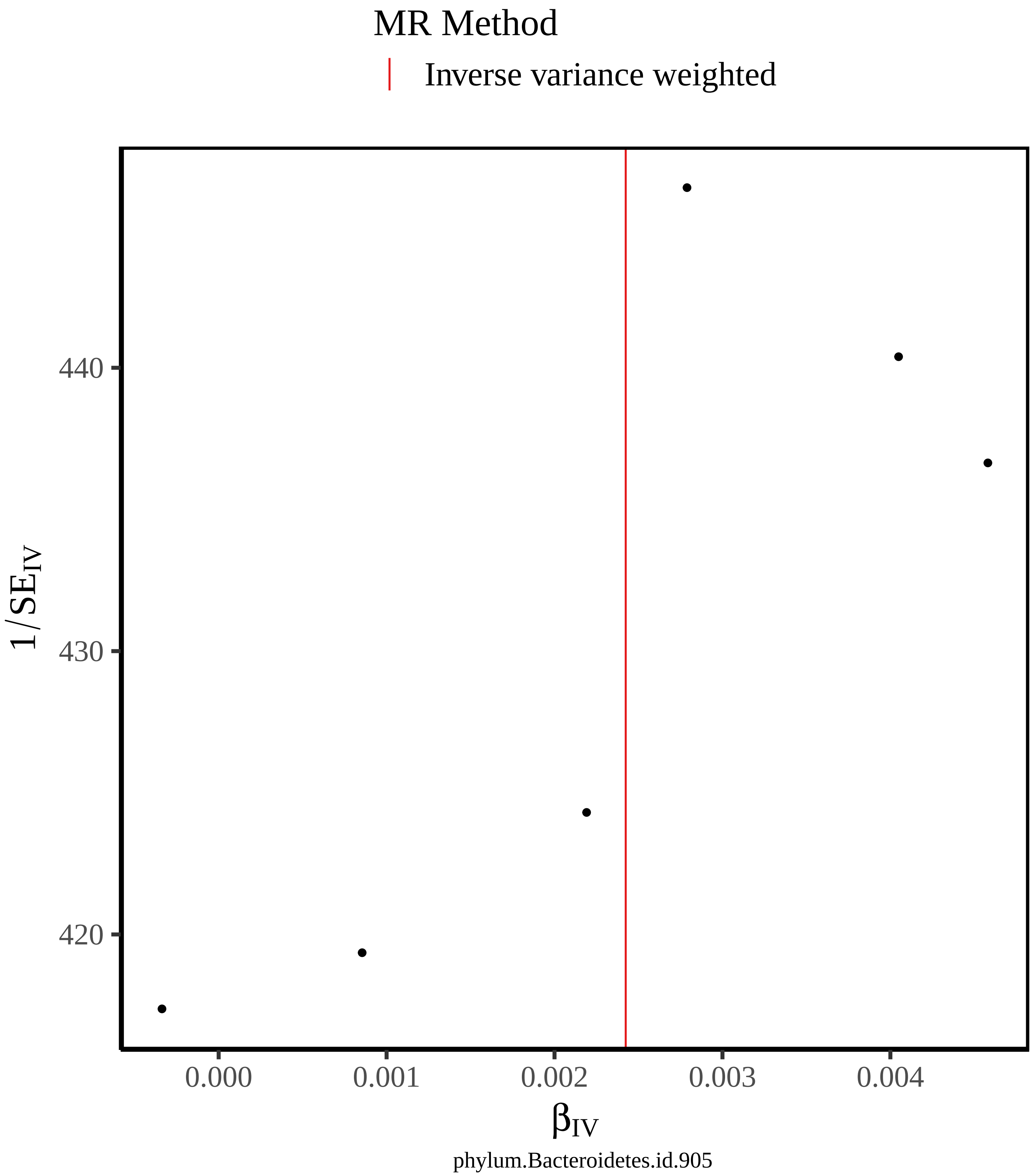
MR Method
Inverse variance weighted
1/SE$_{IV}$
440
430
420
0.000
0.001
0.002
0.003
0.004
β$_{IV}$
phylum.Bacteroidetes.id.905

Supplementary Figure 4

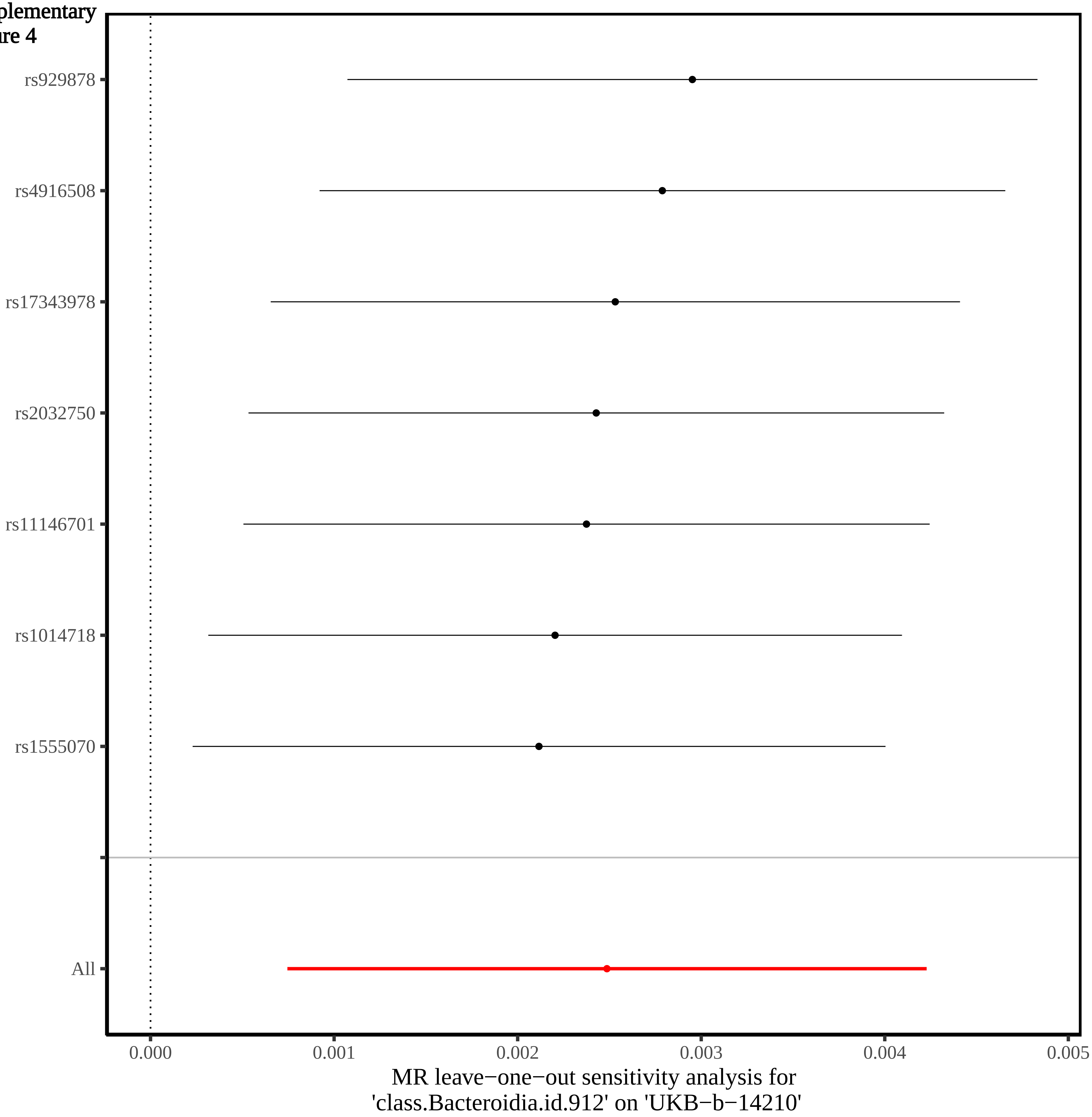

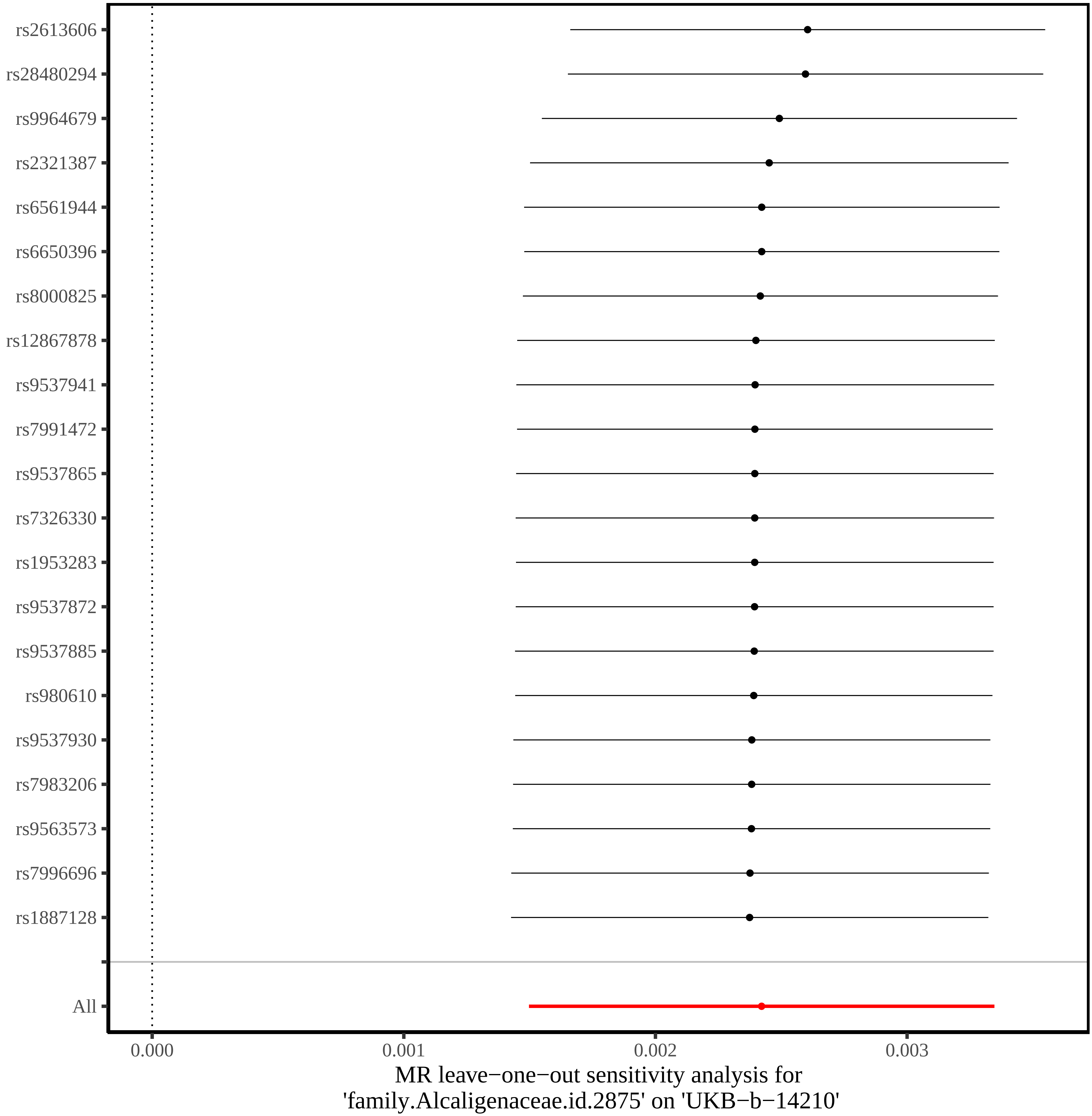
rs2613606
rs28480294
rs9964679
rs2321387
rs6561944
rs6650396
rs8000825
rs12867878
rs9537941
rs7991472
rs9537865
rs7326330
rs1953283
rs9537872
rs9537885
rs980610
rs9537930
rs7983206
rs9563573
rs7996696
rs1887128
All
0.000
0.001
0.002
0.003
MR leave−one−out sensitivity analysis for
'family.Alcaligenaceae.id.2875' on 'UKB−b−14210'

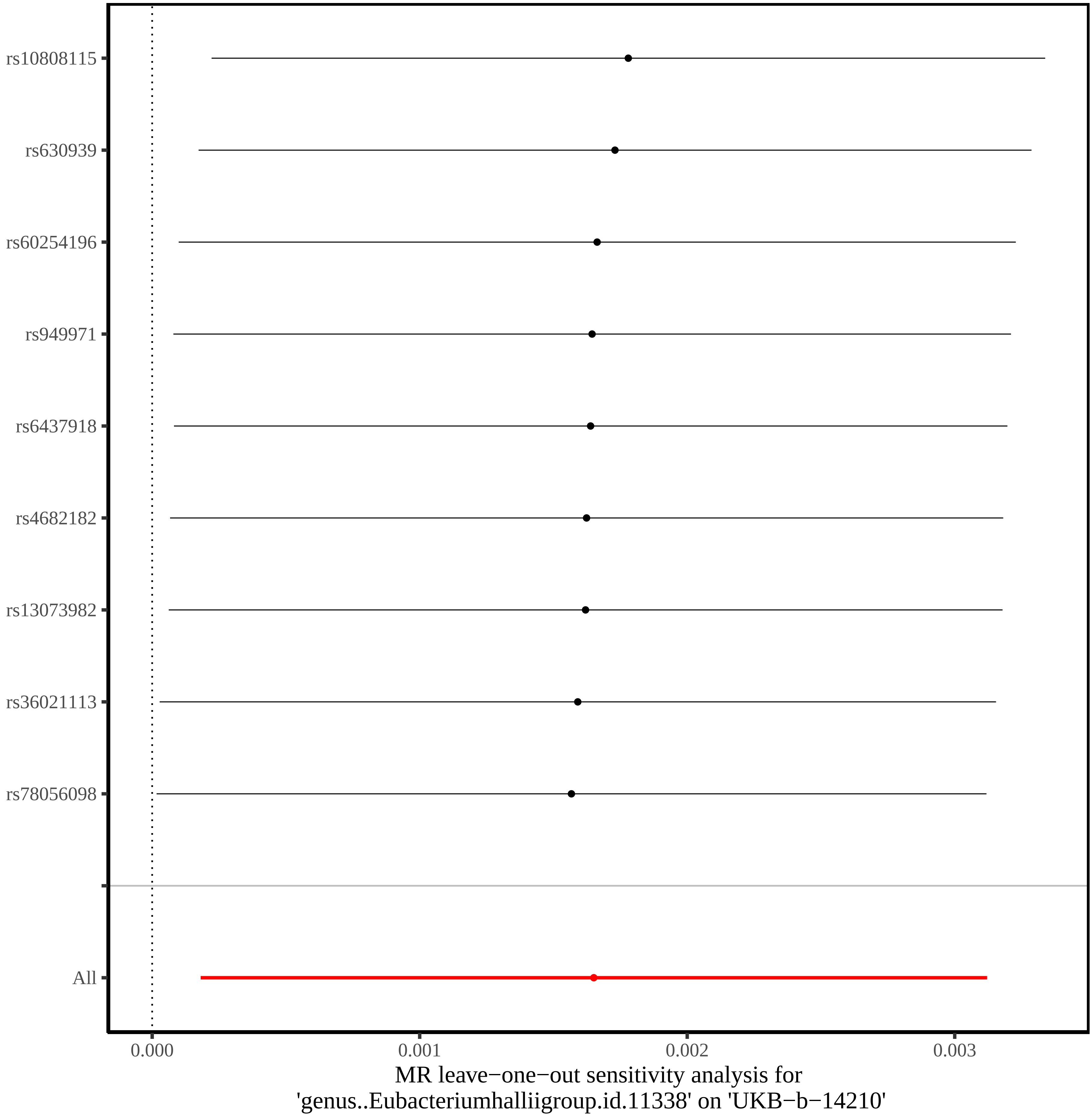
rs10808115
rs630939
rs60254196
rs949971
rs6437918
rs4682182
rs13073982
rs36021113
rs78056098
All
0.000
0.001
0.002
0.003
MR leave−one−out sensitivity analysis for
'genus..Eubacteriumhalliigroup.id.11338' on 'UKB−b−14210'

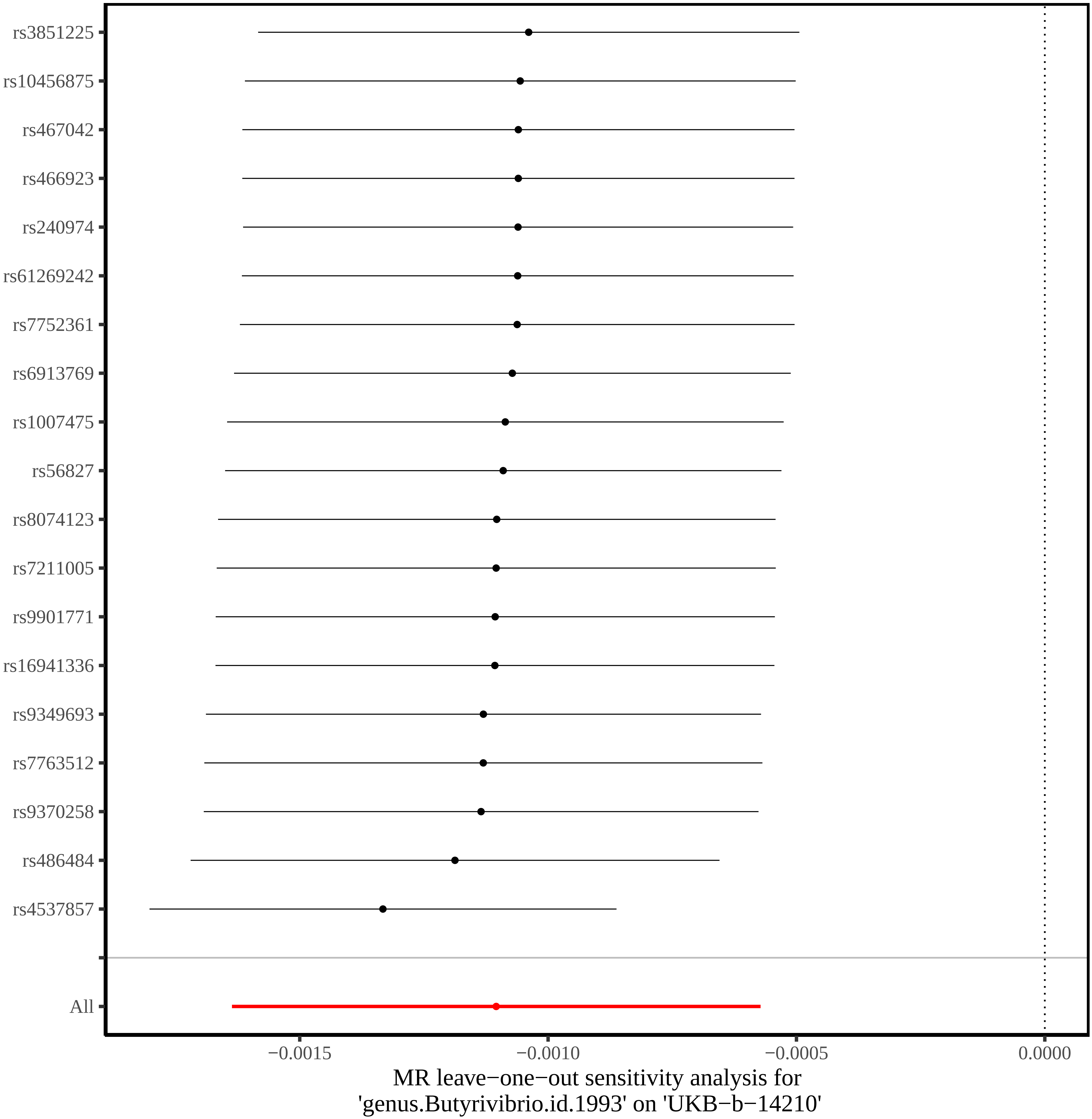

rs3851225
rs10456875
rs467042
rs466923
rs240974
rs61269242
rs7752361
rs6913769
rs1007475
rs56827
rs8074123
rs7211005
rs9901771
rs16941336
rs9349693
rs7763512
rs9370258
rs486484
rs4537857
All
−0.0015
−0.0010
−0.0005
0.0000
MR leave−one−out sensitivity analysis for
'genus.Butyrivibrio.id.1993' on 'UKB−b−14210'

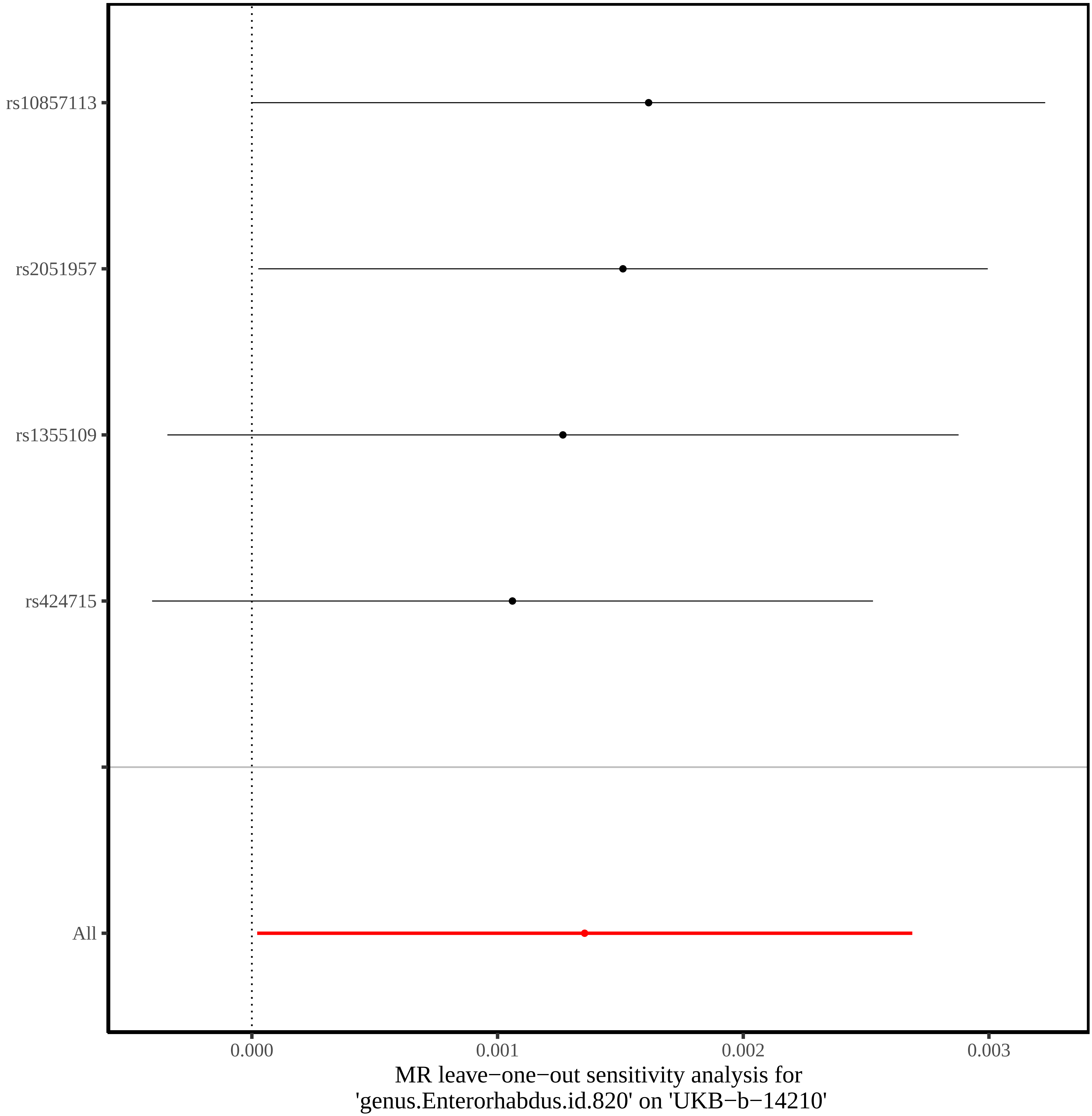
rs10857113
rs2051957
rs1355109
rs424715
All
0.000
0.001
0.002
0.003
MR leave−one−out sensitivity analysis for
'genus.Enterorhabdus.id.820' on 'UKB−b−14210'

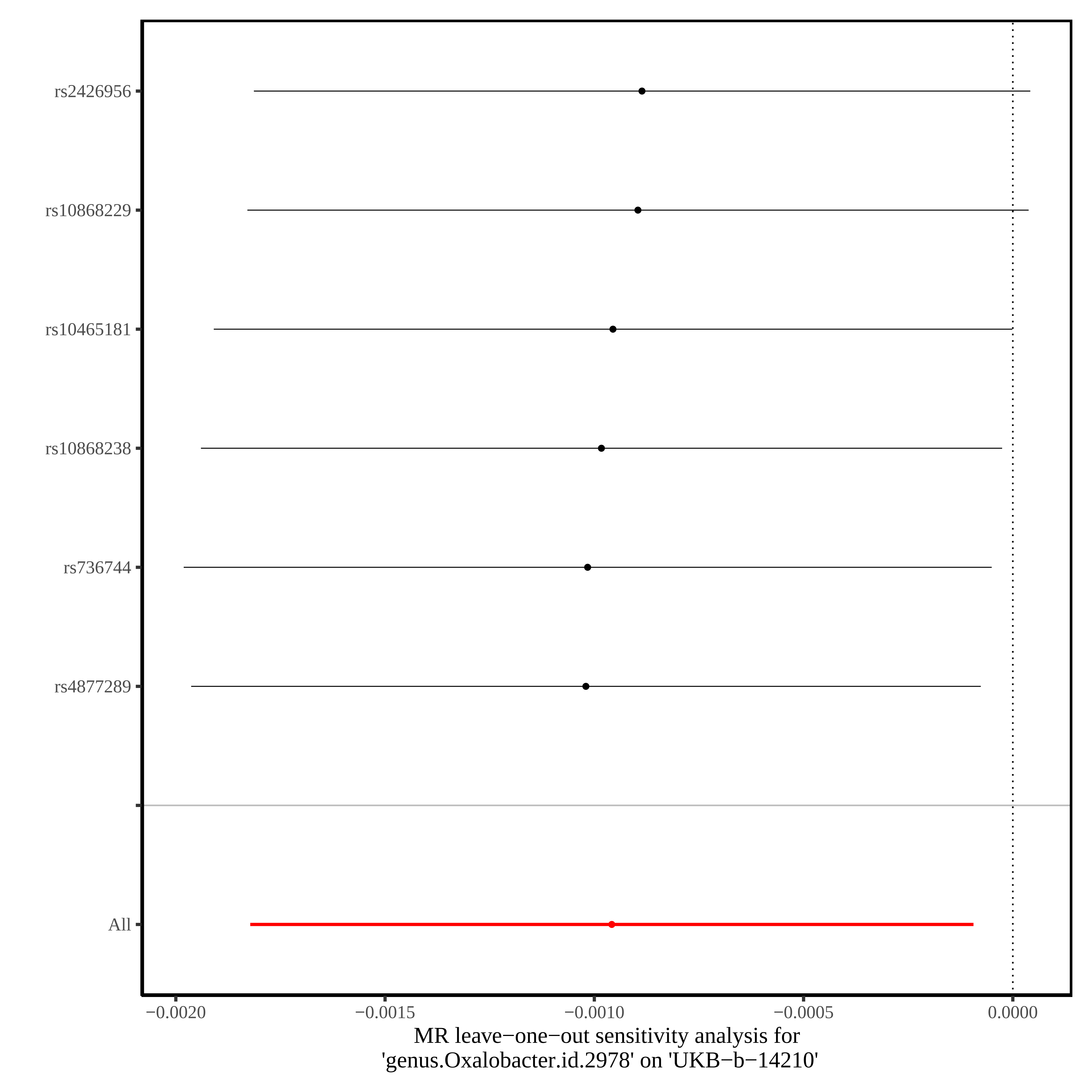

rs2426956
rs10868229
rs10465181
rs10868238
rs736744
rs4877289
All
−0.0020
−0.0015
−0.0010
−0.0005
0.0000
MR leave−one−out sensitivity analysis for
'genus.Oxalobacter.id.2978' on 'UKB−b−14210'

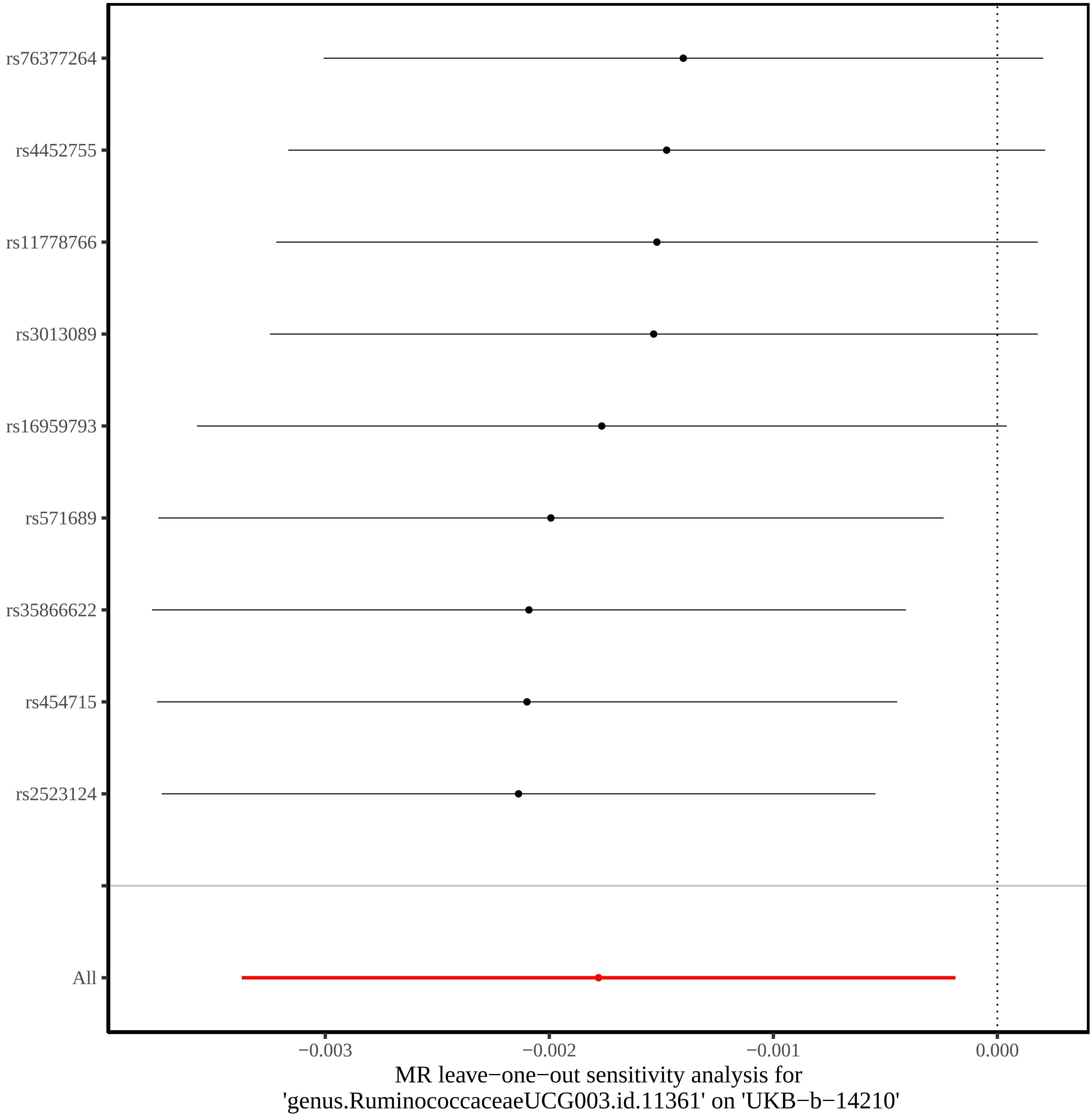
rs76377264
rs4452755
rs11778766
rs3013089
rs16959793
rs571689
rs35866622
rs454715
rs2523124
All
−0.003
−0.002
−0.001
0.000
MR leave−one−out sensitivity analysis for
'genus.RuminococcaceaeUCG003.id.11361' on 'UKB−b−14210'

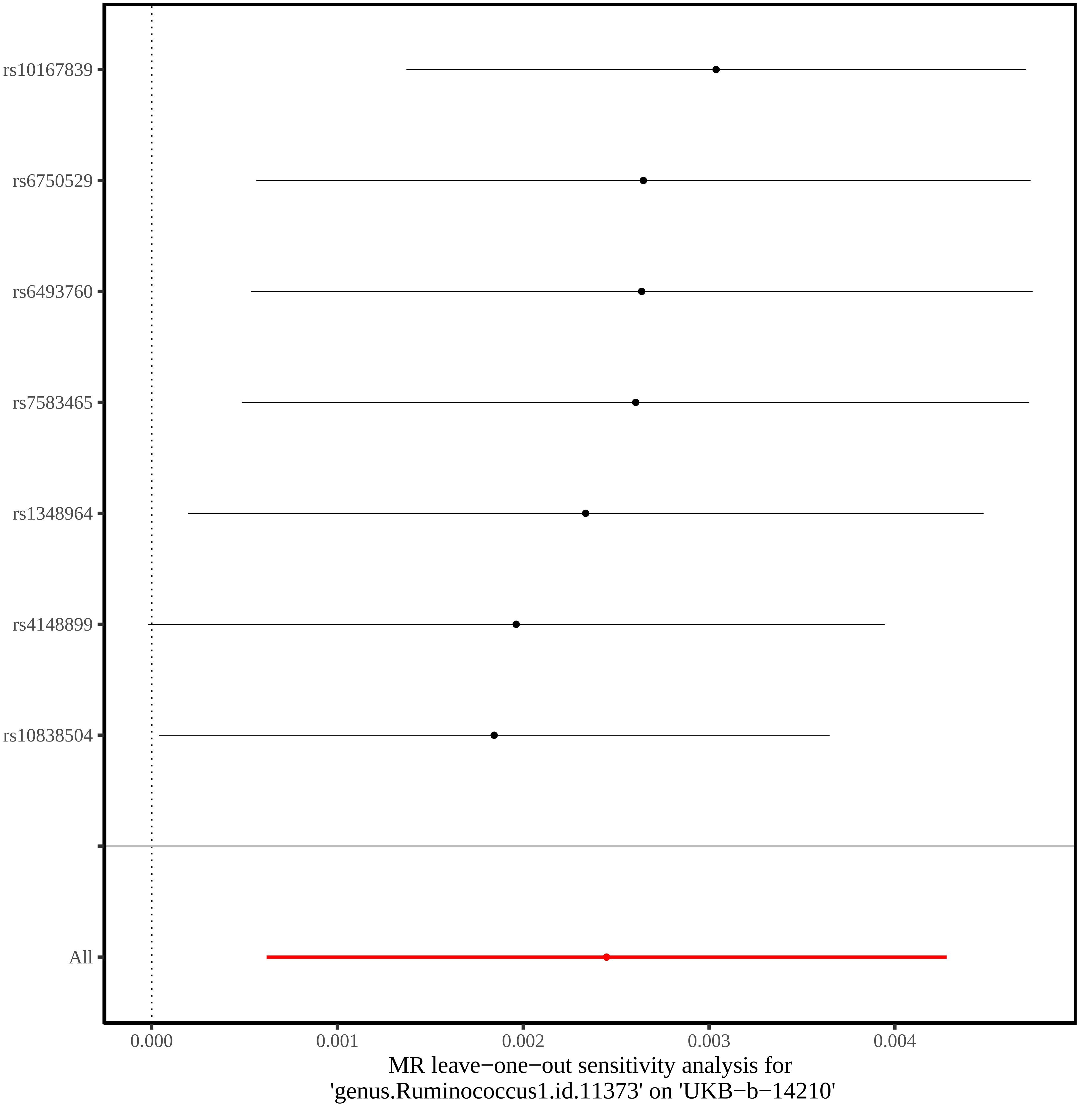

rs10167839
rs6750529
rs6493760
rs7583465
rs1348964
rs4148899
rs10838504
All
0.000
0.001
0.002
0.003
0.004
MR leave−one−out sensitivity analysis for
'genus.Ruminococcus1.id.11373' on 'UKB−b−14210'

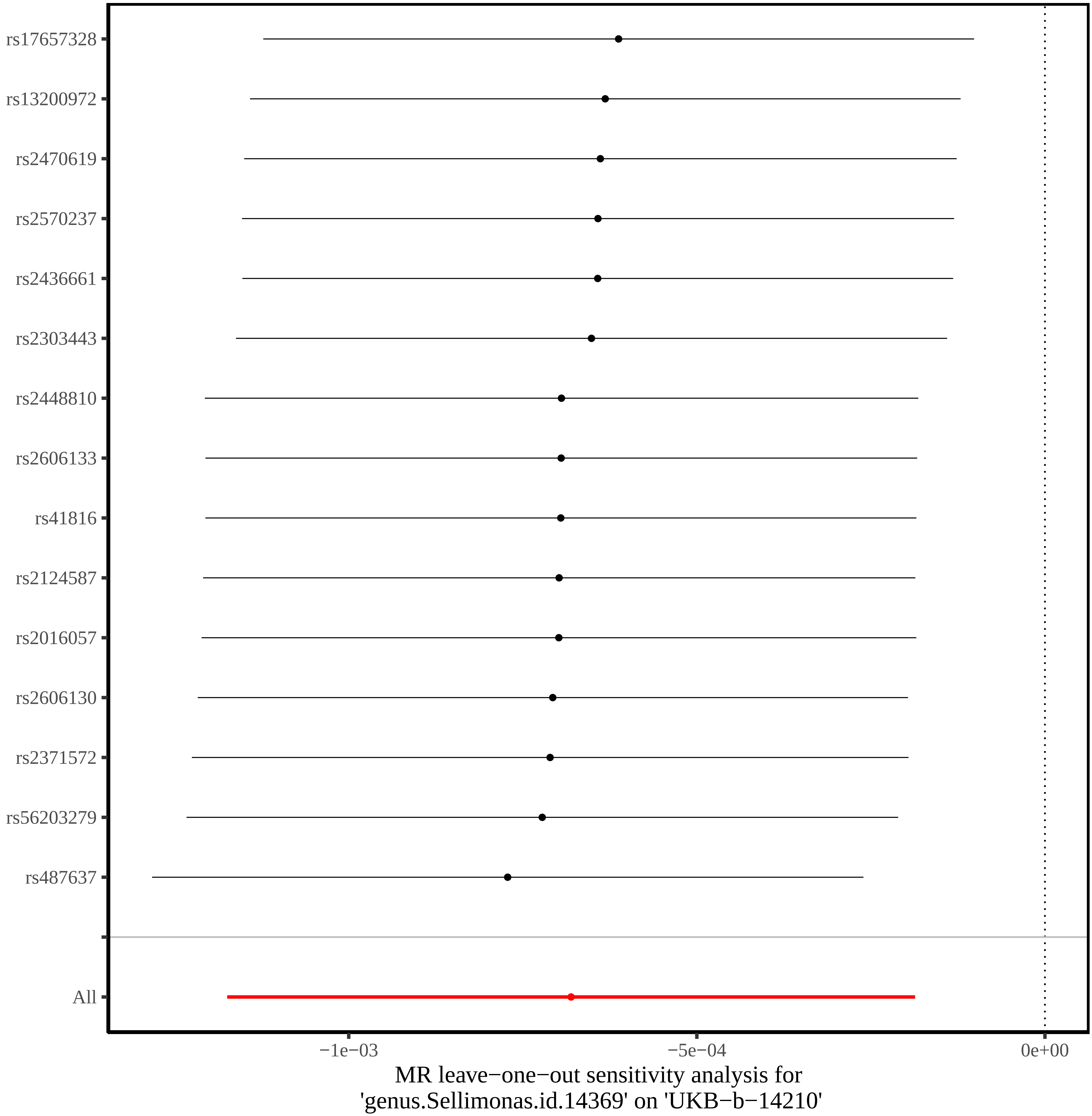

rs17657328
rs13200972
rs2470619
rs2570237
rs2436661
rs2303443
rs2448810
rs2606133
rs41816
rs2124587
rs2016057
rs2606130
rs2371572
rs56203279
rs487637
All
−1e−03
−5e−04
0e+00
MR leave−one−out sensitivity analysis for
'genus.Sellimonas.id.14369' on 'UKB−b−14210'

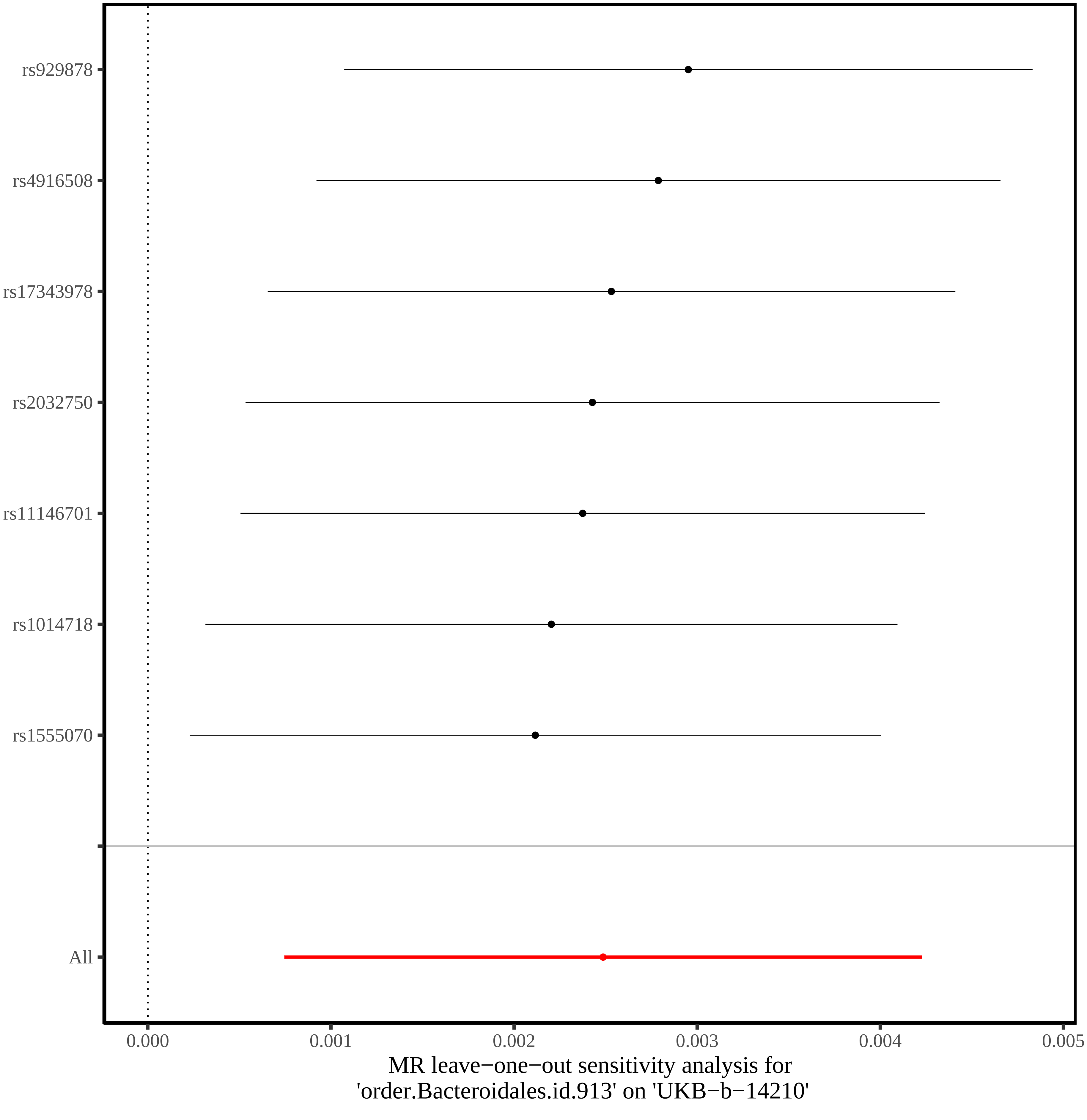
rs929878
rs4916508
rs17343978
rs2032750
rs11146701
rs1014718
rs1555070
All
0.000
0.001
0.002
0.003
0.004
0.005
MR leave−one−out sensitivity analysis for
'order.Bacteroidales.id.913' on 'UKB−b−14210'

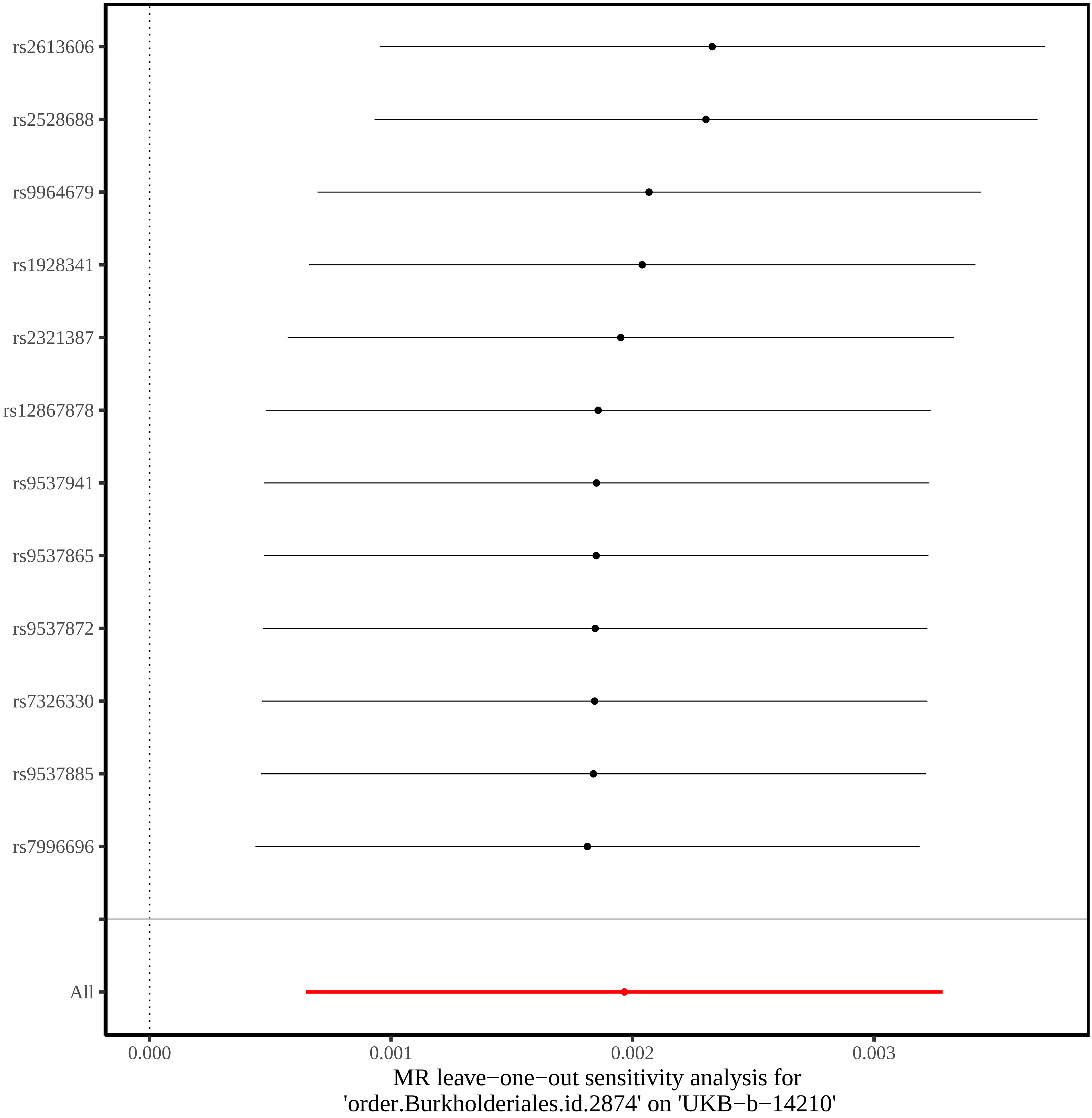

rs2613606
rs2528688
rs9964679
rs1928341
rs2321387
rs12867878
rs9537941
rs9537865
rs9537872
rs7326330
rs9537885
rs7996696
All
0.000
0.001
0.002
0.003
MR leave−one−out sensitivity analysis for
'order.Burkholderiales.id.2874' on 'UKB−b−14210'

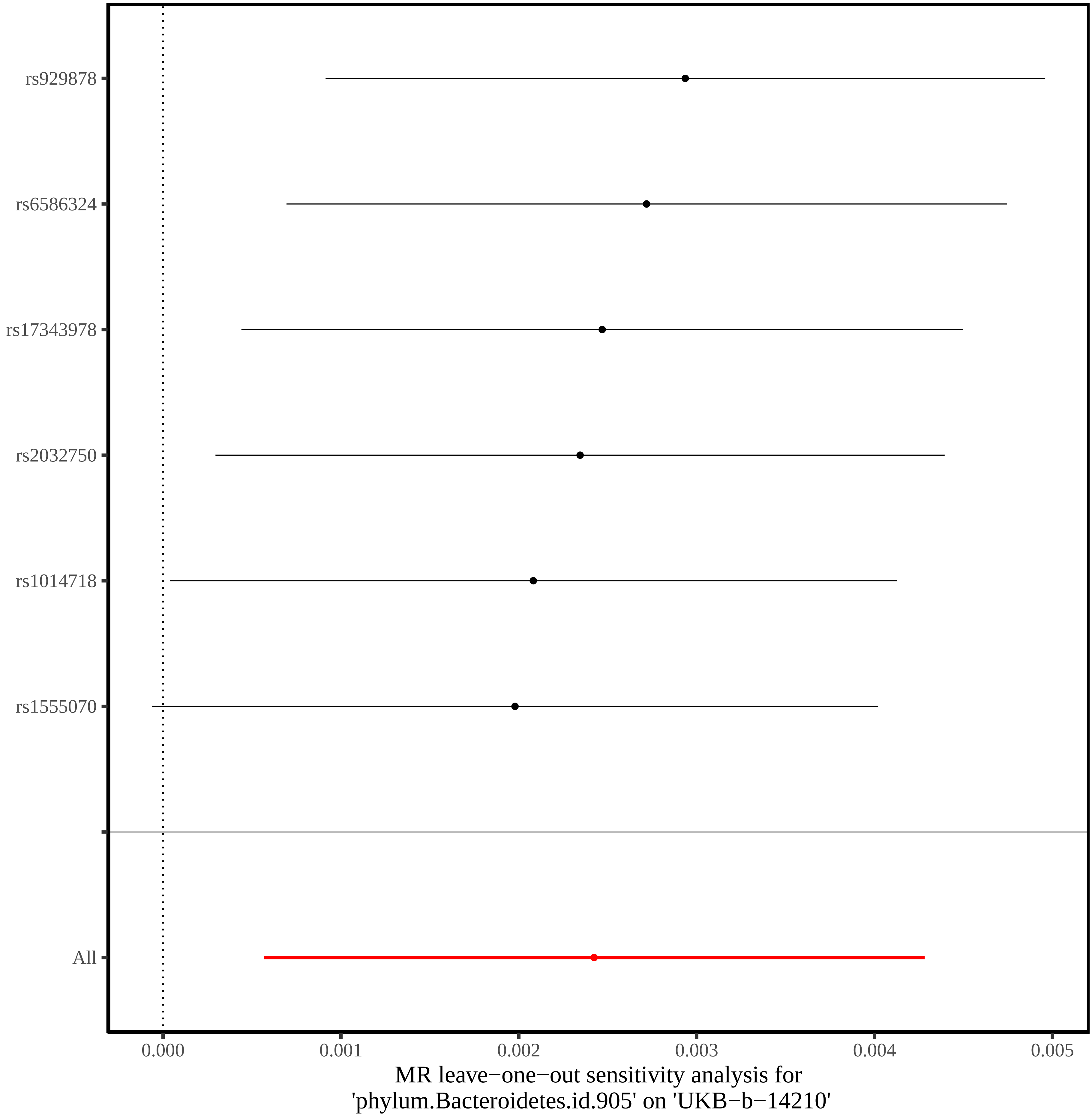

rs929878
rs6586324
rs17343978
rs2032750
rs1014718
rs1555070
All
0.000
0.001
0.002
0.003
0.004
0.005
MR leave−one−out sensitivity analysis for
'phylum.Bacteroidetes.id.905' on 'UKB−b−14210'

Supplementary Figure 5

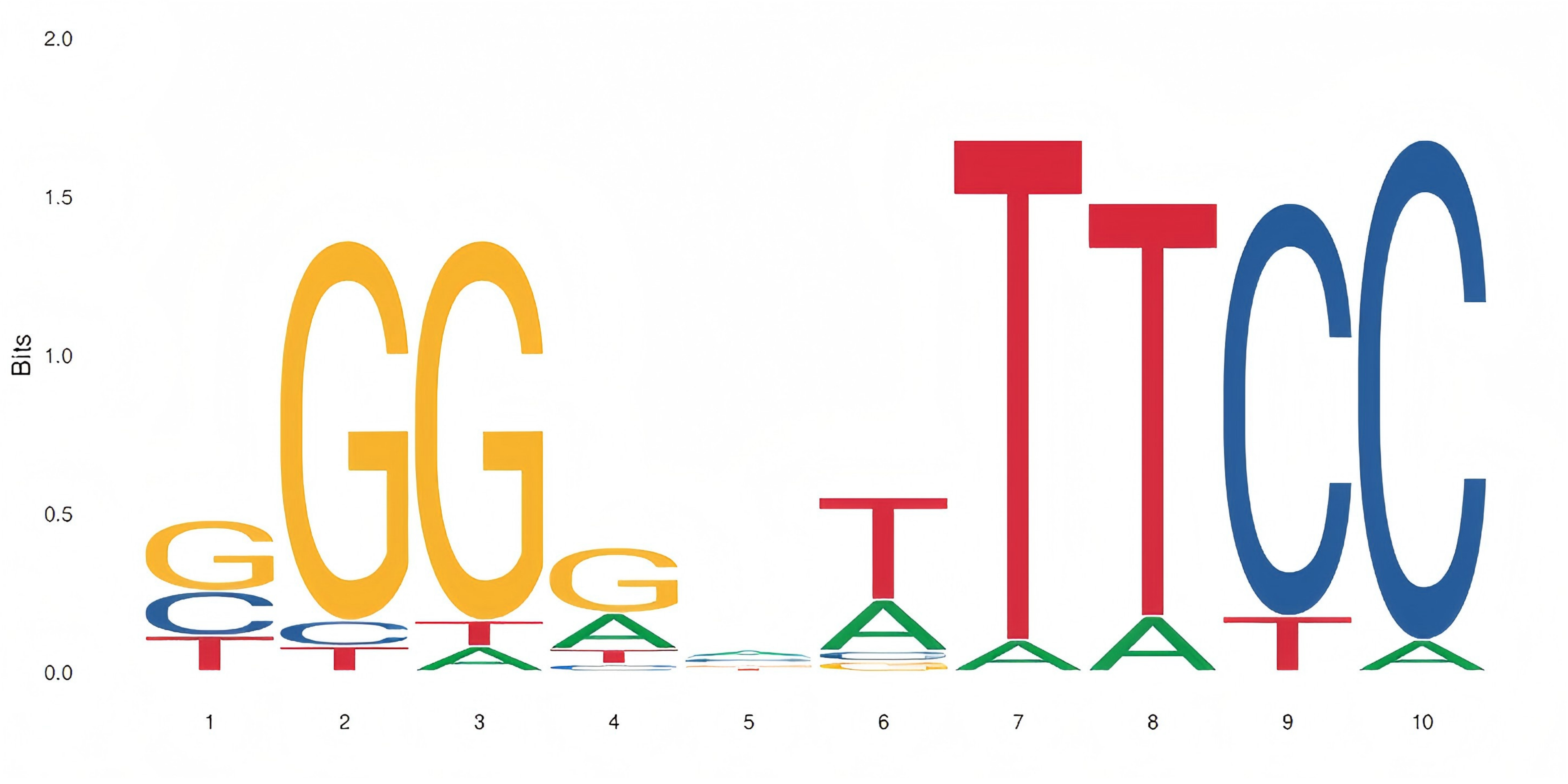

REL

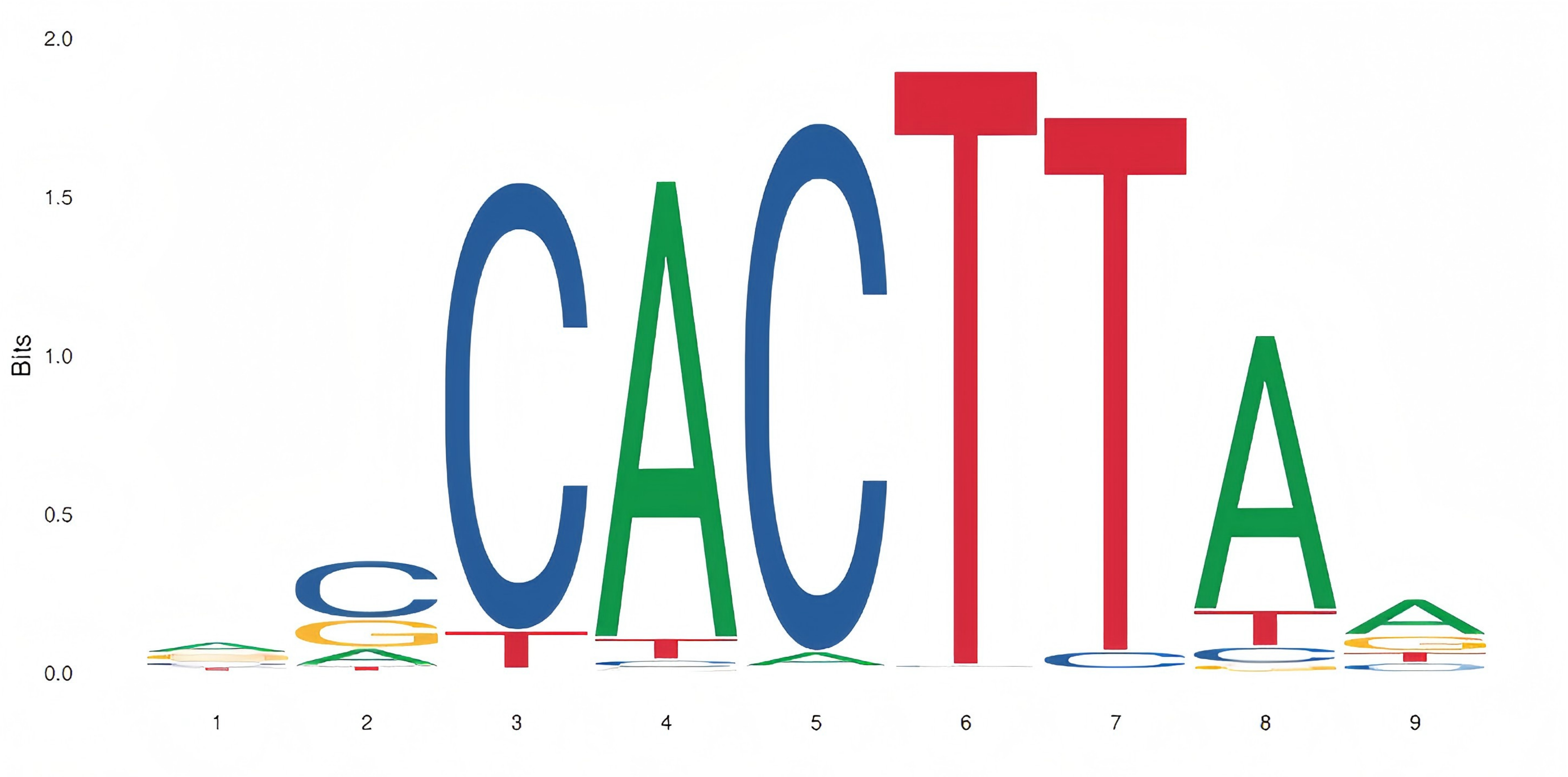

NKX3-2

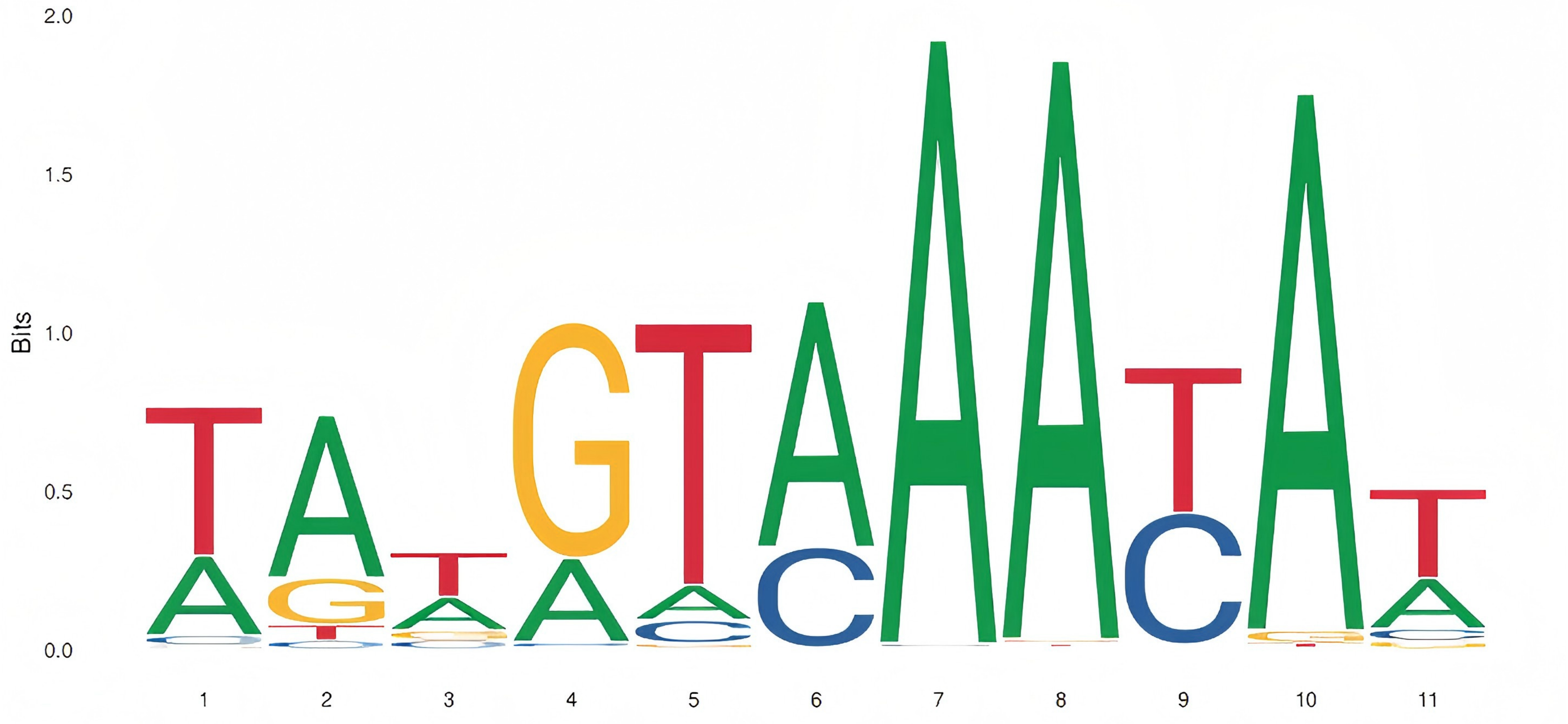

FOXC1

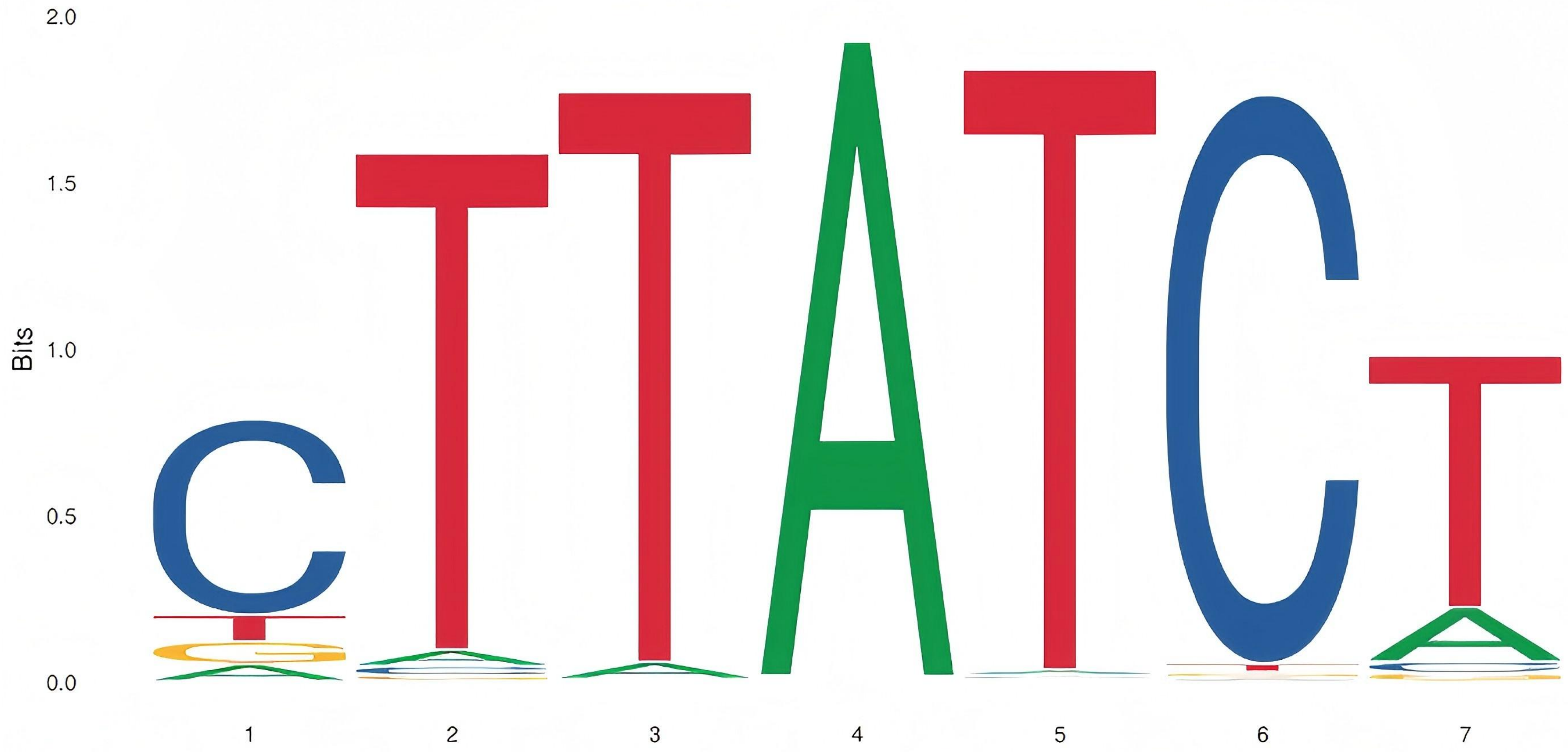

GATA2

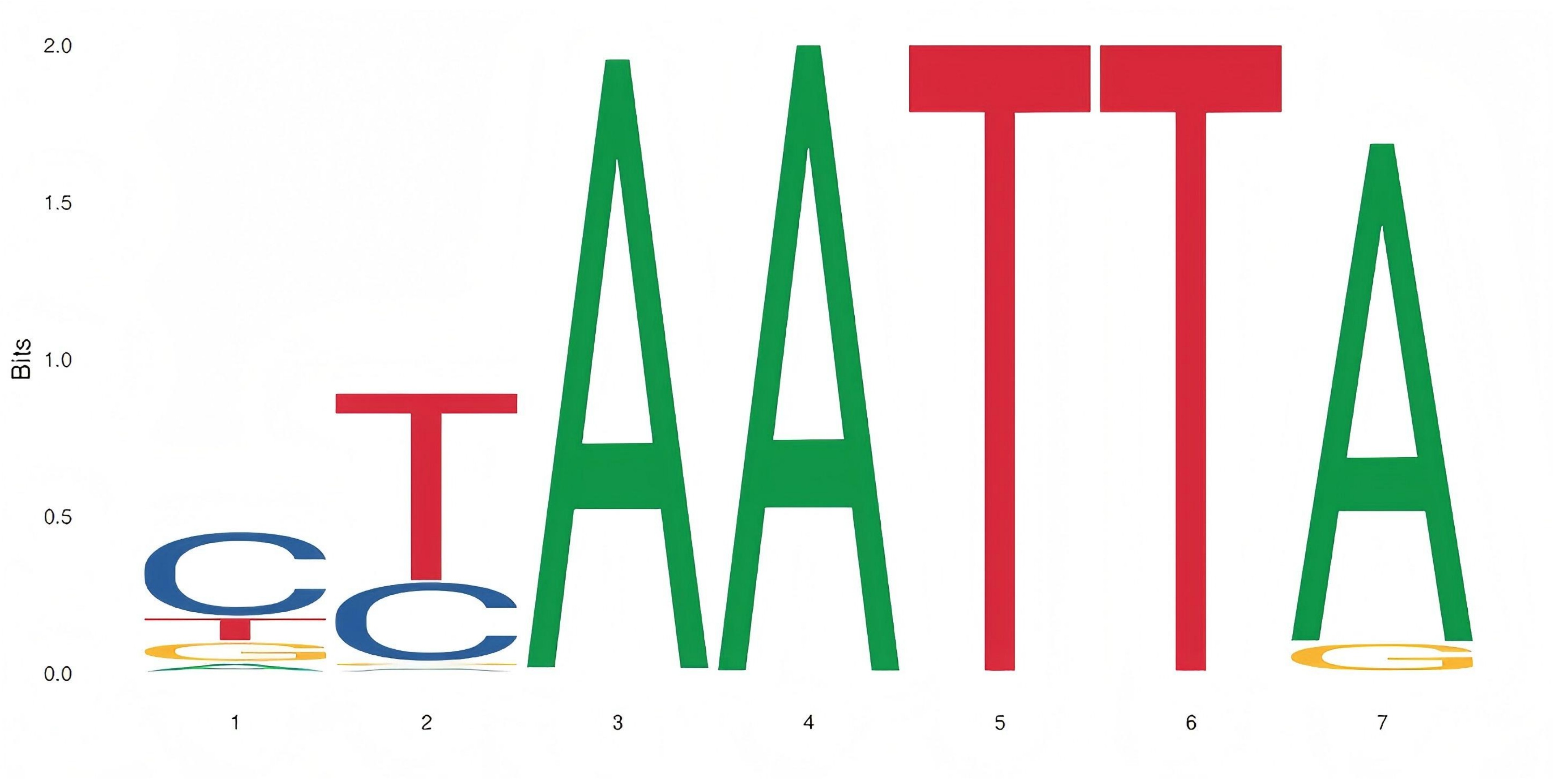

PRRX2

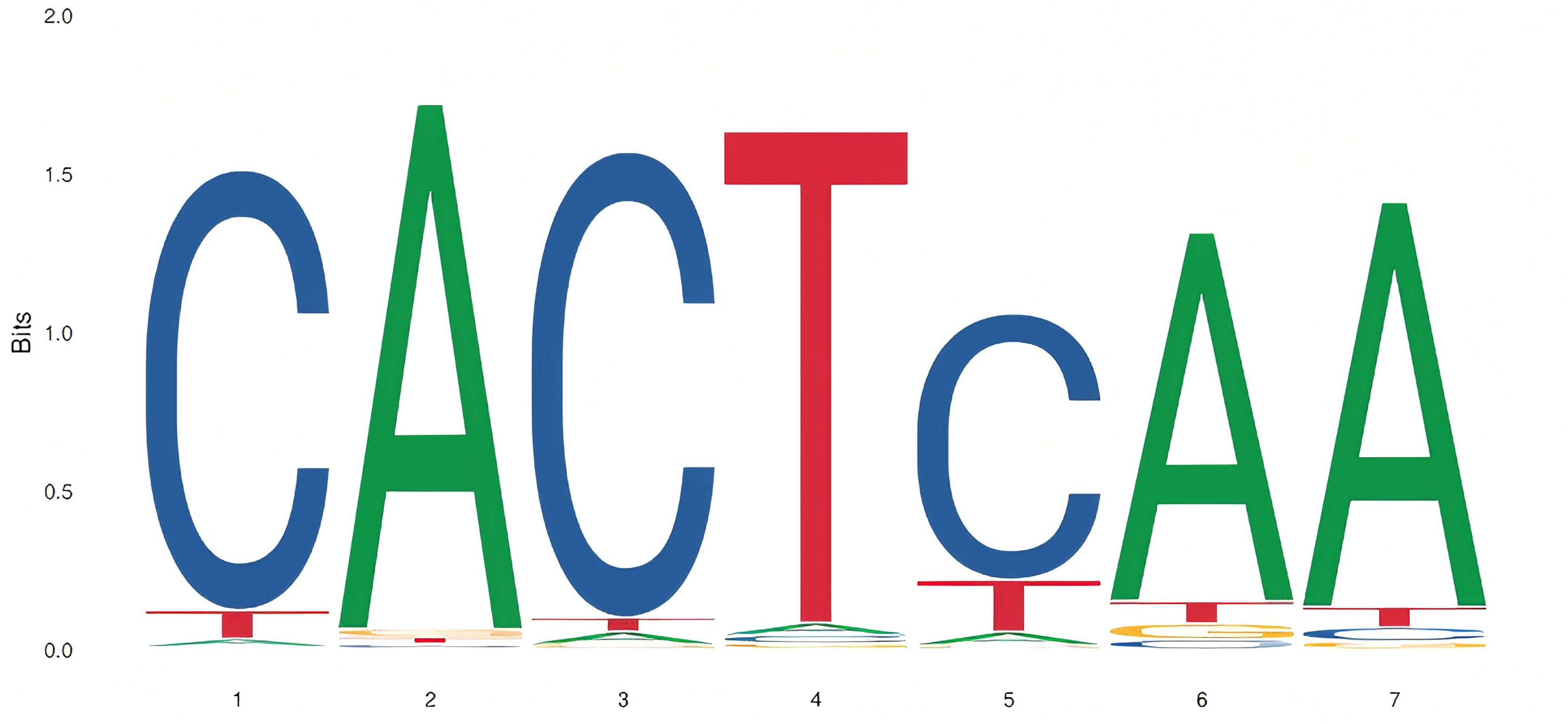

NKX2-5

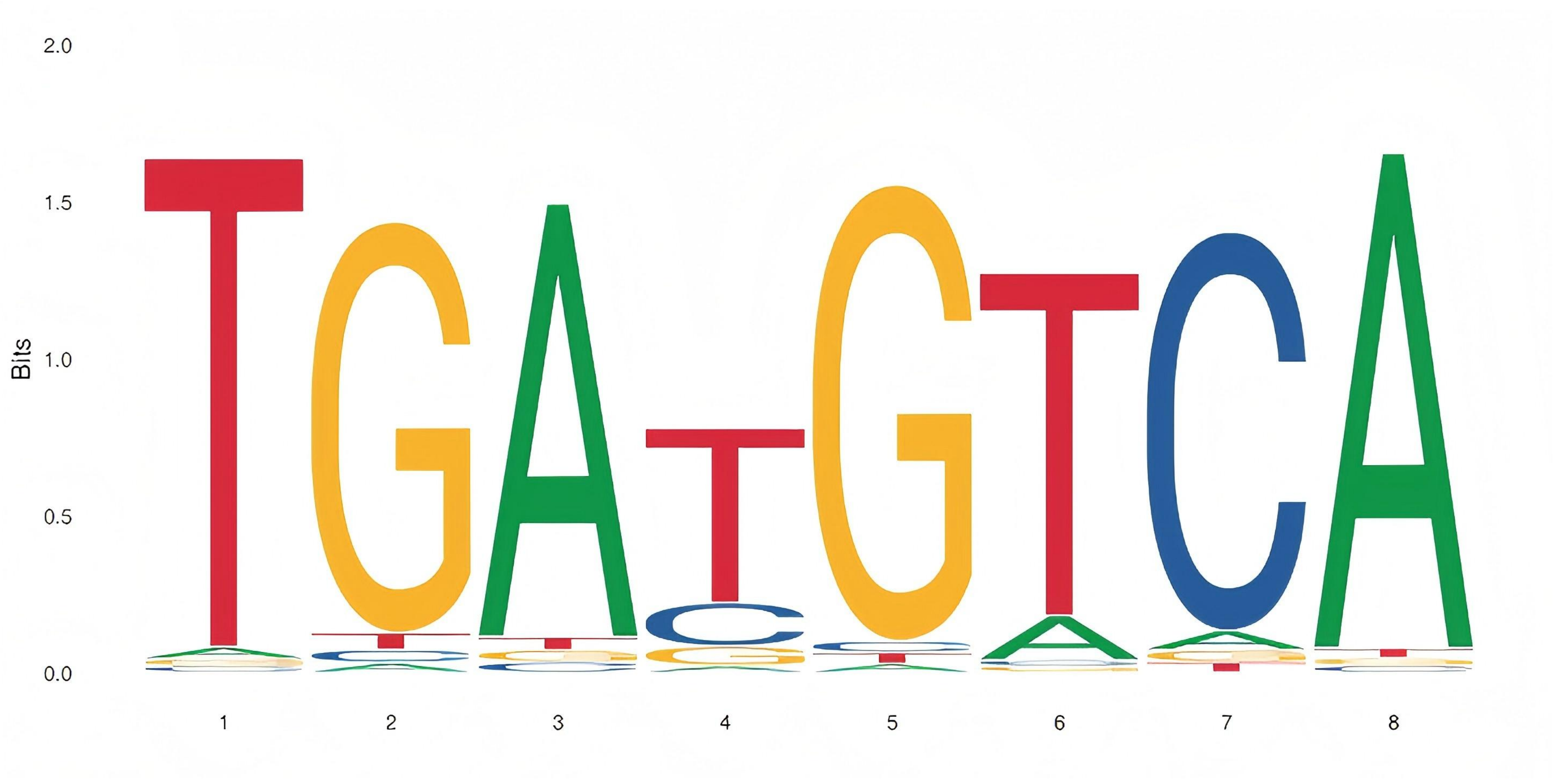

CREB1

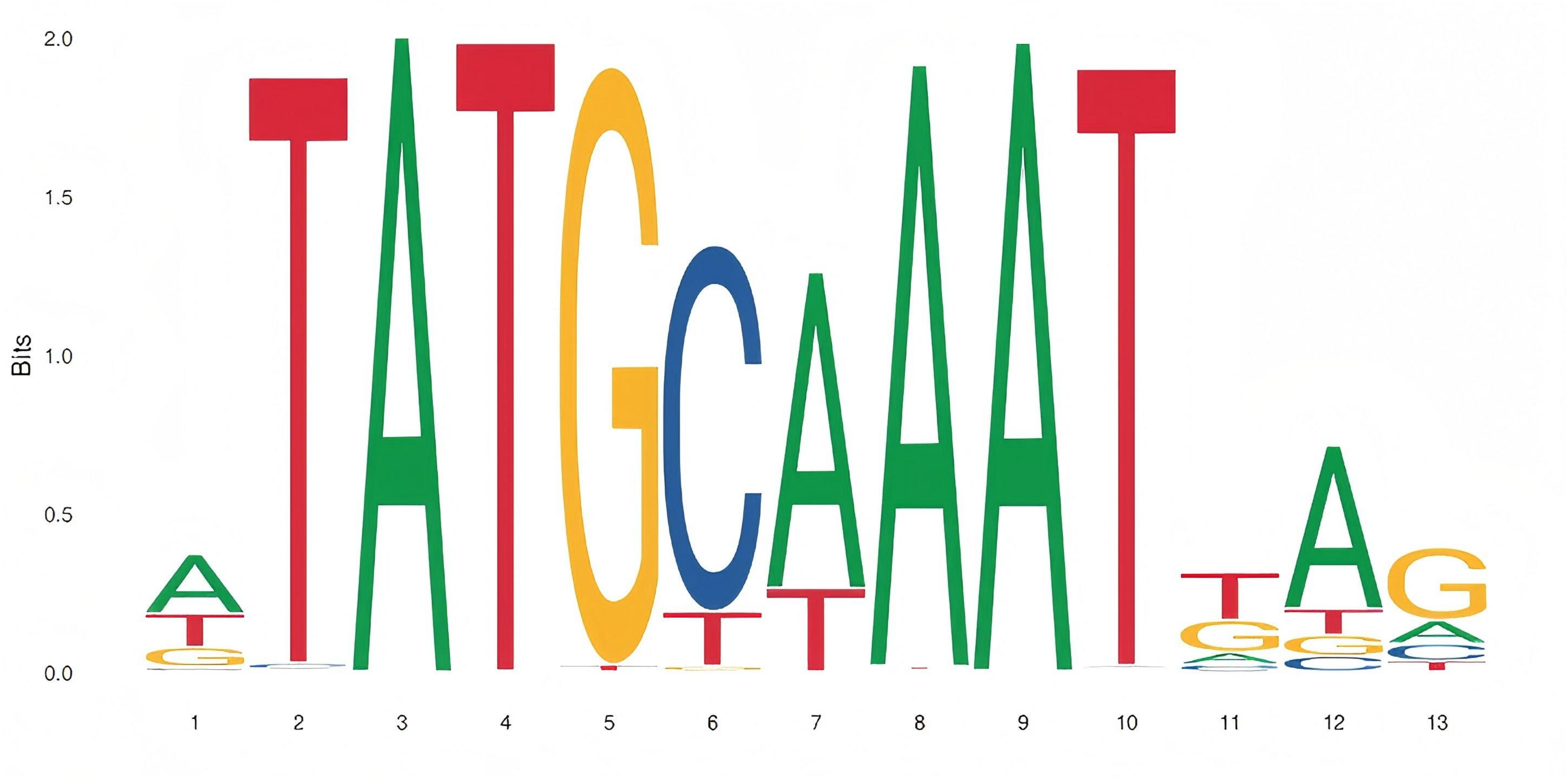

POU2F2

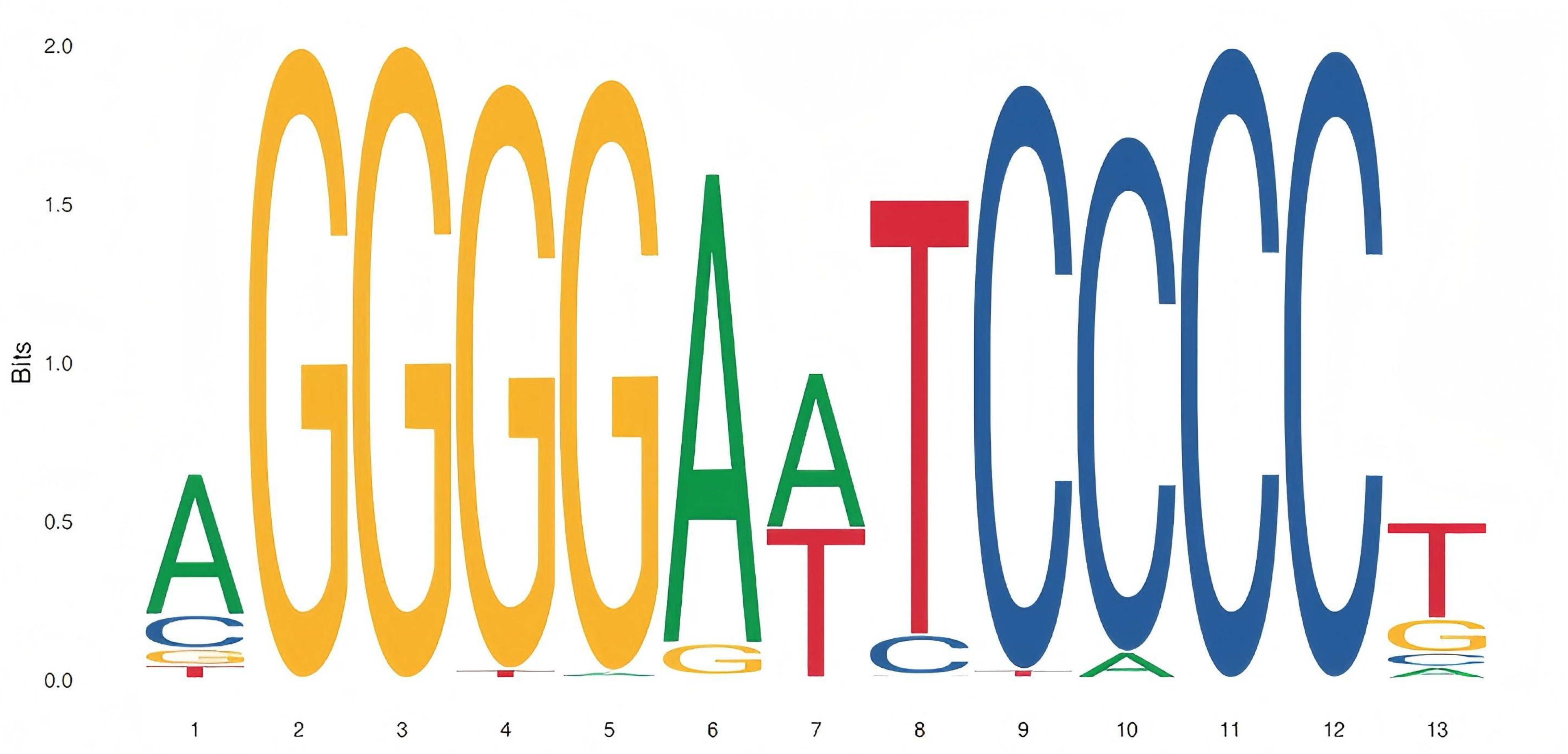

NFKB1

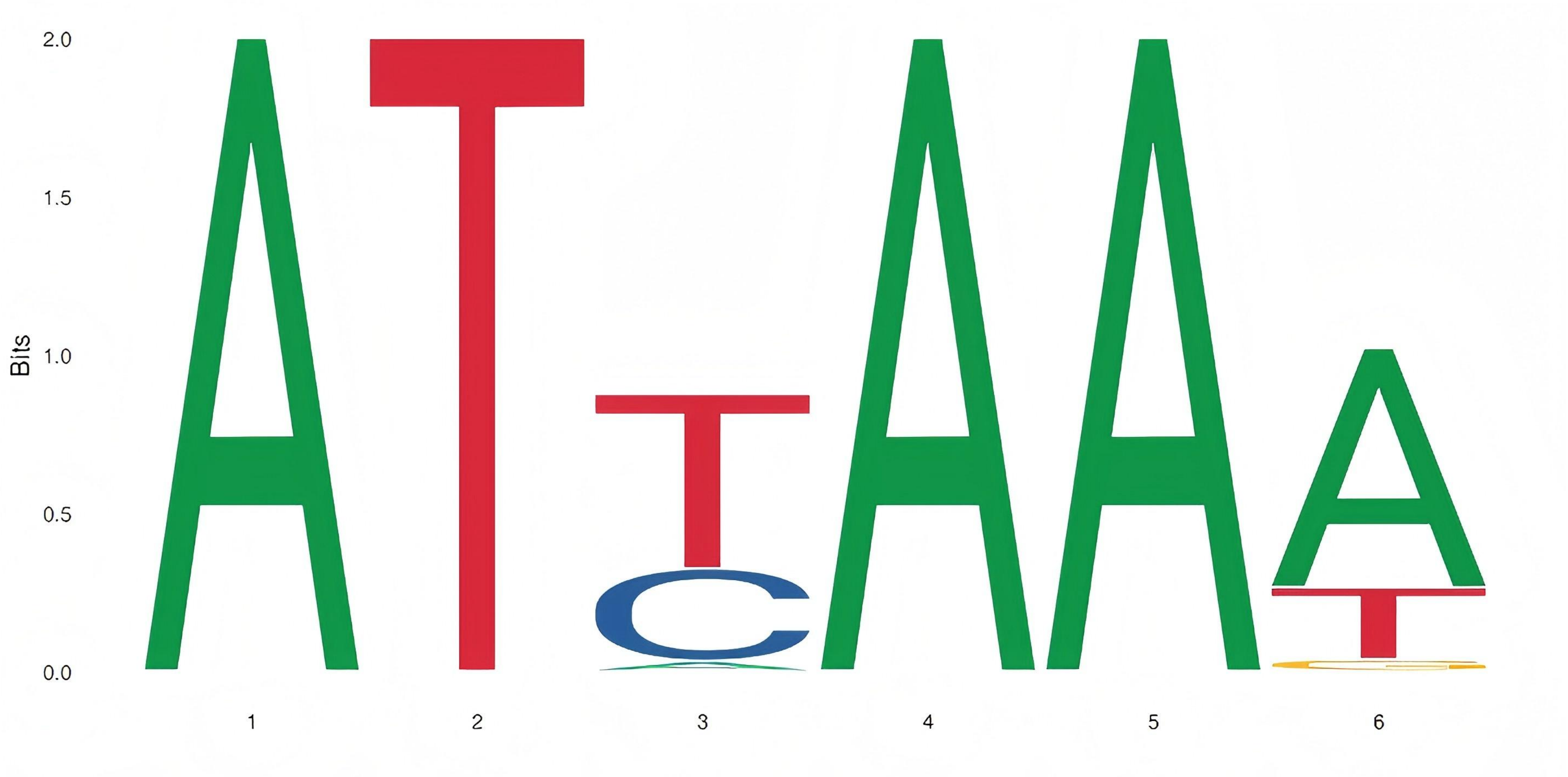

ARID3A
